# Movements of free-range pigs in rural communities in Zambia: an explorative study towards future ring interventions for the control of *Taenia solium*

**DOI:** 10.1186/s13071-022-05264-0

**Published:** 2022-04-27

**Authors:** Inge Van Damme, Ian Pray, Kabemba E. Mwape, Chiara Trevisan, Fien Coudenys, Chishimba Mubanga, Chembesofu Mwelwa, Victor Vaernewyck, Pierre Dorny, Seth E. O’Neal, Sarah Gabriël

**Affiliations:** 1 Laboratory of Foodborne Parasitic Zoonoses, Ghent University, Salisburylaan 133, 9820 Merelbeke, Belgium; 2 School of Public Health, Oregon Health & Science University–Portland State University, Portland, USA; 3 Department of Clinical Studies, School of Veterinary Medicine, University of Zambia, Lusaka, Zambia; 4 Department of Biomedical Sciences, Institute of Tropical Medicine, Antwerp, Belgium; 5 Center for Global Health–Tumbes, Universidad Peruana Cayetano Heredia, Lima, Peru

**Keywords:** Taeniosis, Cysticercosis, Control, GPS, Movement, *Sus scrofa*, Sub-Saharan Africa, Zambia, Ring treatment

## Abstract

**Background:**

*Taenia solium* typically affects resource-poor communities where pigs are allowed to roam freely, and sanitation and hygiene levels are suboptimal. Sustainable, long-term strategies are urgently needed to control the disease. Geographically targeted interventions, i.e. screening or treatment of taeniosis among people living near infected pigs (defined as ring screening and ring treatment, respectively), have been shown to be effective control options in Peru. However, these results might not be directly generalizable to sub-Saharan African settings. Pig movements play a vital role in the transmission and, consequently, the success of ring interventions against *T. solium*. The aim of the present study was to explore roaming patterns of pigs in *T. solium* endemic communities in Zambia as a first step toward evaluating whether ring interventions should be considered as a treatment option in Zambia.

**Methods:**

In total, 48 free-roaming pigs in two rural neighborhoods in the Eastern Province of Zambia were tracked using a Global Positioning System (GPS) receiver. Tracking took place in April (end of the rainy season) 2019 and October (end of the dry season) 2019. The number of revisitations and the time spent within rings of different radii (50, 100 and 250 m) around the coordinates of each pig owner’s household were calculated for each pig.

**Results:**

The total tracking time for 43 pigs in the final analysis set ranged between 43 and 94 h. Pigs spent a median of 31% and 13% of the tracked time outside the 50- and 100-m radius, respectively, although large variations were observed between pigs. Overall, 25 pigs (58%) went outside the 250-m ring at least once, and individual excursions lasting up to 16 h were observed. In the dry season, 17 out of 23 pigs went outside the 250-m radius compared to only eight out of 20 pigs in the rainy season (*P* = 0.014).

**Conclusions:**

In our study sites in Zambia, the majority of pigs spent most of their time within 50 or 100 m of their owner’s home, and these results are comparable with those on Peruvian pigs. Both radii could therefore be considered reasonable options in future ring interventions.

**Graphical Abstract:**

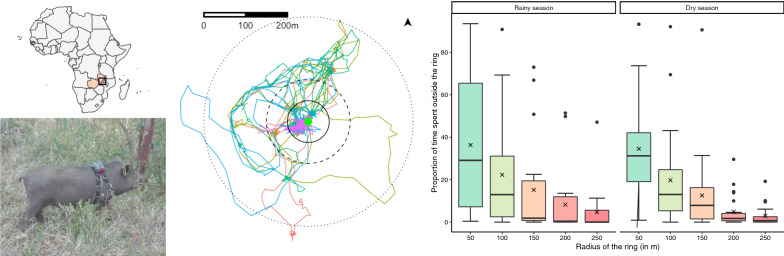

**Supplementary Information:**

The online version contains supplementary material available at 10.1186/s13071-022-05264-0.

## Background

People infected with the adult *Taenia solium* tapeworm can excrete high numbers of infective eggs in the stool [1]. When pigs ingest tapeworm eggs through coprophagia or eating contaminated food/water, the larvae migrate to tissues and organs where they mature into cysticerci (porcine cysticercosis). People who consume cysticerci in undercooked pork close the cycle by developing taeniosis. The main public health burden of *T. solium* results from humans possibly becoming accidental intermediate hosts after ingesting eggs. When the larvae migrate to the central nervous system, the infective person may cause neurocysticercosis, which is an important cause of acquired epilepsy in regions where *T. solium* is endemic [2]. Endemic regions are typically resource-poor communities where pigs are allowed to roam freely and sanitation and hygiene levels are suboptimal, conditions that occur in many rural areas of sub-Saharan Africa, Asia and Latin America.

Many intervention strategies targeting humans and/or pigs have been described to interrupt the transmission of *T. solium* [3]. Although recent studies carried out in endemic areas of Peru and Zambia showed that it is possible to eliminate active transmission by humans [4, 5], achieving this endpoint required multiple rounds of mass drug administration (MDA) at 4- or 6-monthly intervals combined with mass treatment/vaccination of pigs and public health education. Because the substantial resources required to achieve or maintain elimination may not be available in most endemic regions, efforts should be made to find effective and sustainable interventions to control the disease. Geographically targeted interventions among people living near visibly infected pigs and applying taeniosis treatment (defined as ring treatment) or taeniosis treatment after screening (ring screening) have been shown to be effective management/control options in Peru [6], where strong clustering of porcine cysticercosis around foci of human taeniosis has been demonstrated [7]. However, pig roaming behaviors may be different in other endemic regions of the world due to differences in housing density, geography, climate, agricultural practices, pig breeds and/or open defecation practices, and may consequently require differently sized rings.

Selecting an ideal radius for ring interventions is a balancing act. While larger rings are more likely to capture the source case of taeniosis, they also lose efficiency with increasing size of the targeted population and as the intervention approaches the scale of MDA. Smaller radius rings, on the other hand, may risk not capturing the source case of taeniosis. For the region in northern Peru, where the ring interventions were developed, 100-m rings were chosen as a convenient size for easy field implementation, and these were found to be effective in reducing the seroincidence in pigs when compared to mass applied interventions [6]. If large differences in the roaming patterns of pigs are present in Zambia compared to Peru, particularly in terms of time spent and frequency of visits outside of feasible ring radii, other control options might be more practical and effective. Knowledge of pig roaming behavior could also be used to tailor ring interventions to this area. Therefore, the aim of the present study was to explore the roaming patterns of pigs in *T.* *solium* endemic communities in Zambia as a first step toward evaluating whether ring interventions should be considered in Zambia.

## Methods

### Study area and population

The study was conducted in the Sinda and Katete Districts of the Eastern Province of Zambia (Additional file 1: Fig. S1), where *T. solium* is endemic [8]. Two rural neighborhoods, both of which were participating in a larger *T. solium* control trial [4], were selected for this study because of the presence of free-roaming pigs, open defecation practiced by the village inhabitants, village accessibility throughout the year and community willingness to participate. Pigs were selected in neighborhood A (elimination study arm with six 4-monthly human MDA and pig treatment/vaccination, followed by 6-monthly ring treatments) and neighborhood B (control study arm with yearly pig treatment) during two study time points of the ongoing trial. Pigs were tracked in April 2019, which is at the end of the rainy season (subsequently referred as the ‘rainy season,’ characterized by abundant foliage) and October 2019, which is at the end of the dry season (subsequently referred to as the ‘dry season,’ characterized by very little green foliage). At each time point, we approached all pig-owning households and requested consenting farmers to provide a pig from their household and include it in the study. Pigs that were aged ≥ 2 months, healthy and not in late gestation and which were allowed to roam freely (i.e. not routinely corralled or tethered) were eligible for inclusion. To obtain a representative sample, we aimed to include one eligible pig per pig-owning household. Pigs were selected based on convenience (presence around the household at the time of recruitment and ability to catch the pig) and size (according to the available sizes of harnesses).

### Pig tracking

Each pig received an ear tag with a unique identifier code and was fitted an adjustable nylon harness with an “iGotU” portable Global Positioning System (GPS) receiver (MobileAction Technology, New Taipei City, Taiwan) enclosed in a waterproof case (HPRC 1100; Plaber, Vicenza, Italy) [9] (Additional file 1: Fig. S2). The time the GPS device was turned on was recorded, along with the household code and the age and sex of the pig. Signal transmission took place once every minute, and pigs were continuously tracked for up to 4 days. Pigs were monitored daily and any interventions (e.g. harness adjustments) were recorded. On the agreed-upon final day, the starting time of the pursuit and the time the GPS device was turned off were recorded. GPS data on each pig were downloaded using @Trip and exported to CSV files.

### Data analysis

All pre-processing steps, visualizations and analyses of data were done using R version 4.1.2 [10]. Different pre-processing steps were conducted, with the aim to reach a balance between removing inaccurate data and overzealous rejection of valid animal movements [11]. The data from each pig were assessed by plotting observations and tracks on maps using the R package ggmap [12], and evident location errors (e.g. points outside the study area) were removed. Points with an unrealistic movement speed were identified using the R package *atlastools* [13], excluding points with an incoming or outgoing speed > 3 m/s [14]. Since data shortly after release and before capture may not properly represent the pig’s movement, the first 60 min and last 30 min were excluded. Additional temporal filters were used whenever an intervention was done (e.g. harness adjustments) by removing data for the time periods 30 min before and 30 after the intervention.

#### Calculation of revisitation metrics

The coordinates were projected from latitude–longitude to Universal Transverse Mercator (UTM) zone 36 south using spTransform from the *rgdal* package [15]. Revisitation metrics were calculated using the *recurse* package [16]. The residence time was determined for every visit outside rings of different radii (50, 100, 150, 200 and 250 m) around the coordinates of the pig-owning household (referred to hereafter as ’50-m ring’ up to ‘250-m ring’). Household coordinates were determined at the front door of the pig owner’s main building, collected during a baseline survey in 2015 [4] using a handheld GPS receiver (eTrex LegendH Cx; Garmin Ltd., Olathe, KS, USA). The proportion of time spent outside the ring was calculated by dividing the total time outside the ring by the total tracking time. The furthest straight-line distance from the household coordinate was also calculated.

#### Exploring the association of outcomes with predictor variables

The proportion of time spent outside the rings was analyzed for the 50- and 100-m rings using beta regressions [17]. Since a considerable number of pigs did not go outside the 250-m ring, going outside the 250-m ring (yes/no) was analyzed as a binary variable, using a logistic regression. The furthest distance traveled was analyzed using a generalized linear model with gamma distribution and identity link. The neighborhood, tracking season, pig sex and pig age (categorized as ≤ 6 months or > 6 months) were included as predictor variables in the full model, excluding at each step the predictor with the highest *P*-value until only significant variables remained. Neighborhood and season (including their interaction) were always included in the models to correct for the study design. For all models, marginal effects (predicted values) for the outcome variable were estimated using the function ggeffect [18]. Given the exploratory (hypothesis-generating) nature of this study and the relatively low sample size, a significance level of 10% was used for all tests.

## Results

### Pig characteristics

In total, 48 pigs, all of local breed, were enrolled in the study (12 per neighborhood per season). The results for five pigs were excluded due to confinement during the entire tracking time (*n* = 3; rainy season in neighborhood A) or due to loss of the GPS device (*n* = 2; 1 in the dry season in neighborhood A, and 1 in the rainy season in neighborhood B). The data of 20 pigs tracked in the rainy season and 23 pigs tracked in the dry season were used in the final analyses (Table 1; Additional file 2). Most pigs were female (35/43, 81%), and age varied between 3 and 71 months (median age: 10 months). The pigs included in the final analysis set originated from 19 different households in the rainy season and 22 different households in the dry season. Two pigs originated from the same household in the rainy season, and two pigs were from the same household during dry season. From nine households, one pig was included in both seasons (4 in neighborhood A, 5 in neighborhood B). The tracking time on the pigs included in the final analysis ranged from 44 to 95 h (Table 1).

**Table 1:** Characteristics and tracking information of pigs included in the final analysis (*n* = 43)

Characteristics and tracking information of pigs	Rainy season		Dry season	
	Neighborhood A (*n* = 9)	Neighborhood B (*n* = 11)	Neighborhood A (*n* = 11)	Neighborhood B (*n* = 12)
*Sex*,* n*				
Female	6	10	10	9
Male	3	1	1	3
*Age* (months),* n*				
3–6	1	2	4	5
7–11	6	3	3	1
12–24	2	3	4	3
> 24	0	3	0	3
*Tracking information*				
Median tracking time, h (minimum–maximum)^a^	74 (64–78)	70 (44–91)	72 (65–77)	93 (68–95)
Median number of observations^a^	4339 (3890–4481)	4046 (2631–5354)	4366 (3446–4626)	5424 (4062–5674)
Total number of observations used for analysis	38,507	46,290	47,067	63,202

^a^Based on entries that were used in the final analysis set (after cleaning)

### Pig tracking results

**Fig. 1:**
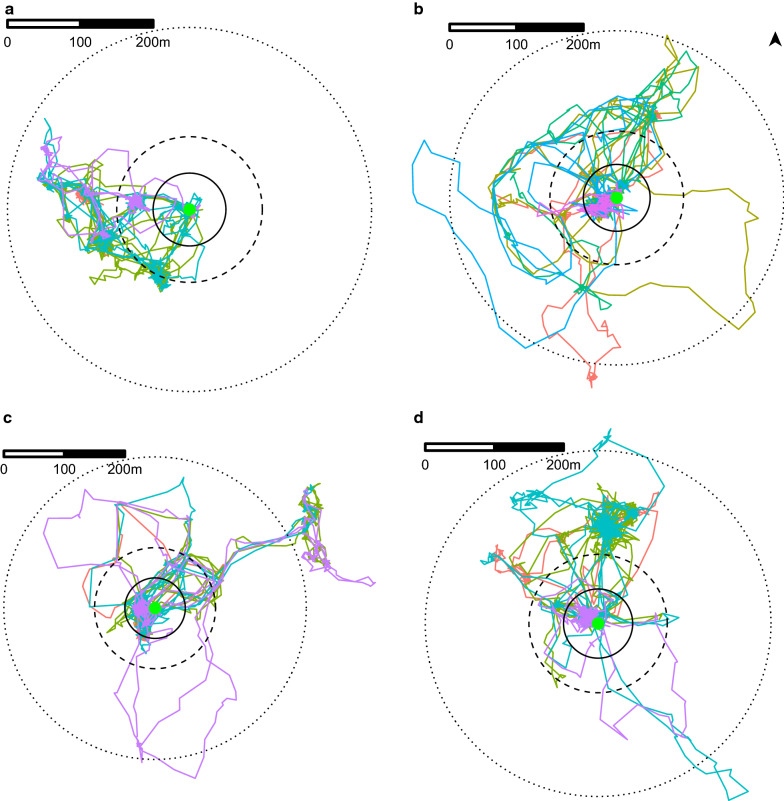
Visualization of tracking results for four different pigs (**a**–**d**). The circles represent rings around the pig-owning household (green central point), with the inner circle representing the ring with radius 50 m ring, the middle circle represent the ring with radius 100 m and the outer circle representing the ring with radius 250 m. **a **Pig spent 88, 55 and 0% of its time outside the 50-, 100- and 250-m rings, respectively. **b** Pig spent 19, 15 and 1% of its time outside the 50-, 100- and 250-m rings, respectively. **c** Pig spent 22, 16 and 11% of its time outside the 50-, 100- and 250-m rings, respectively. **d** Pig spent 73, 68 and 1% of its time outside the 50-, 100- and 250-m rings, respectively. Each color represents a different day

**Fig. 2:**
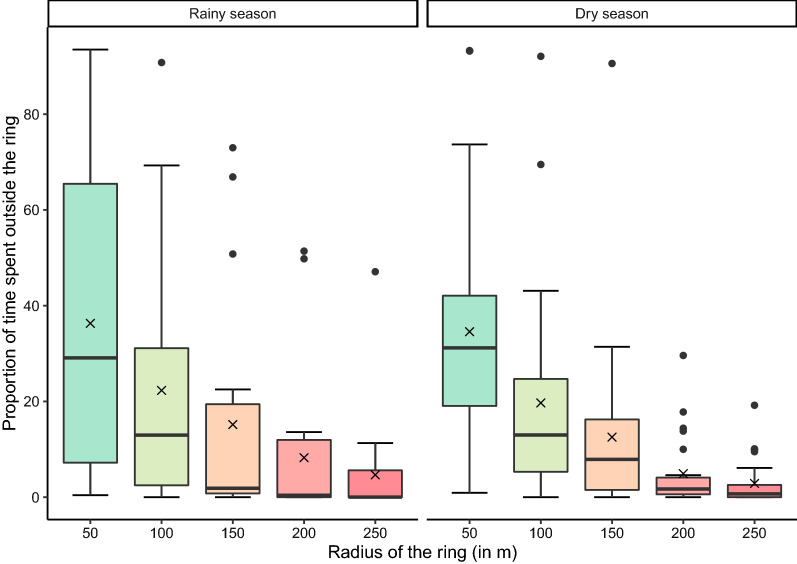
Proportion of the tracking time pigs spent outside differently sized rings around their household (*n* = 43 pigs). The cross-mark (x) represents the mean value, the horizontal line in the box represents the median value, the lower and upper boundaries of the box represent the first and third quartiles, respectively. The whiskers extend to the largest value no further than 1.5-fold the inter-quartile range, and the individual dots represent observations above the latter value

As an illustration, tracking results from four different pigs are visualized in Fig. 1. A summary of the proportion of time spent outside the different rings is shown in Fig. 2. A detailed summary of these visualizations are provided in Additional file 1: Fig. S3.

None of the pigs stayed within the 50-m ring around their household throughout the entire tracking period. Overall, pigs spent a median of 31% of the tracked time outside the ring with 50-m radius, although large variations were observed between pigs (range between < 1% and 93%; Fig. 2). The longest visit outside the 50-m ring varied between pigs, ranging from 13 min to 18 h (median: 5 h). No significant differences were found in the length of time outside the 50-m ring between neighborhoods, seasons, sexes and ages (*P* > 0.10; Additional file 1: Table S1).

Most pigs went outside the 100-m ring, with only two pigs (5%) staying within the 100-m radius around the household during the entire tracking period. Overall, the median time spent outside the 100-m radius was 13%, ranging from < 1% to 92%. Six pigs spent more than half of their time outside their 100-m ring. Older pigs spent more time outside the 100-m ring compared to younger pigs (*P* = 0.088), with predicted marginal mean times of 25% (18–34%) and 15% (8–26%), respectively. No significant differences were found between neighborhoods, seasons and pig sexes (*P* > 0.10; Additional file 1: Table S2). The median duration of a visit outside the 100-m ring varied between pigs, ranging from 1 to 247 min (median: 10 min), and the longest individual visit lasted for 1232 min (> 20 h). The visits outside the 100-m ring showed a bimodal distribution throughout the day, particularly in the dry season, with most visits occurring around 5 a.m. and 4 p.m. (Additional file 1: Fig. S4).

Overall, 25 pigs (58%) went outside the 250-m ring at least once. Although half of these pigs spent only ≤ 2% of their time outside this ring, one pig spent 47% of its time outside this ring. The median duration of a visit outside the 250-m ring varied between pigs, ranging from 2 to 214 min (median: 9 min), and individual visits up to 966 min (16 h) were observed.

In the dry season, 17 out of 23 pigs went outside the 250-m ring compared to only eight out of 20 pigs in the rainy season. In neighborhood A, the probability of traveling outside the 250-m ring was 82% (95% confidence interval [CI]: 49–95%) in the dry season and 22% (95% CI: 6–58%) in the rainy season; in neighborhood B, the estimated probabilities were respectively 67% (95% CI: 38–87%) in the dry season and 55% (05% CI: 27–80%) in the rainy season (Additional file 1: Table S3).

On average, the maximum straight-line distance traveled away from the household during the tracking period was 280 m (median value; minimum: 100 m, maximum: 855 m). Two pigs traveled more than 800 m away from their respective owner’s household. The maximum distance traveled was associated with the age of the pig, season and neighborhood (Additional file 1: Table S4). The predicted marginal maximum distance was 335 m (95% CI: 277–393 m) for older pigs, compared to 239 m (95% CI: 172–307 m) for younger pigs.

## Discussion

In this study, we tracked pigs with the aim to explore their movements within and outside rings of different radii around their owners’ household, as a first step towards developing ring treatment strategies for the control of *T. solium* in rural communities in Zambia. We found that the majority of pigs spent most of their time within a 50-m radius and a 100-m radius around their owner’s home (median proportion of time: 69% and 87%, respectively). This is very similar to the results reported by Pray and colleagues [9], who found that pigs in Peru spent a median of 70% and 83% of their roaming time within a 50- and 100-m radius, respectively, of their homes. Since 100-m ring interventions were shown to be efficient in Peru [6], the similarity in residence time between our findings and those in Peru [9] indicates that a 100-m ring intervention may also be effective in sub-Saharan rural settings.

The majority of pigs (95%) in our study roamed outside the 100-m ring around their homes at least once, and more than half of the pigs traveled > 250 m away from their home, providing ample opportunity to become infected beyond the 100-m ring. If a pig were to become infected when outside the 100-m ring around their home, the source case of taeniosis would not be identified or treated. Consequently, our results advocate for increasing the ring radius to reach the desired control effect. Nevertheless, this study was limited to observing pig roaming patterns, which may not be indicative of their exposure to *T. solium* eggs and, consequently, to the risk of infection. Many other factors could influence infection risk of pigs and the potential effectiveness of ring interventions.

Factors that influence pig movement can vary between regions and even between villages in the same region. We found that pigs were more likely to roam outside the 250-m ring in the dry season than in the rainy season. This difference could be related to the lower availability of food in the dry season driving the need to roam further from home. Similar patterns were observed in rural Kenya, where larger (non-significant) home ranges were observed in the dry season compared to the rainy season [19]. In contrast, in Peru, almost all occurrences of extended roaming were observed in the rainy season, and home ranges of Peruvian pigs were 61% larger during the rainy season than in the dry season [14]. Our study was limited to two time points, which were at the end of each season (dry and rainy), so the observed tracks may not be representative of the entire dry/rainy season. We also found some indications that older pigs travel longer distances than younger pigs, and roamed more frequently outside the 100-m ring. In Peru, pigs that roam long distances had more contact with areas of open defecation and thus represent an important subgroup in the context of interventions to control *T. solium* [14]. Differences in pig roaming behavior were also observed between villages in the same region in Peru [14]. Likewise, in the present study, there were some indications that in the rainy season pigs in neighborhood A traveled less far than pigs in neighborhood B, possibly due to the closer proximity of the households within the community in neighborhood A. In the latter neighborhood, one round of ring strategy with a few 100-m rings would approximate an MDA. Therefore, defining the optimal ring size is a balance between the ring being sufficiently large to obtain the desired effectiveness, while also being the smallest possible to increase efficiency. Assuming that there is a relation between residence time and the risk of infection, the 50-m ring could potentially be a worthy alternative.

This was an exploratory study with a number of limitations. Although active foraging time may be a better proxy for the infection risk with *T. solium*, we did not exclude night or inactive time points from our analysis, as several pigs in our study roamed during the night, and GPS data are not reliable to assess nighttime activity [20]. The use of collar-mounted activity sensors or visual observations may be useful to provide more insights into animal activity and behavior, including the assessment of pig exposure to human stool and the *T. solium* infection risk. The pigs in our sample could be weaker and smaller than the total pig population in Zambia as they were selected based on their size (according to the available harnesses) and convenience (opportunity to catch the pigs), potentially resulting in a sample of weaker, smaller pigs, and those who were at home at the time of enrollment (i.e. less likely to be roamers). Given the relatively short tracking time of certain pigs (< 48 h), we also cannot completely rule out the possibility that capture, harness or unforeseen events may have affected the pigs’ normal behavior. Although the relatively small sample size and inclusion of only two neighborhoods, which were both part of an ongoing intervention trial, limits the strength of conclusions that can be drawn, this study is important in that it contributes to the necessary knowledge base that is needed for testing and tailoring ring interventions to this new setting.

## Conclusions

In our study sites in Zambia, the majority of pigs spent most of their time within a 50- or 100-m radius of their owner’s home. Our data support the previously proposed 100-m ring interventions, although a 50-m radius may also be considered reasonable in future interventional trials. While studies to evaluate the strength and extent of clustering of human taeniosis and porcine cysticercosis could provide additional information [7], such an approach requires complete screening of entire villages for taeniosis, as well as the purchase and necropsy of all pigs, both of which are expensive endeavors. Resources might better be applied towards the completion of an intervention trial, as this is ultimately needed to determine whether ring interventions can achieve the right combination of effectiveness and efficacy in Zambia.

## Supplementary Information

**Additional file 1: Fig. S1.** Map of the study areas. **Fig. S2.** Pig with a GPS device in a waterproof case, secured to a harness for tracking. **Fig. S3.** Boxplots of (A) the proportion of time and (B) the number of visits outside different rings around the household. **Fig. S4.** Number of visits outside the 100-m and 250-m ring according to the hour of the day. **Table S1.** Model output for the proportion of time outside the 50-m ring. **Table S2.** Model output for the proportion of time outside the 100-m ring. **Table S3.** Model output for going outside the 250-m ring. **Table S4.** Model output for the maximum distance from home.

**Additional file 2.** GPS tracks and summary data on individual pigs.

## Data Availability

The individual data points, tracks and summary statistics per pig are provided in Additional file 2: GPS tracks and summary data of individual pigs. Aggregated data can be accessed upon request via the Institute of Tropical Medicine data access committee.

## Abbreviations

GPS: Global positioning system
MDA: Mass drug administration
