# Supplementary material for: Movements of free-range pigs in rural communities in Zambia: an explorative study towards future ring interventions for the control of Taenia solium
Source: Parasit Vectors. 2022 Apr 27;15:150. doi: 10.1186/s13071-022-05264-0 (PMC9044682; doi:10.1186/s13071-022-05264-0)
Supplement: Supplementary file 1 — Additional file 1: Fig. S1. Map of the study areas. Fig. S2. Pig with a GPS device in a waterproof case, secured to a harness for tracking. Fig. S3. Boxplots of (A) the proportion of time and (B) the number of visits outside different rings around the household. Fig. S4. Number of visits outside the 100-m and 250-m ring according to the hour of the day. Table S1. Model output for the proportion of time outside the 50-m ring. Table S2. Model output for the proportion of time outside the 100-m ring. Table S3. Model output for going outside the 250-m ring. Table S4. Model output for the maximum distance from home. [file 13071_2022_5264_MOESM1_ESM.pdf]

Additional file 1

This file accompanies the manuscript "Movements of free-range pigs in rural communities in Zambia: an explorative study towards future ring interventions for the control of *Taenia solium*".

Table of contents

Figure S1. Map of the study areas. 2

Figure S2. Pig with a GPS device in a waterproof case, secured to a harness for tracking. 3

Figure S3. Boxplots of (A) the proportion of time and (B) the number of visits outside different rings around the household. 4

Figure S4. Number of visits outside the 100-m and 250-m ring according to the hour of the day. 5

Table S1. Model output for the proportion of time outside the 50m ring. 6

Table S2. Model output for the proportion of time outside the 100m ring. 7

Table S3. Model output for going outside the 250m ring. 8

Table S4. Model output for the maximum distance from home. 9

Additional file 1

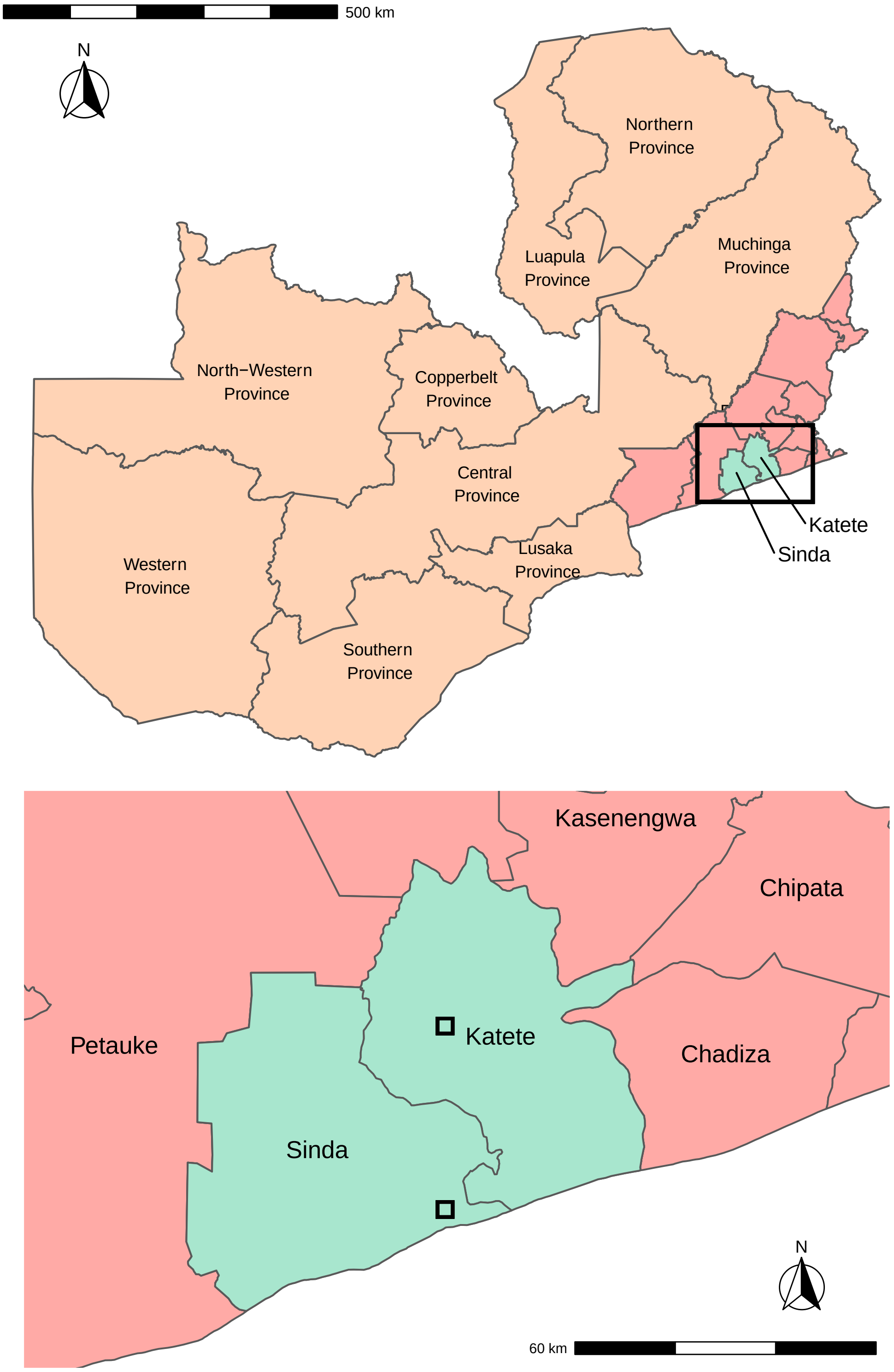

Figure S1. Map of the study areas.

The study areas are located in Sinda and Katete district (green), located in Eastern Province (red) of Zambia.

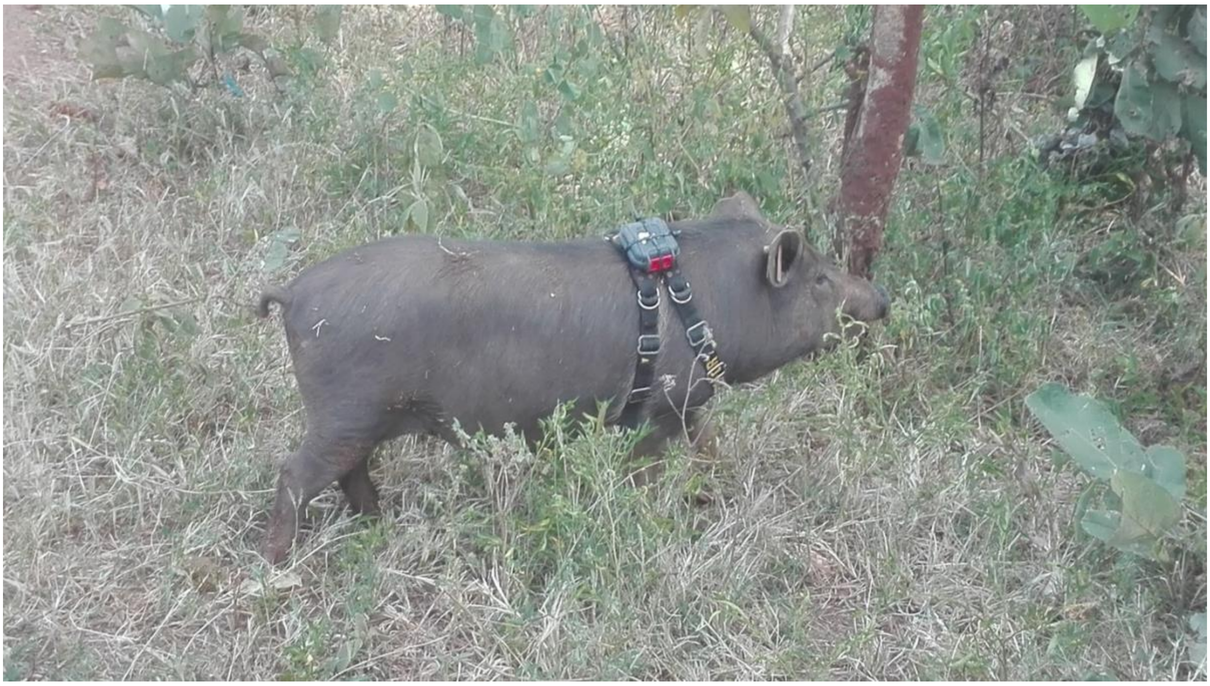

Figure S2. Pig with a GPS device in a waterproof case, secured to a harness for tracking.

Additional file 1

A

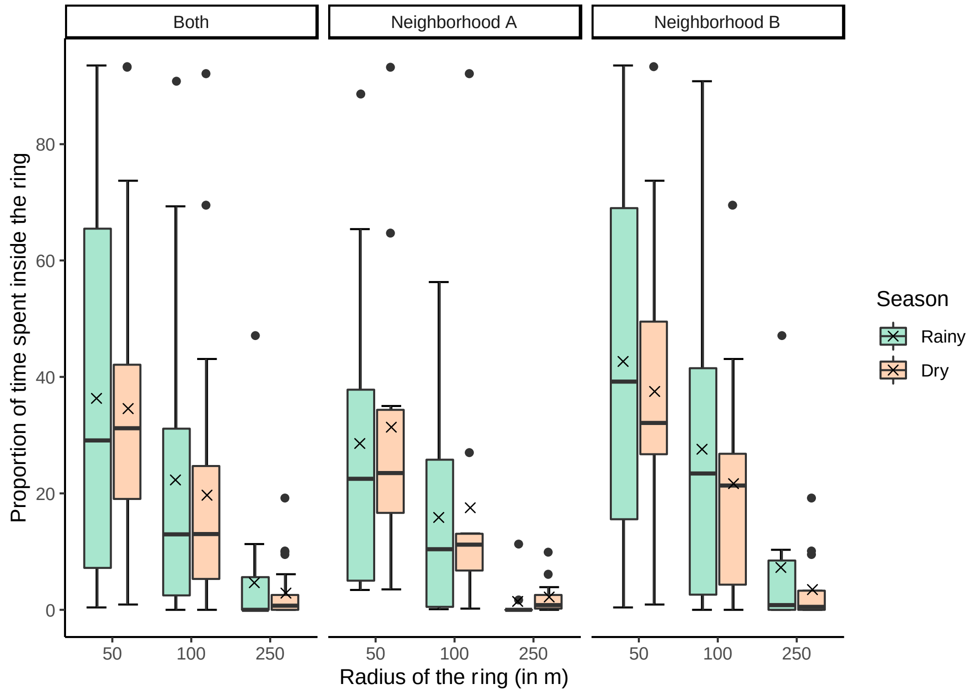

B

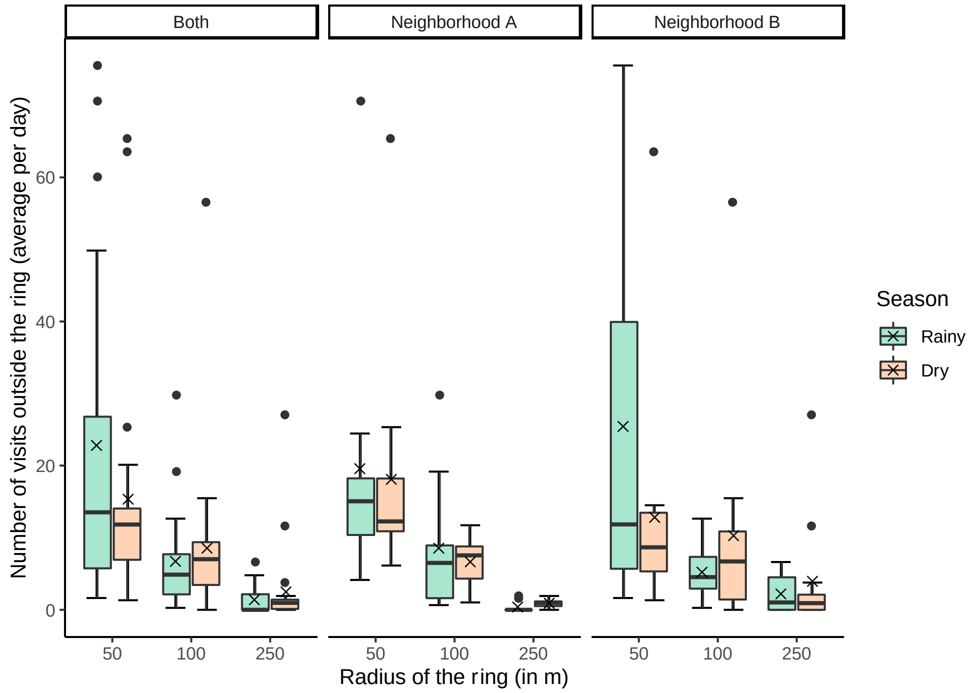

Figure S3. Boxplots of (A) the proportion of time and (B) the number of visits outside different rings around the household.

Results are presented for both neighborhoods, per season (rainy season, n = 20 pigs; dry season, n = 23 pigs). The horizontal line in the box represents the median value, the lower and upper hinges the first and third quartiles. The whiskers extend to the largest value no further than 1.5 times the inter-quartile range and the individual dots represent observations above the latter value. The mean is indicated with a cross-mark (x).

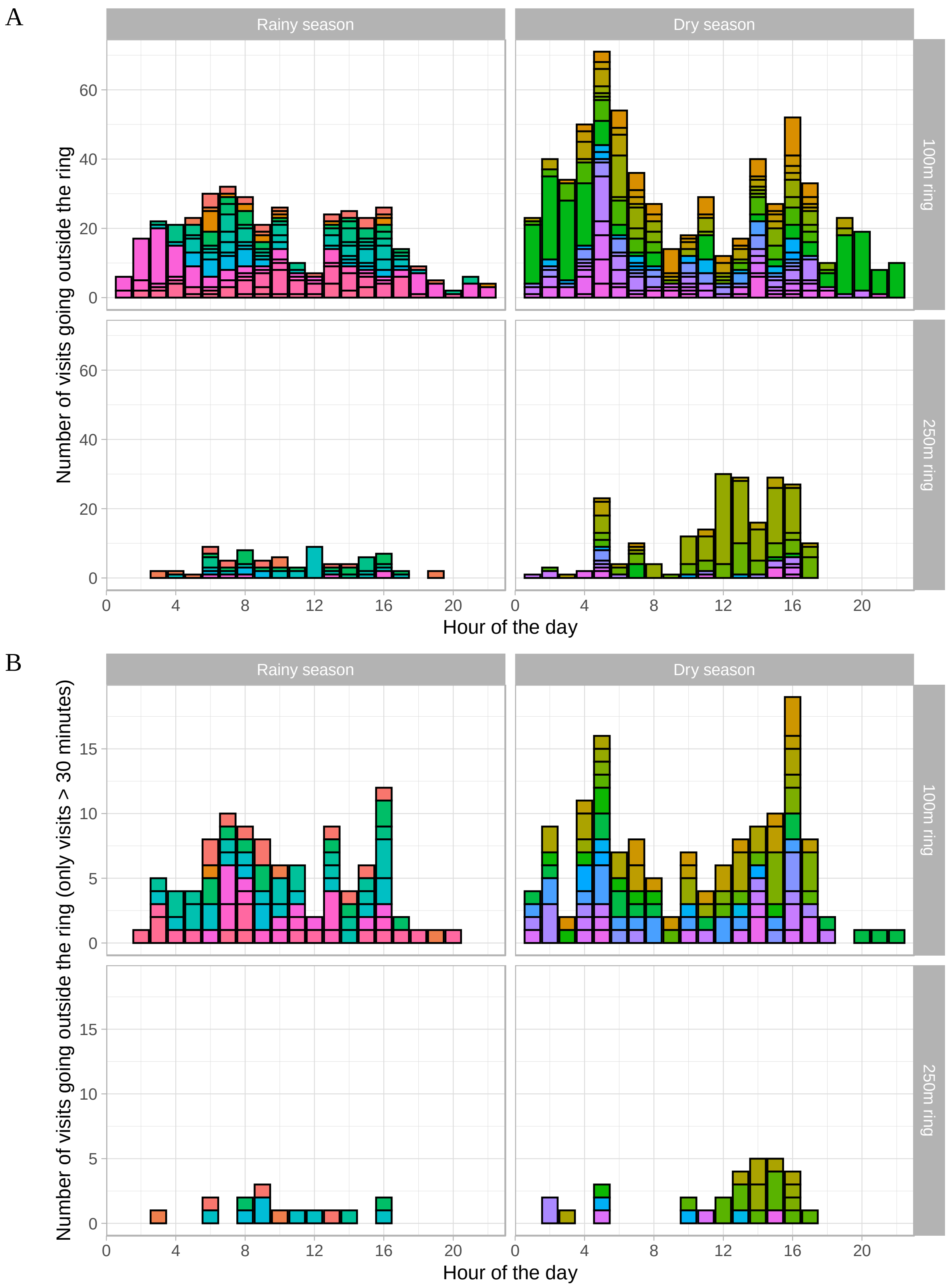

Figure S4. Number of visits outside the 100-m and 250-m ring according to the hour of the day.

A: including all visits; B: including only visits with a duration > 30 minutes (cutoff value chosen arbitrarily to exclude short visits, which may be caused by a track close to the ring). The hour of the day (X-axis) is based on the time when the pig went outside the ring (i.e. starting time of the visit). Different pigs are presented by a different color/box.

Table S1. Model output for the proportion of time outside the 50m ring.

A) Output of the full and final beta regression for the proportion of time outside the 50m ring, using the logit link function. Neighborhood, season and their two-way interaction were included in the model to account for the design (stratification per neighborhood and per season).

	**Full model**			**Final model**		
	**Coefficient**	**Standard error**	**p-value**	**Coefficient**	**Standard error**	**p-value**
Intercept	-1.22	0.53	0.022	-0.81	0.37	0.027
**Neighborhood**						
Neighborhood A	Ref.			Ref.		
Neighborhood B	0.60	0.50	0.228	0.41	0.49	0.405
**Season**						
Rainy	Ref.			Ref.		
Dry	0.47	0.50	0.347	0.25	0.49	0.615
**Interaction**						
Neighborhood B x dry season	-0.60	0.68	0.378	-0.33	0.66	0.623
**Sex**						
Female						
Male	0.68	0.44	0.121			
**Age group**						
Young (≤ 6 months)						
Old (> 6 months)	0.17	0.38	0.654			

B) Predicted proportion of time outside the 50m ring

**Neighborhood**	**Season**	**Predicted proportion [95% CI]**
A	Rainy	0.31 [0.18, 0.48]
B	Rainy	0.40 [0.26, 0.56]
A	Dry	0.36 [0.23, 0.52]
B	Dry	0.38 [0.25, 0.53]

Table S2. Model output for the proportion of time outside the 100m ring.

A) Output of the full and final beta regression for the proportion of time outside the 100m ring, using the logit link function. Neighborhood, season and their two-way interaction were included in the model to account for the design (stratification per neighborhood and per season). For two pigs that didn't go outside the 100m ring were given an arbitrary small value of 0.001.

	Full model			Final model		
	**Coefficient**	**Standard error**	**p-value**	**Coefficient**	**Standard error**	**p-value**
Intercept	-2.15	0.56	<0.001	-2.11	0.52	<0.001
**Neighborhood**						
Neighborhood A	Ref.			Ref.		
Neighborhood B	0.44	0.50	0.375	0.42	0.49	0.394
**Season**						
Rainy	Ref.			Ref.		
Dry	0.47	0.51	0.360	0.44	0.50	0.379
**Interaction**						
Neighborhood B x dry season	-0.40	0.69	0.559	-0.37	0.66	0.580
**Sex**						
Female	Ref.					
Male	0.08	0.44	0.856			
**Age group**						
Young (≤ 6 months)	Ref.			Ref.		
Old (> 6 months)	0.66	0.39	0.088	0.66	0.38	0.088

B) Predicted proportion of time outside the 100m ring

Neighborhood	Season	Age group	Predicted proportion of time [95% CI]
Neighborhood A	Rainy	Young	0.11 [0.04, 0.25]
Neighborhood B	Rainy	Young	0.16 [0.07, 0.32]
Neighborhood A	Dry	Young	0.16 [0.08, 0.30]
Neighborhood B	Dry	Young	0.17 [0.08, 0.30]
Neighborhood A	Rainy	Old	0.19 [0.10, 0.33]
Neighborhood B	Rainy	Old	0.26 [0.15, 0.41]
Neighborhood A	Dry	Old	0.27 [0.15, 0.43]
Neighborhood B	Dry	Old	0.28 [0.16, 0.44]

C) Estimated marginal effects of the proportion of time outside the 100m ring, at the margin of model terms neighborhood, season and pig age, .

Variable	Predicted proportion of time [95% CI]
Neighborhood A	0.20 [0.13, 0.29]
Neighborhood B	0.23 [0.16, 0.33]
Rainy season	0.20 [0.12, 0.29]
Dry season	0.24 [0.16, 0.34]
Young pigs	0.15 [0.08, 0.26]
Old pigs	0.25 [0.18, 0.34]

Table S3. Model output for going outside the 250m ring.

A) Output of the full and final logistic regression for going outside the 250m ring. Neighborhood, season and their two-way interaction were included in the model to account for the design (stratification per neighborhood and per season).

	**Full model**				**Final model**			
	**Odds ratio ($e^{ß}$)**	**95% CI**		**p-value**	**Odds ratio ($e^{ß}$)**	**95% CI**		**p-value**
Intercept	0.08	0.01	0.77	0.0440	0.29	0.04	1.18	0.1182
**Neighborhood**								
Neighborhood A	Ref.				Ref.			
Neighborhood B	5.03	0.70	51.42	0.1293	4.20	0.65	37.98	0.1532
**Season**								
Rainy	Ref.				Ref.			
Dry	26.72	2.88	438.19	0.0088	15.75	2.11	192.36	0.0138
**Interaction**								
Neighborhood B x dry season	0.09	0.00	1.51	0.1057	0.11	0.01	1.53	0.1119
**Sex**								
Female	Ref.							
Male	1.21	0.19	8.39	0.8407				
**Age group**								
Young (≤ 6 months)	Ref.							
Old (> 6 months)	3.57	0.72	21.89	0.1351				

B) Predicted probabilities of travelling more than 250m away, based on

**Neighborhood**	**Season**	**Predicted probability [95% CI]**
Neighborhood A	Rainy	0.22 [0.06, 0.58]
Neighborhood B	Rainy	0.55 [0.27, 0.80]
Neighborhood A	Dry	0.82 [0.49, 0.95]
Neighborhood B	Dry	0.67 [0.38, 0.87]

C) Estimated marginal effects of travelling more than 250m away, at the margin of model terms neighborhood and season.

**Variable**	**Predicted probability [95% CI]**
Neighborhood A	0.56 [0.29, 0.79]
Neighborhood B	0.61 [0.40, 0.79]
Rainy season	0.38 [0.19, 0.62]
Dry season	0.74 [0.53, 0.88]

Table S4. Model output for the maximum distance from home.

A) Output of the full and final generalized linear model, with gamma distribution and identity link function, for the maximum distance travelled from home. Neighborhood, season and their two-way interaction were included in the model to account for the design (stratification per neighborhood and per season).

	Full model			Final model		
	**Coefficient**	**Standard error**	**p-value**	**Coefficient**	**Standard error**	**p-value**
Intercept	159	52	0.004	152	49	0.003
**Neighborhood**						
Neighborhood A	Ref.			Ref.		
Neighborhood B	123	61	0.052	124	61	0.050
**Season**						
Rainy	Ref.			Ref.		
Dry	153	64	0.022	155	63	0.019
**Interaction**						
Neighborhood B x dry season	-208	88	0.024	-216	87	0.018
**Sex**						
Female	Ref.					
Male	-26	48	0.592			
**Age group**						
Young (≤ 6 months)	Ref.			Ref.		
Old (> 6 months)	91	44	0.047	95	44	0.036

B) Predicted maximum distance (in m)

Neighborhood	Season	Age group	Predicted maximum distance [95% CI]
Neighborhood A	Rainy	Young	152 [53, 251]
Neighborhood B	Rainy	Young	276 [157, 394]
Neighborhood A	Dry	Young	307 [194, 420]
Neighborhood B	Dry	Young	215 [135, 295]
Neighborhood A	Rainy	Old	248 [172, 324]
Neighborhood B	Rainy	Old	371 [268, 475]
Neighborhood A	Dry	Old	402 [290, 515]
Neighborhood B	Dry	Old	311 [222, 399]

C) Estimated maximum distance, at the margin of model terms neighborhood, season and pig age, based on the final model specified above.

Variable	Predicted maximum distance [95% CI]
Neighborhood A	304 [238, 369]
Neighborhood B	312 [250, 374]
Rainy season	287 [224, 350]
Dry season	327 [261, 393]
Young pigs	239 [172, 307]
Old pigs	335 [277, 393]
