# Supplementary material for: Movements of free-range pigs in rural communities in Zambia: an explorative study towards future ring interventions for the control of Taenia solium
Source: Parasit Vectors. 2022 Apr 27;15:150. doi: 10.1186/s13071-022-05264-0 (PMC9044682; doi:10.1186/s13071-022-05264-0)

Additional file 2: GPS tracks and summary data of individual pigs

This document contains the GPS tracks and summary data of the 43 pigs used in the final analysis set. The summary table shows the residence time (in hours) within circles of different radii around the pig owner's household. Also the proportion of the total tracking time (in %) and the number of different visits outside the ring are shown.

The histogram shows the number of data points at different distances away from the pig owner's household.

The househould coordinate of the pig's owner is indicated with a green dot. The tracks are shown using a different color per day. The rings are presented as circles of different radii around the pig owner's household (50-m ring with a solid line, 100-m ring with a dashed line and 250-m ring with a dotted line).

Pig 1

Neighborhood	Neighborhood A
Season	October
Sex	F
Age (months)	14

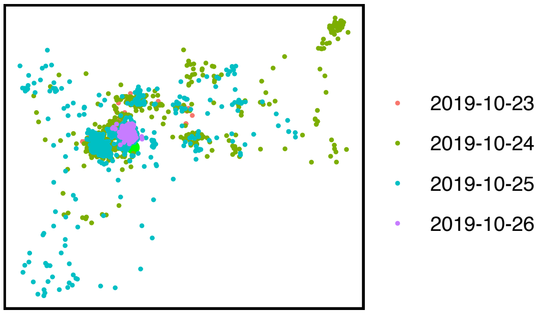

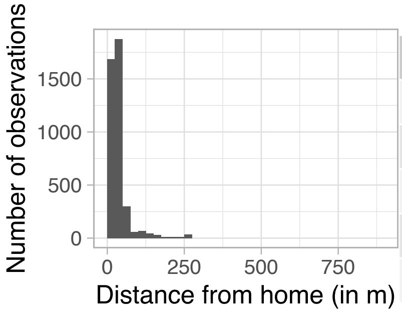

Radius	Residence time	Proportion of total time	Number of visits
50m	61.9 h	14%	48
100m	68.5 h	4.9%	15
150m	70.3 h	2.3%	6
200m	71 h	1.4%	2
250m	71.4 h	0.8%	1

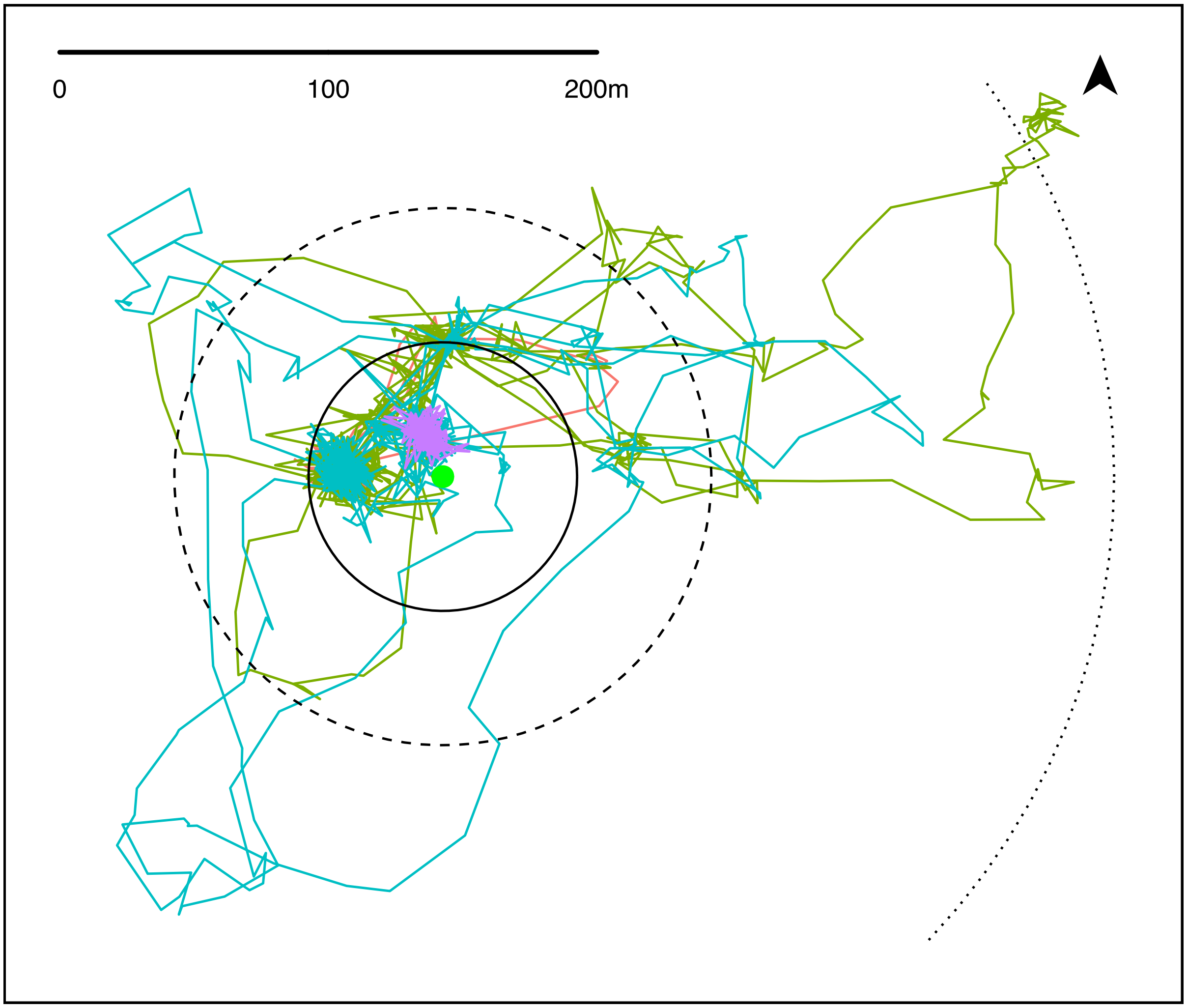

Pig 2

Neighborhood	Neighborhood A
Season	October
Sex	F
Age (months)	10

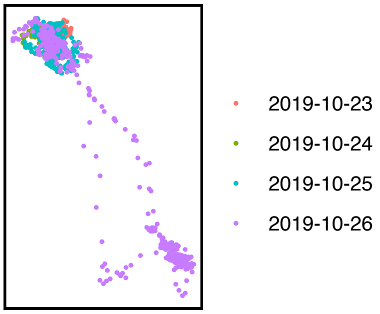

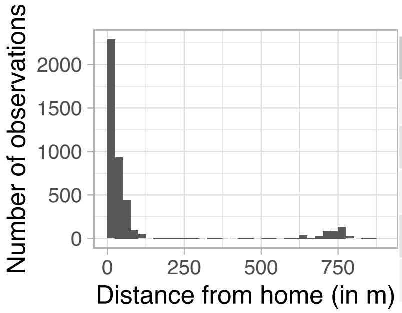

Radius	Residence time	Proportion of total time	Number of visits
50m	55.1 h	23.5%	76
100m	63.9 h	11.2%	11
150m	64.7 h	10.1%	2
200m	64.8 h	10%	2
250m	64.9 h	9.9%	2

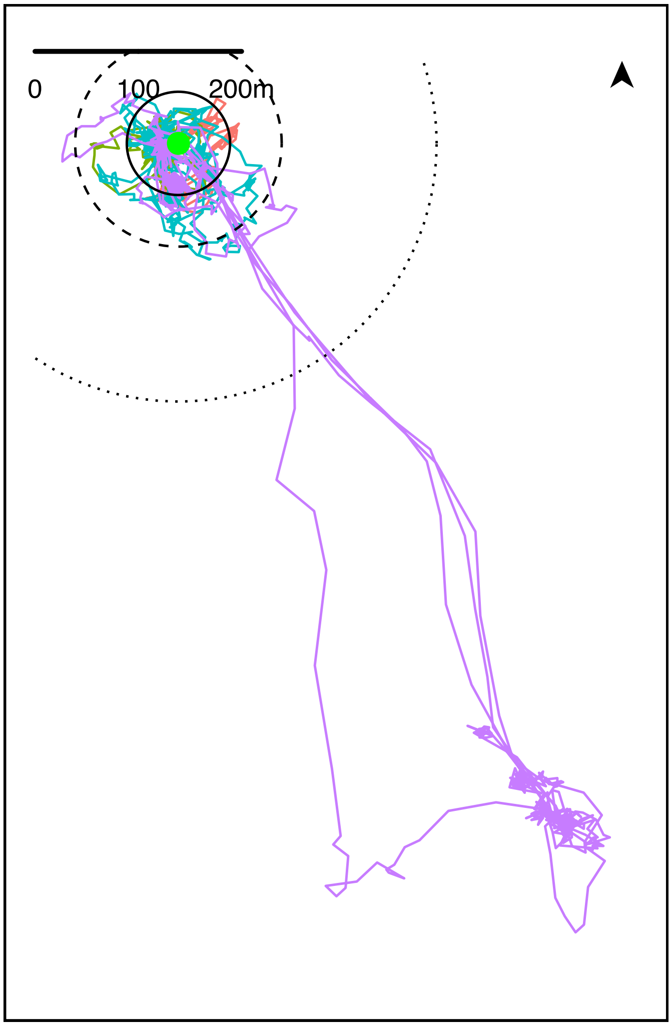

Pig 3

Neighborhood	Neighborhood A
Season	October
Sex	F
Age (months)	8

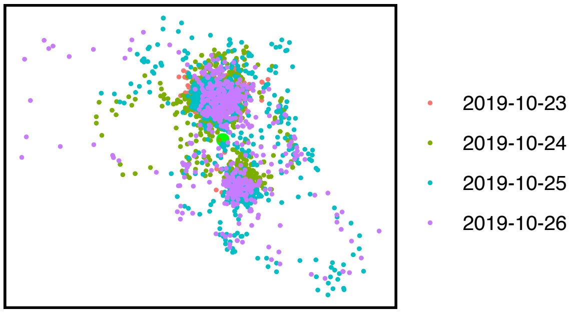

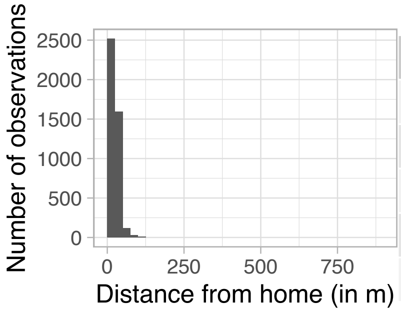

Radius	Residence time	Proportion of total time	Number of visits
50m	67.9 h	3.5%	18
100m	70.2 h	0.2%	3
150m	70.4 h	0%	0
200m	70.4 h	0%	0
250m	70.4 h	0%	0

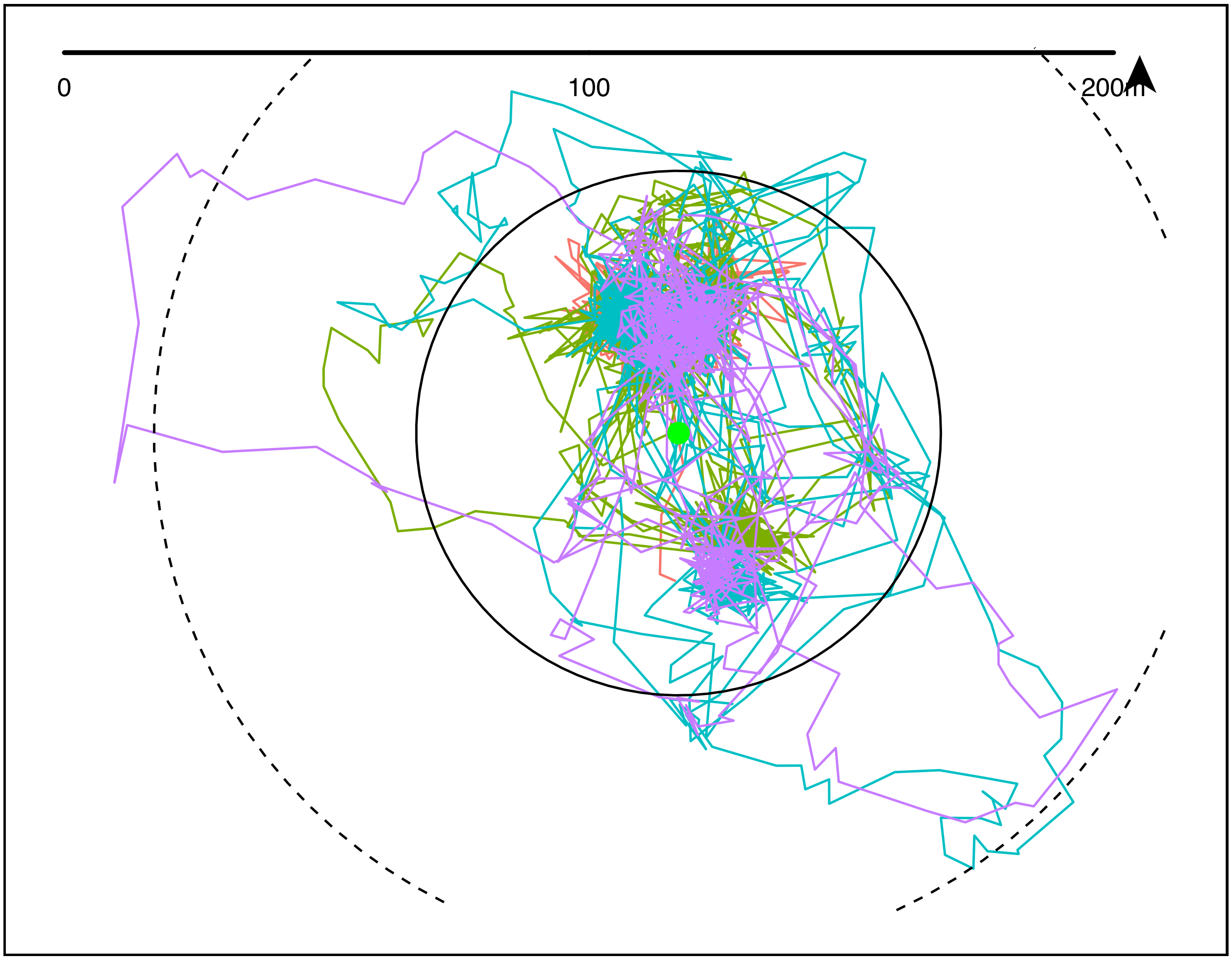

Pig 4

Neighborhood	Neighborhood A
Season	October
Sex	F
Age (months)	6

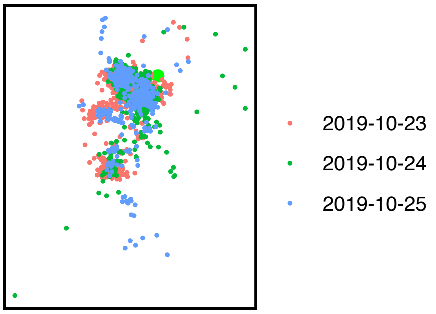

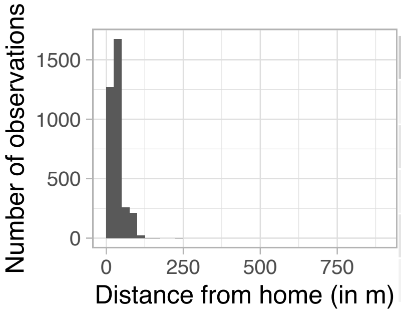

Radius	Residence time	Proportion of total time	Number of visits
50m	55.3 h	14.9%	32
100m	64.3 h	1%	9
150m	64.8 h	0.2%	3
200m	64.9 h	0.1%	1
250m	64.9 h	0%	0

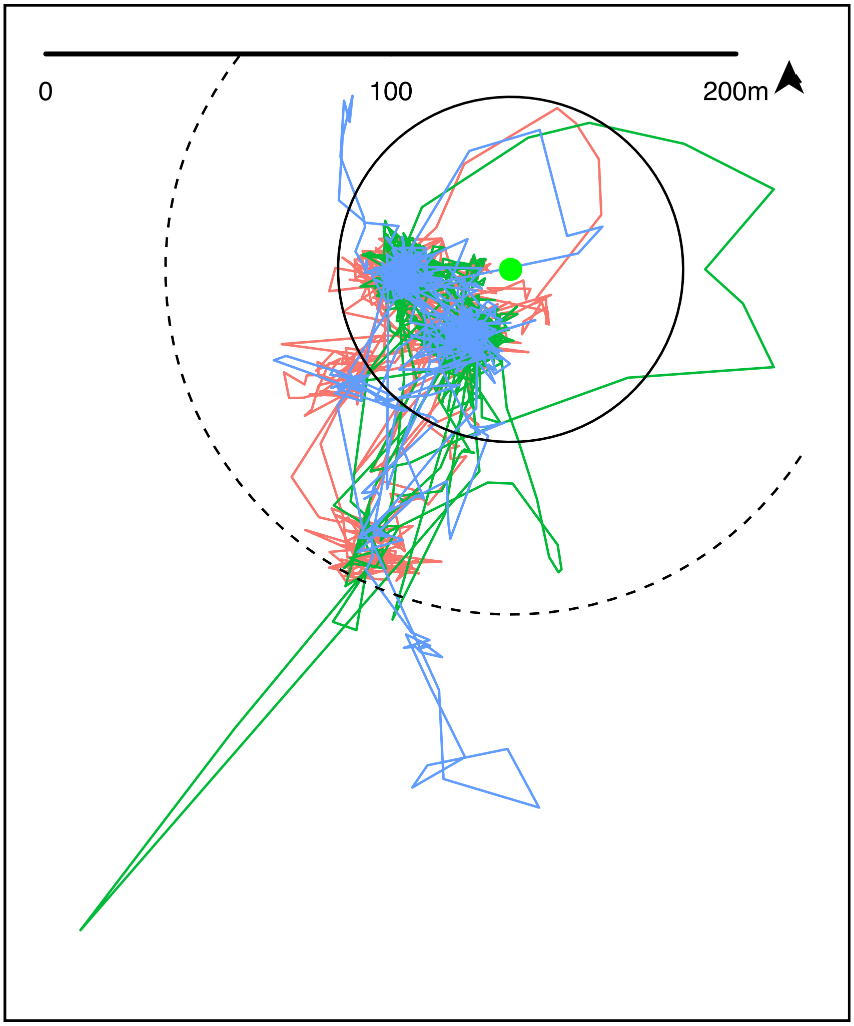

Pig 5

Neighborhood	Neighborhood A
Season	October
Sex	M
Age (months)	5

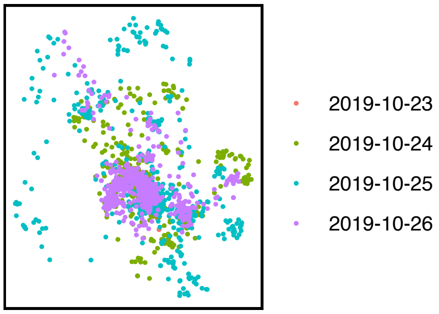

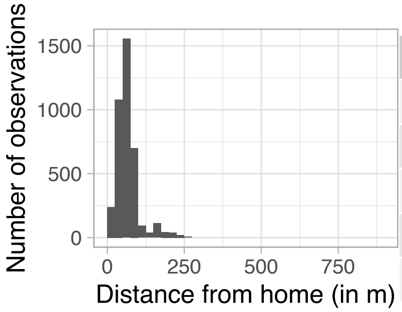

Radius	Residence time	Proportion of total time	Number of visits
50m	24 h	64.7%	185
100m	62.1 h	8.6%	26
150m	64.2 h	5.5%	11
200m	66.8 h	1.6%	3
250m	67.8 h	0.2%	3

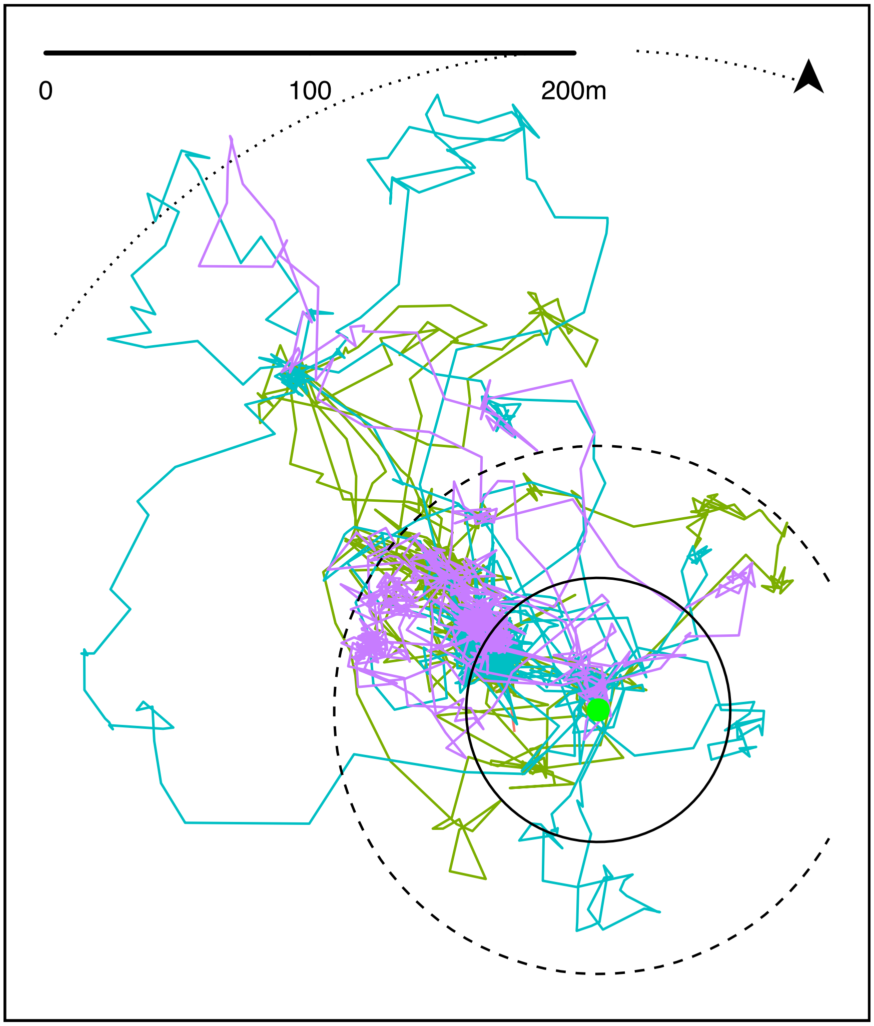

Pig 6

Neighborhood	Neighborhood A
Season	October
Sex	F
Age (months)	21

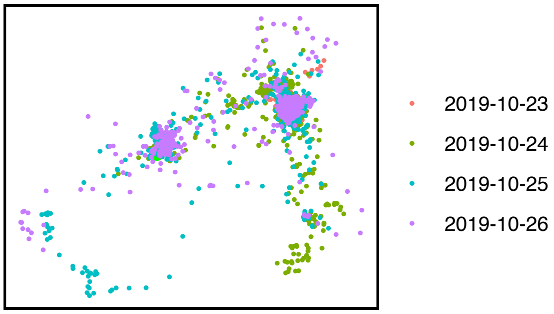

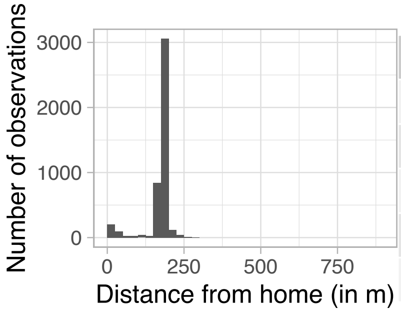

Radius	Residence time	Proportion of total time	Number of visits
50m	5.1 h	93.3%	20
100m	6 h	92.1%	18
150m	7.1 h	90.6%	17
200m	72.6 h	3.6%	27
250m	75.1 h	0.2%	3

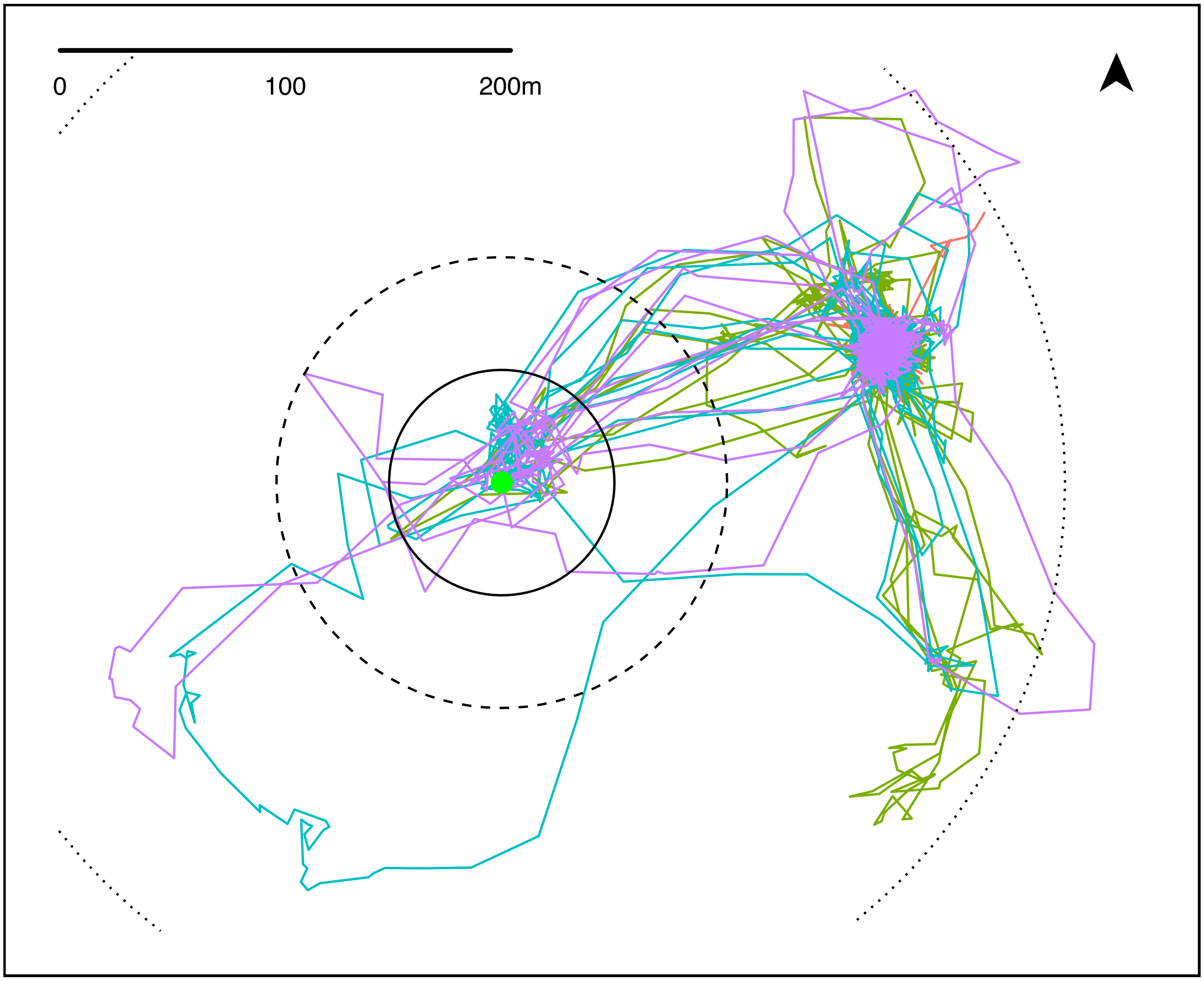

Pig 7

Neighborhood	Neighborhood A
Season	October
Sex	F
Age (months)	6

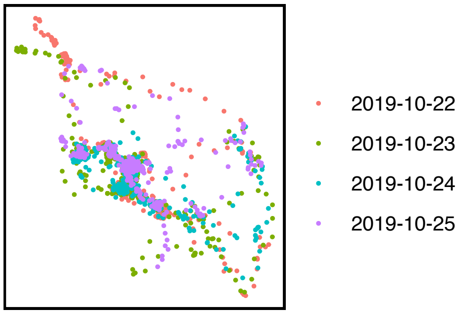

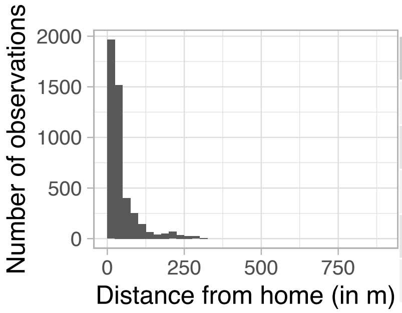

Radius	Residence time	Proportion of total time	Number of visits
50m	57.2 h	24.4%	40
100m	68.1 h	9.9%	37
150m	71.5 h	5.5%	13
200m	73.1 h	3.4%	21
250m	74.8 h	1.2%	6

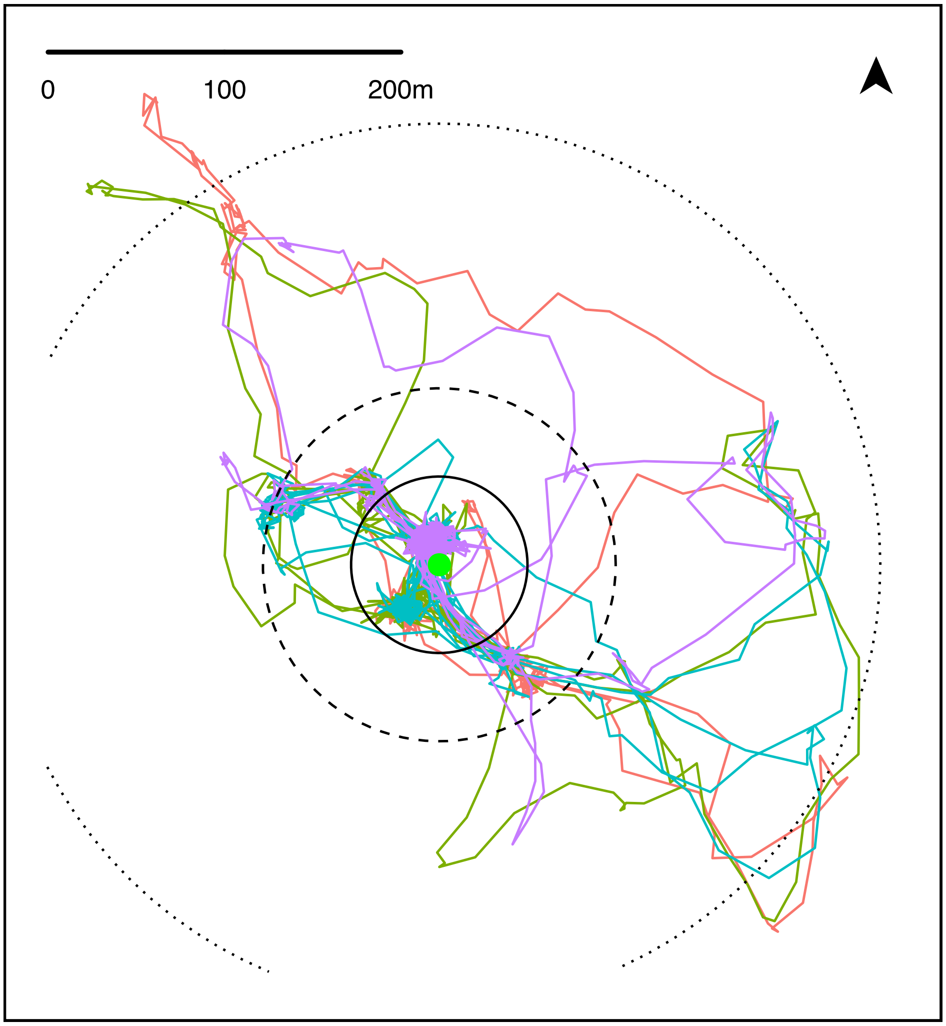

Pig 8

Neighborhood	Neighborhood A
Season	October
Sex	F
Age (months)	14

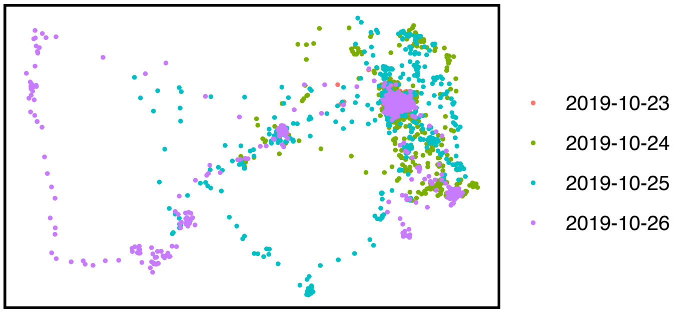

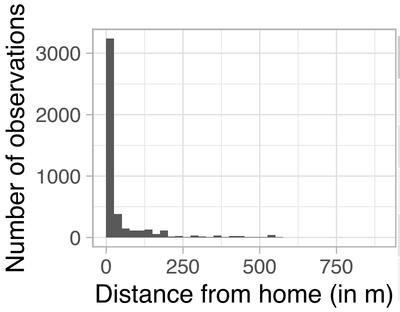

Radius	Residence time	Proportion of total time	Number of visits
50m	62.9 h	18.4%	32
100m	67.1 h	13%	27
150m	71 h	7.9%	19
200m	73.6 h	4.6%	6
250m	74.1 h	3.9%	4

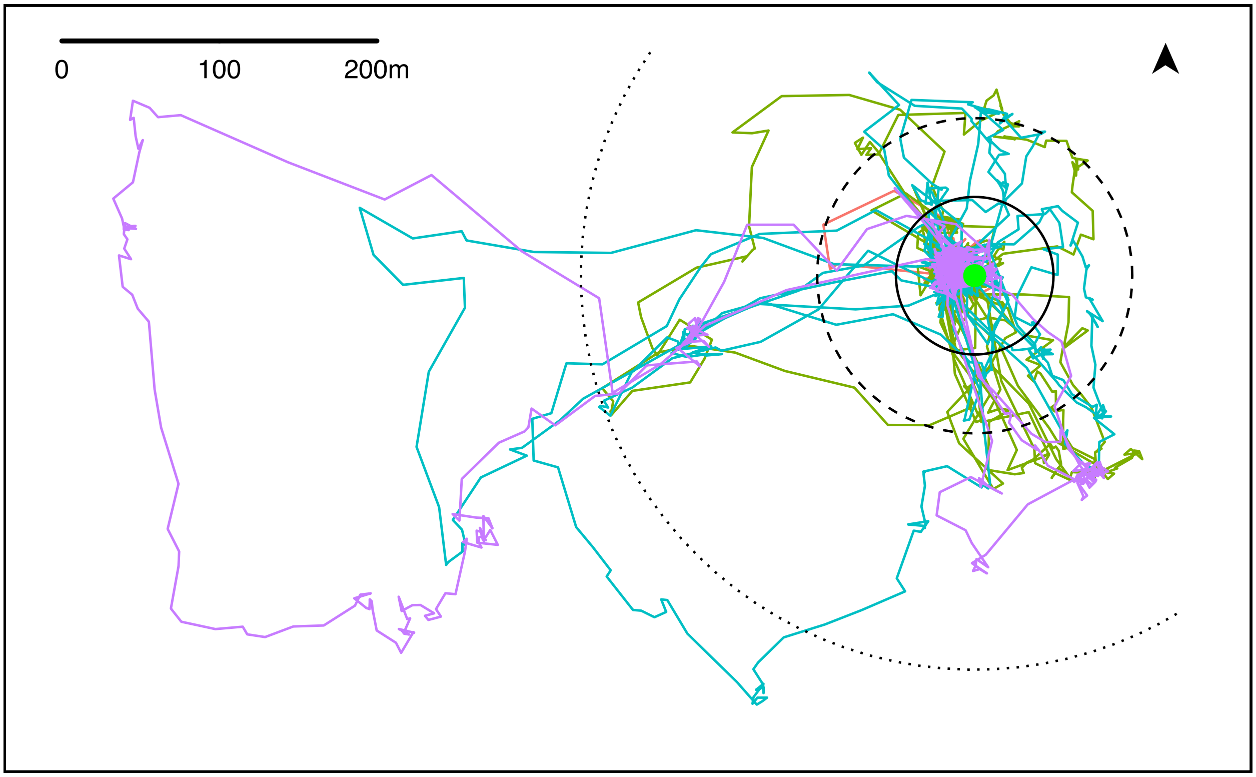

Pig 9

Neighborhood	Neighborhood A
Season	October
Sex	F
Age (months)	11

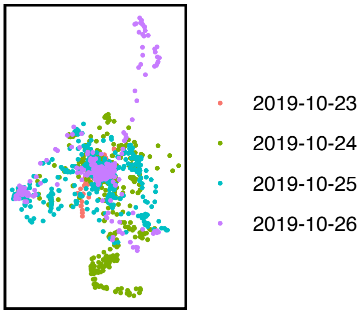

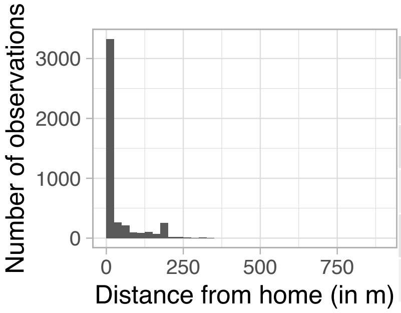

Radius	Residence time	Proportion of total time	Number of visits
50m	61.3 h	19.7%	38
100m	66.4 h	13.1%	24
150m	69.7 h	8.8%	18
200m	75.2 h	1.7%	6
250m	75.8 h	0.7%	3

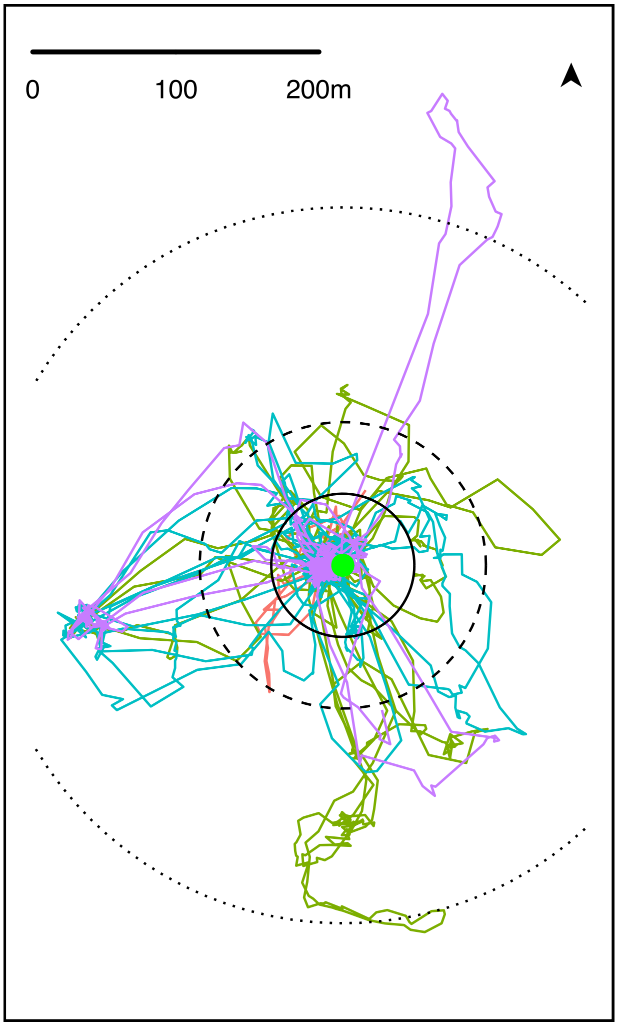

Pig 10

Neighborhood	Neighborhood A
Season	October
Sex	F
Age (months)	22

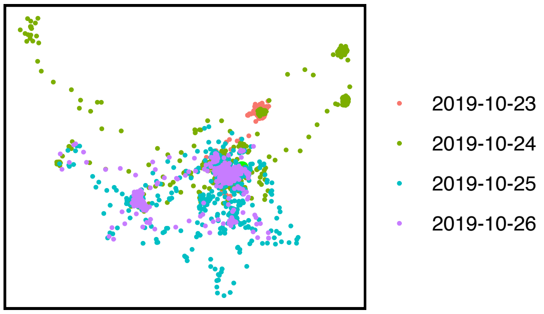

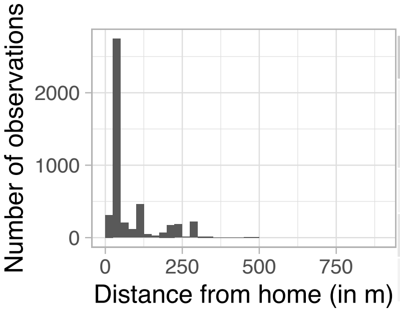

Radius	Residence time	Proportion of total time	Number of visits
50m	49.8 h	33.7%	62
100m	54.8 h	27%	29
150m	63.1 h	16%	13
200m	64.8 h	13.8%	37
250m	70.5 h	6.1%	6

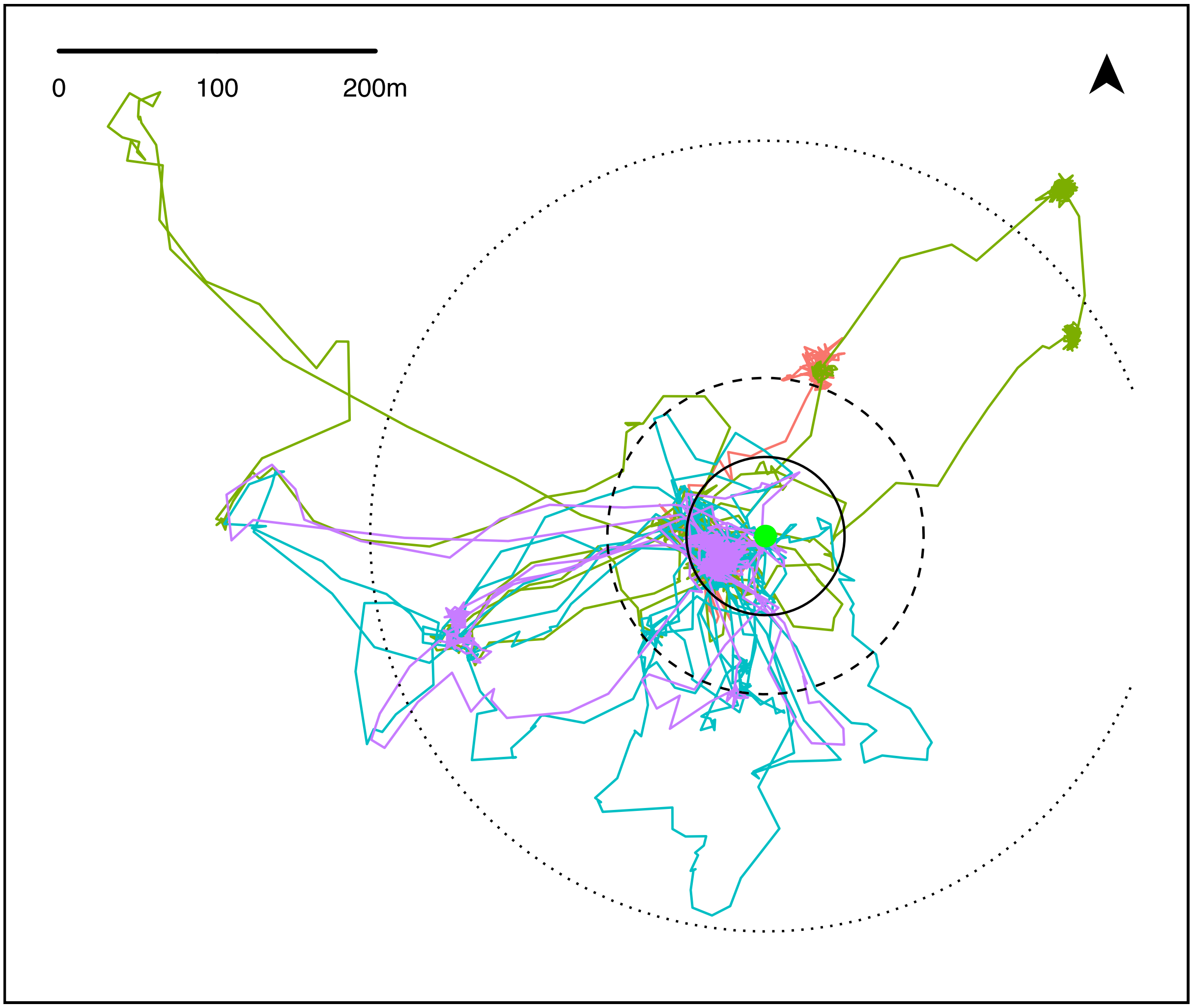

Pig 11

Neighborhood	Neighborhood A
Season	October
Sex	F
Age (months)	5

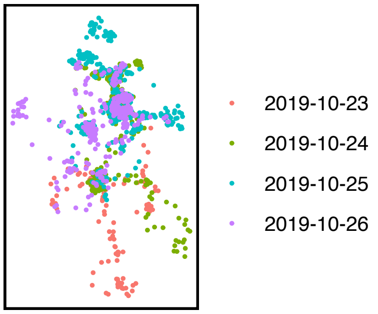

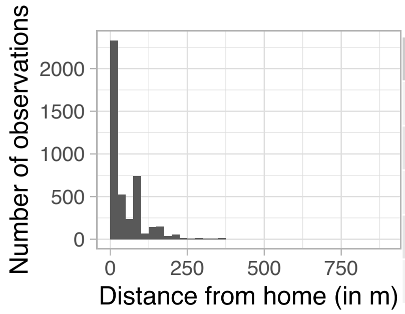

Radius	Residence time	Proportion of total time	Number of visits
50m	46.9 h	35%	35
100m	63.7 h	11.8%	22
150m	67.1 h	7.1%	20
200m	70.2 h	2.8%	7
250m	71.3 h	1.2%	3

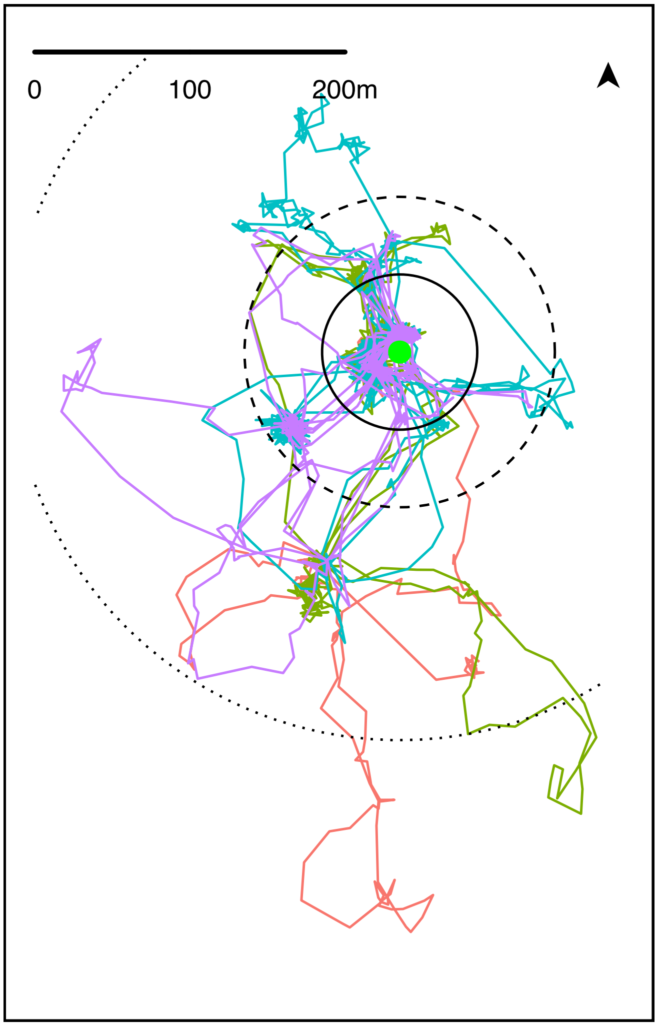

Pig 12

Neighborhood	Neighborhood B
Season	October
Sex	F
Age (months)	8

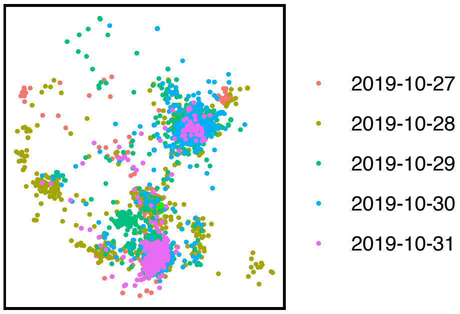

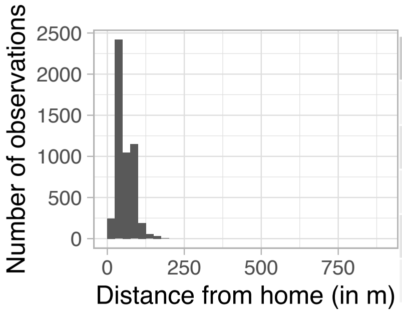

Radius	Residence time	Proportion of total time	Number of visits
50m	44.6 h	50.4%	237
100m	84.8 h	5.7%	57
150m	89.3 h	0.7%	6
200m	89.9 h	0%	0
250m	89.9 h	0%	0

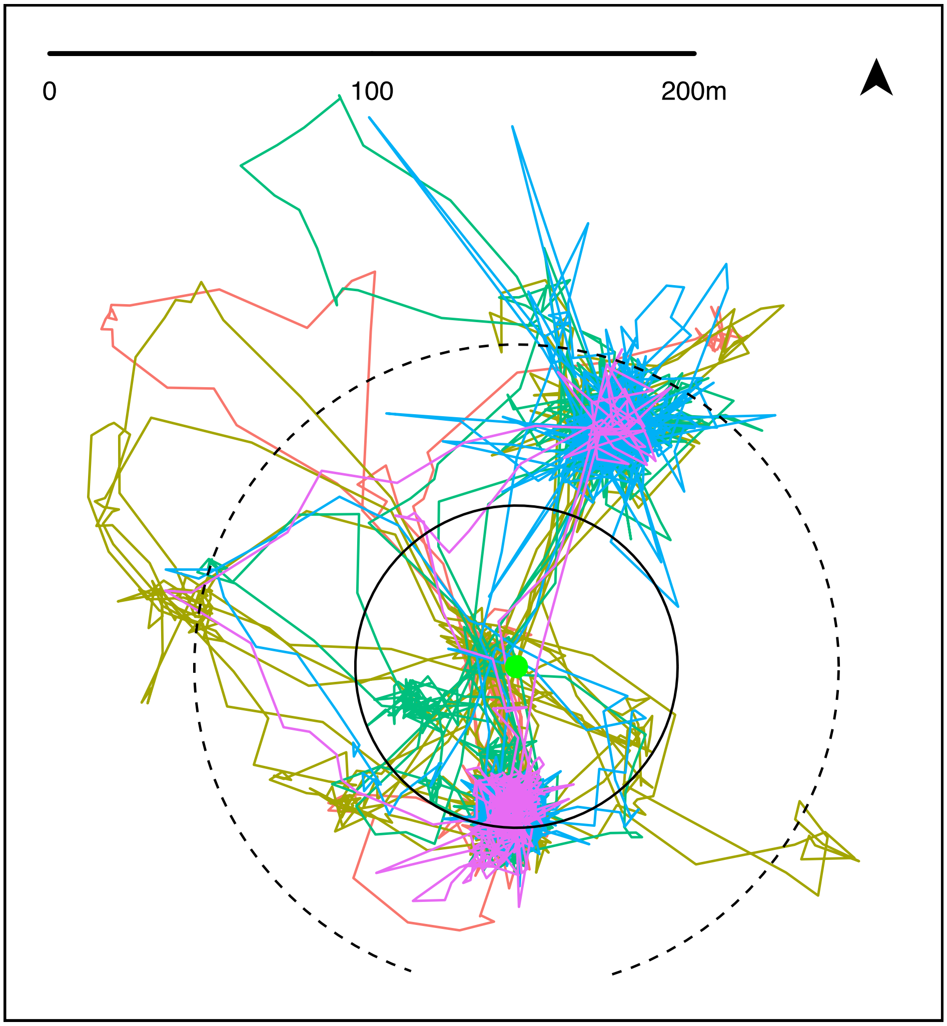

Pig 13

Neighborhood	Neighborhood B
Season	October
Sex	M
Age (months)	12

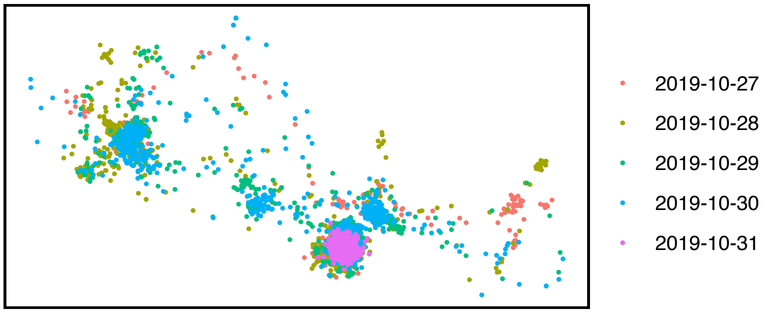

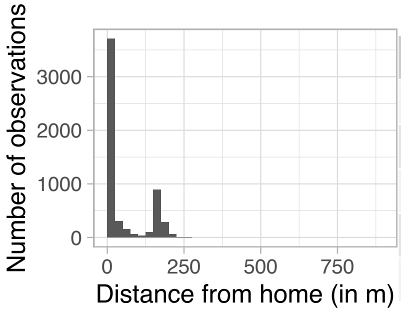

Radius	Residence time	Proportion of total time	Number of visits
50m	67.5 h	28.7%	30
100m	71 h	24.9%	13
150m	73.2 h	22.6%	23
200m	93.5 h	1.1%	8
250m	94.6 h	0%	1

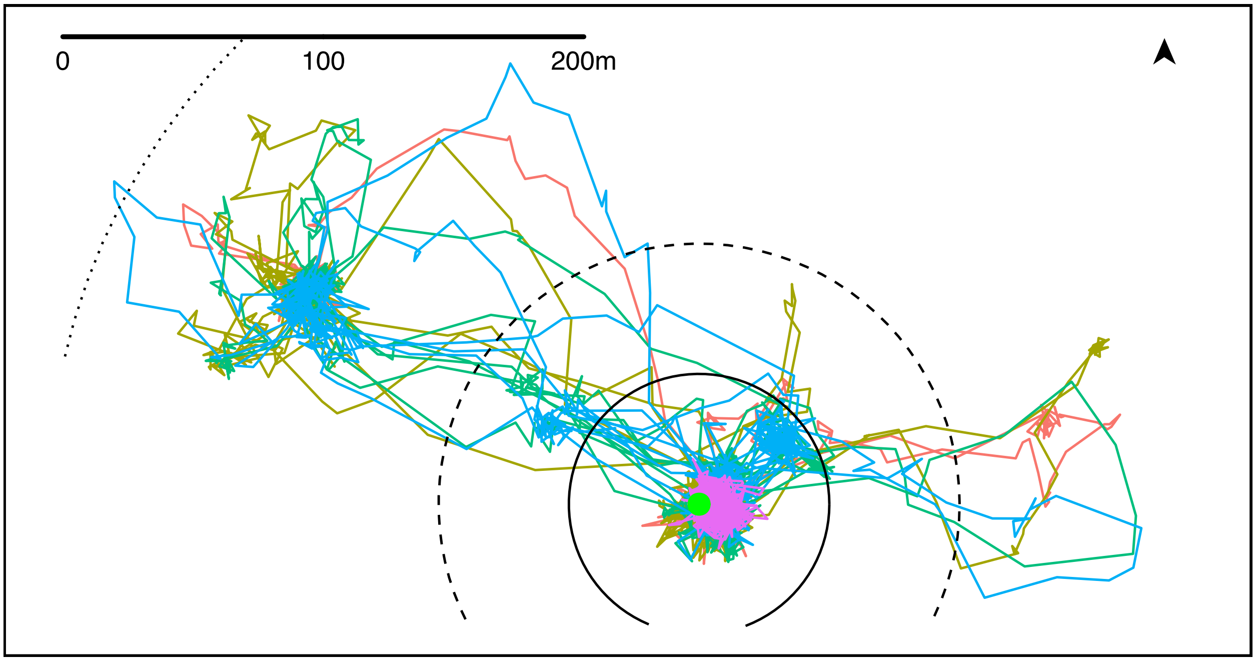

Pig 14

Neighborhood	Neighborhood B
Season	October
Sex	F
Age (months)	6

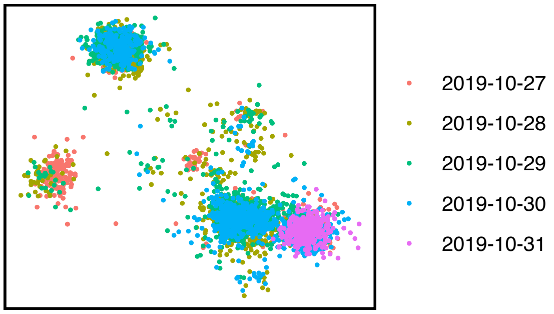

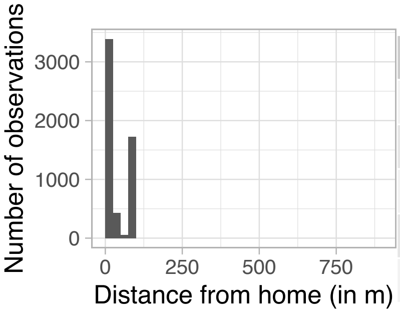

Radius	Residence time	Proportion of total time	Number of visits
50m	64.8 h	31.2%	10
100m	94.2 h	0%	0
150m	94.2 h	0%	0
200m	94.2 h	0%	0
250m	94.2 h	0%	0

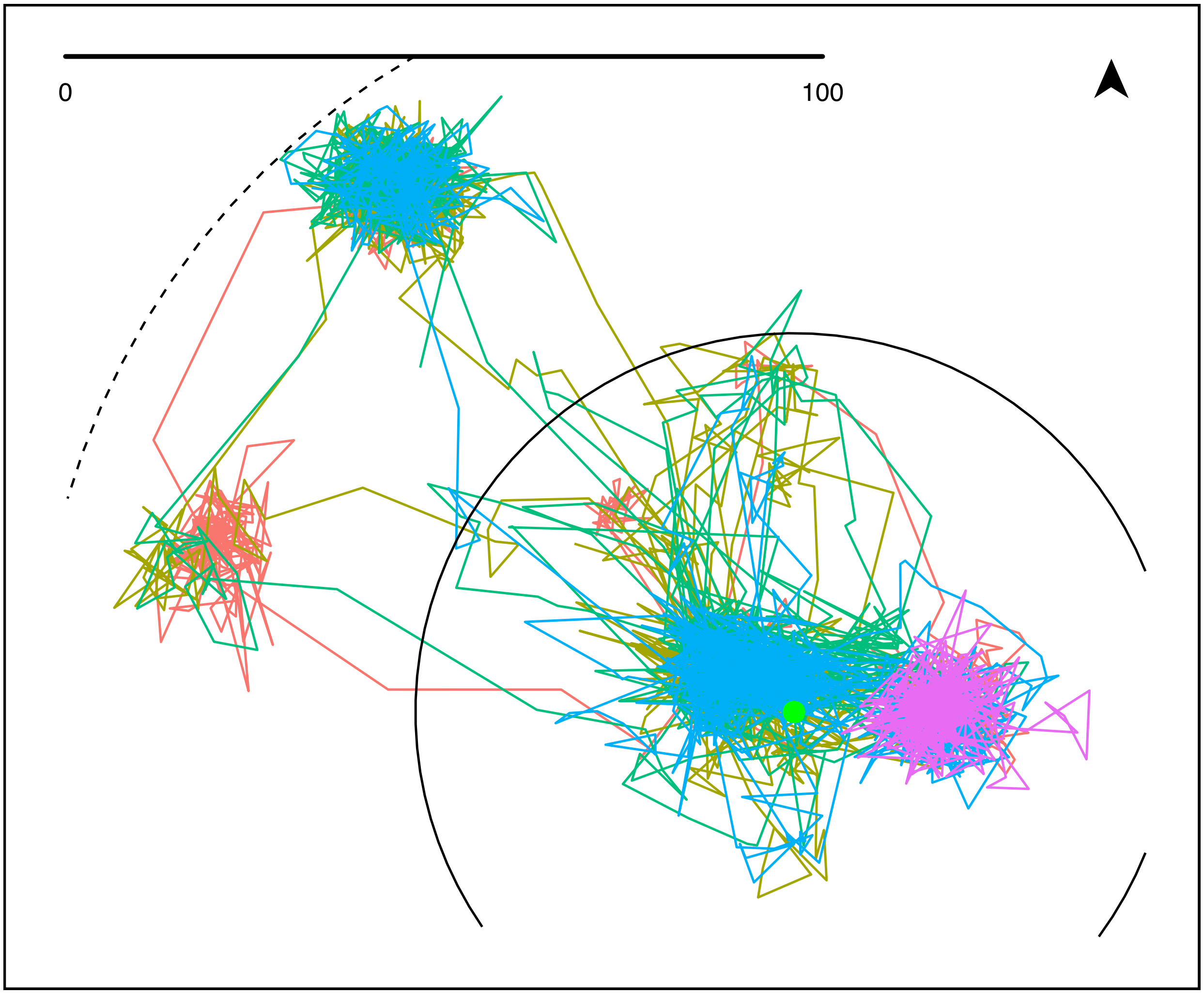

Pig 15

Neighborhood	Neighborhood B
Season	October
Sex	F
Age (months)	6

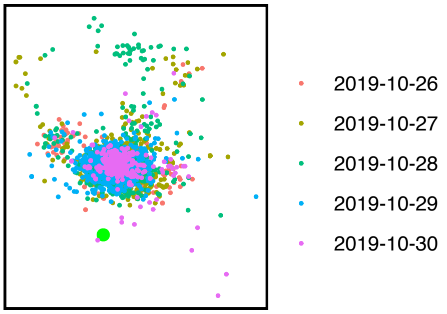

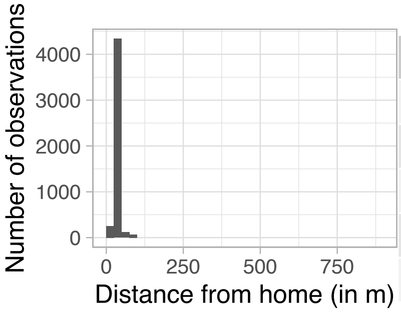

Radius	Residence time	Proportion of total time	Number of visits
50m	87.4 h	4%	55
100m	91.1 h	0%	0
150m	91.1 h	0%	0
200m	91.1 h	0%	0
250m	91.1 h	0%	0

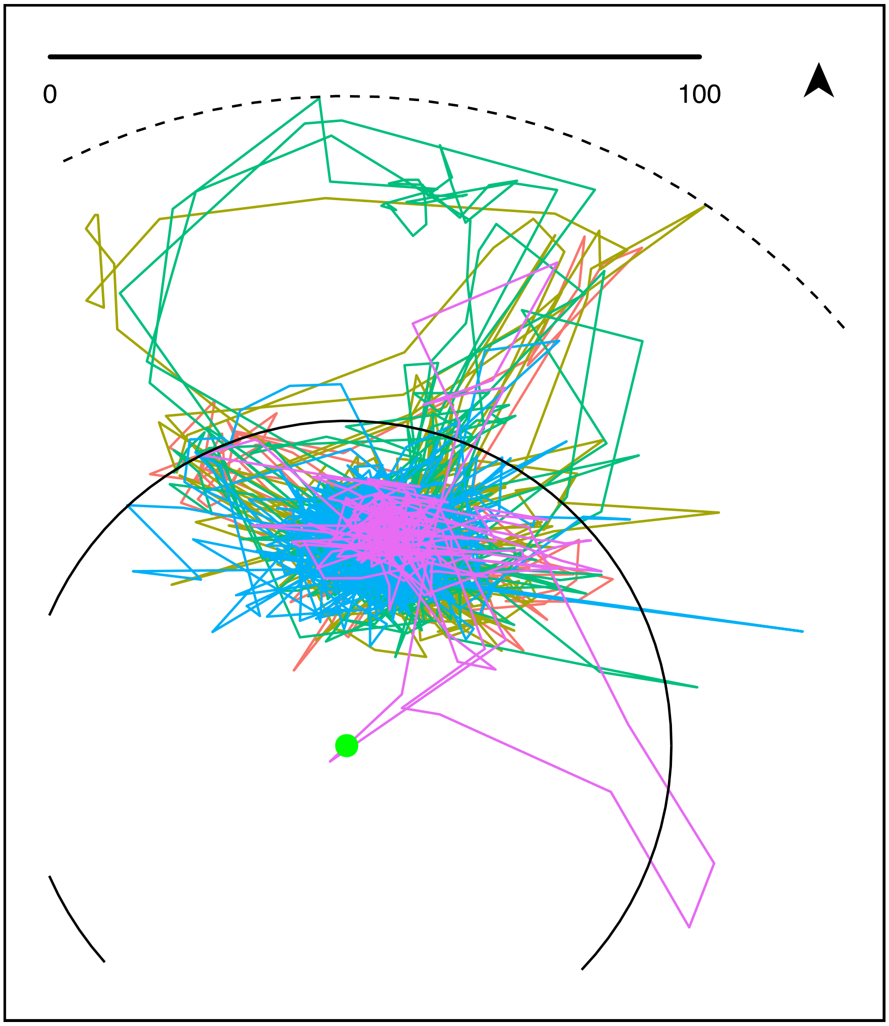

Pig 16

Neighborhood	Neighborhood B
Season	October
Sex	F
Age (months)	55

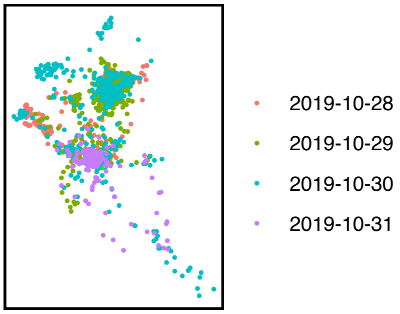

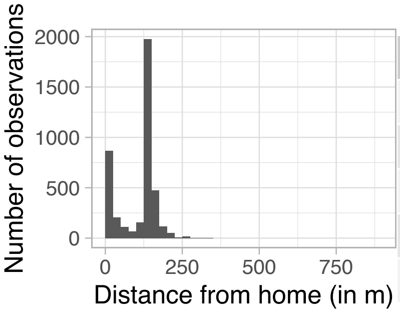

Radius	Residence time	Proportion of total time	Number of visits
50m	18 h	73.7%	26
100m	20.9 h	69.5%	19
150m	57.2 h	16.5%	60
200m	67.2 h	1.9%	10
250m	68.1 h	0.5%	3

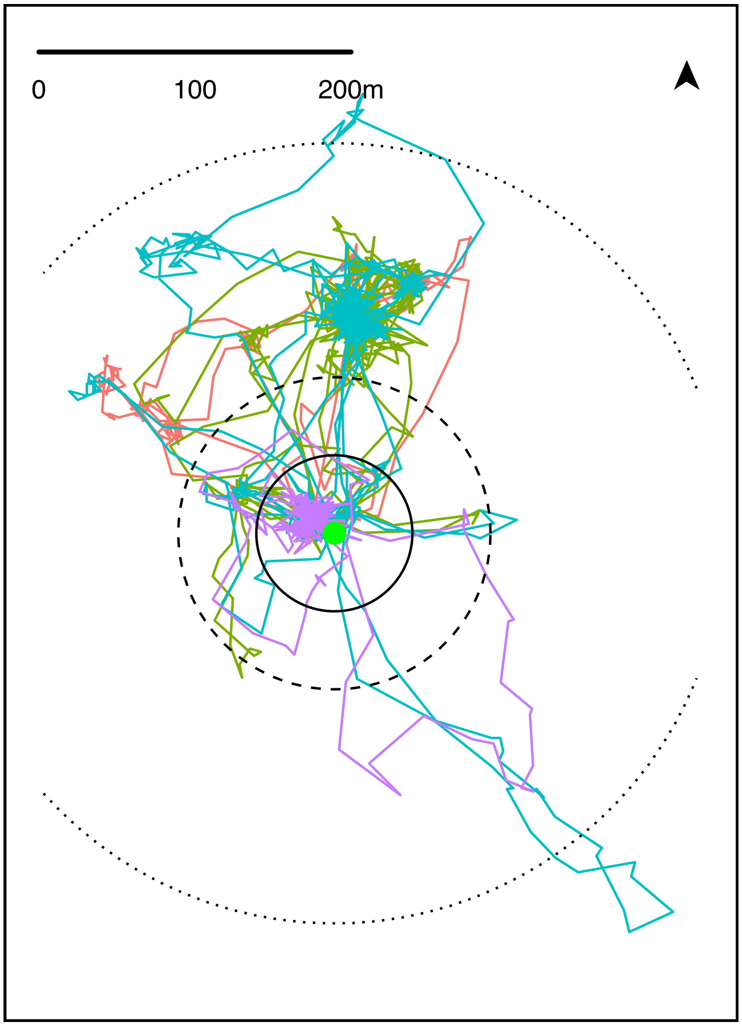

Pig 17

Neighborhood	Neighborhood B
Season	October
Sex	F
Age (months)	16

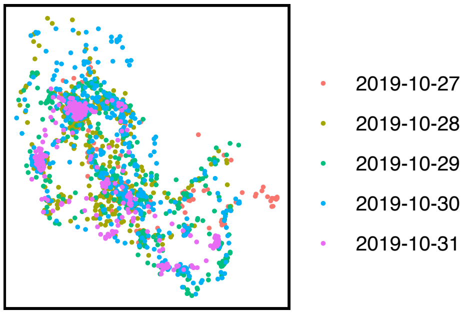

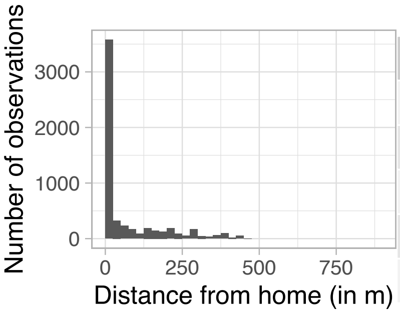

Radius	Residence time	Proportion of total time	Number of visits
50m	65.6 h	31.2%	54
100m	72.2 h	24.2%	34
150m	77 h	19.2%	43
200m	81.5 h	14.4%	42
250m	86.2 h	9.5%	15

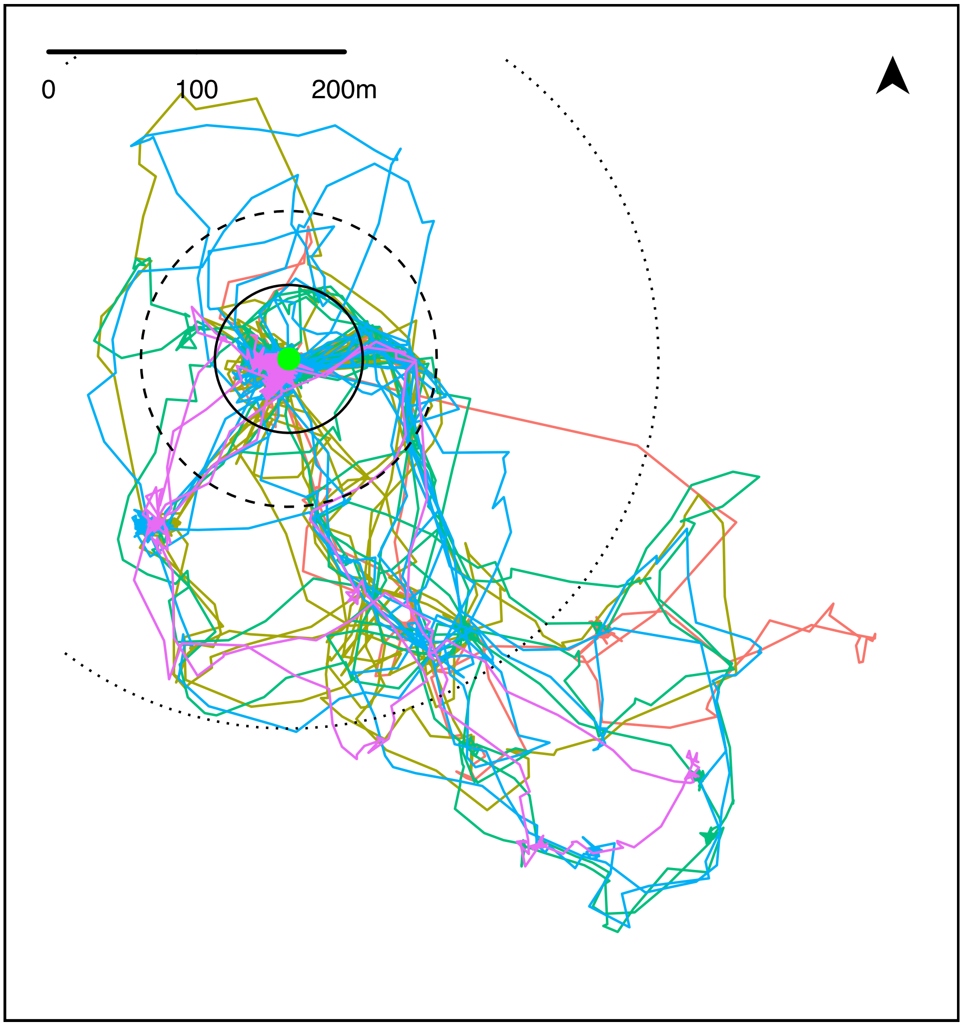

Pig 18

Neighborhood	Neighborhood B
Season	October
Sex	F
Age (months)	5

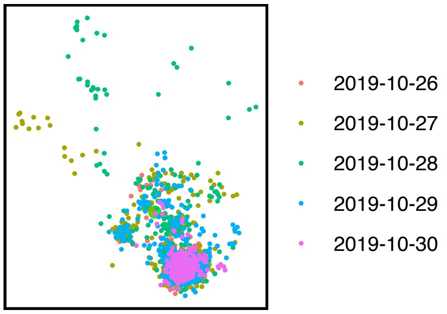

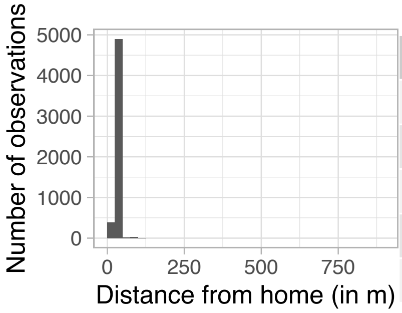

Radius	Residence time	Proportion of total time	Number of visits
50m	89.5 h	0.9%	5
100m	90.2 h	0.2%	1
150m	90.3 h	0%	0
200m	90.3 h	0%	0
250m	90.3 h	0%	0

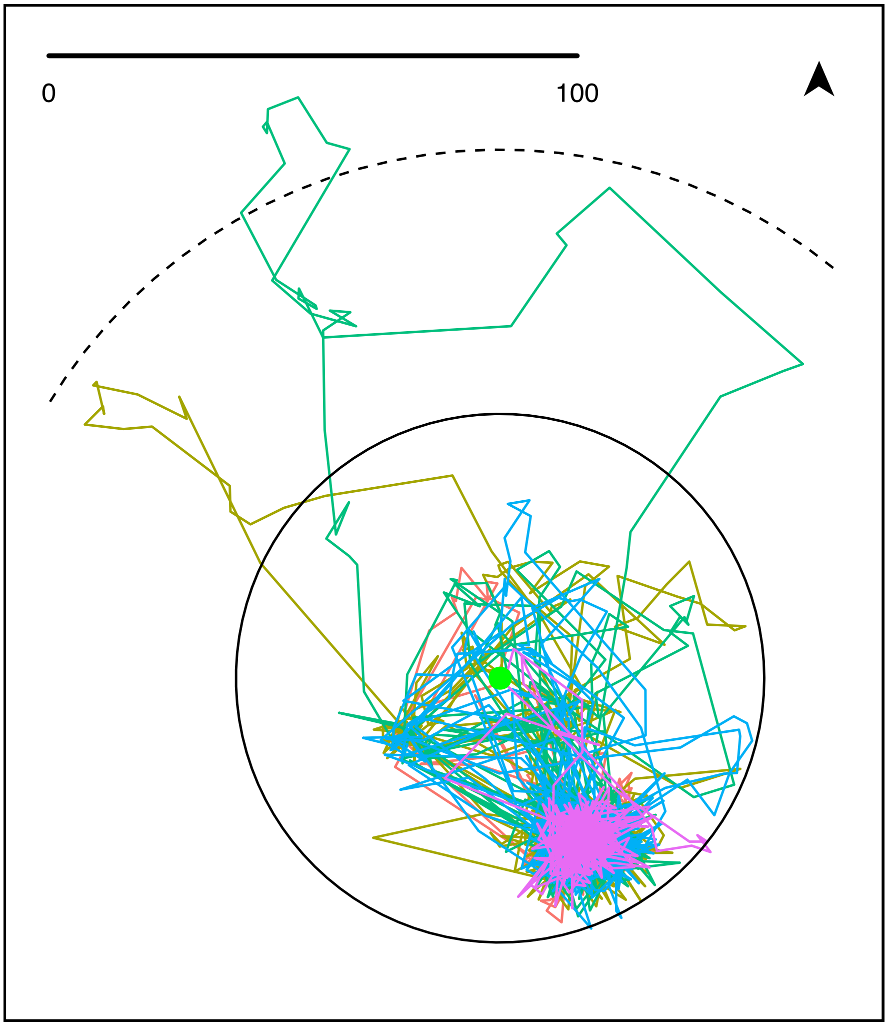

Pig 19

Neighborhood	Neighborhood B
Season	October
Sex	M
Age (months)	5

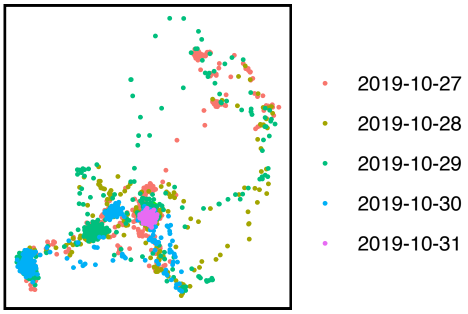

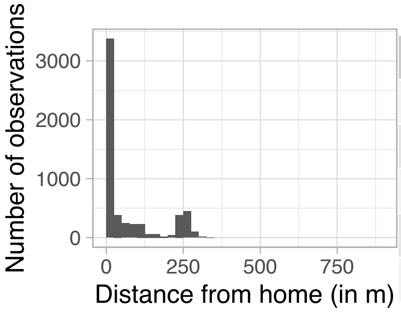

Radius	Residence time	Proportion of total time	Number of visits
50m	63.7 h	33%	24
100m	71.6 h	24.5%	39
150m	76.7 h	19.2%	20
200m	78 h	17.8%	13
250m	85.4 h	10.1%	107

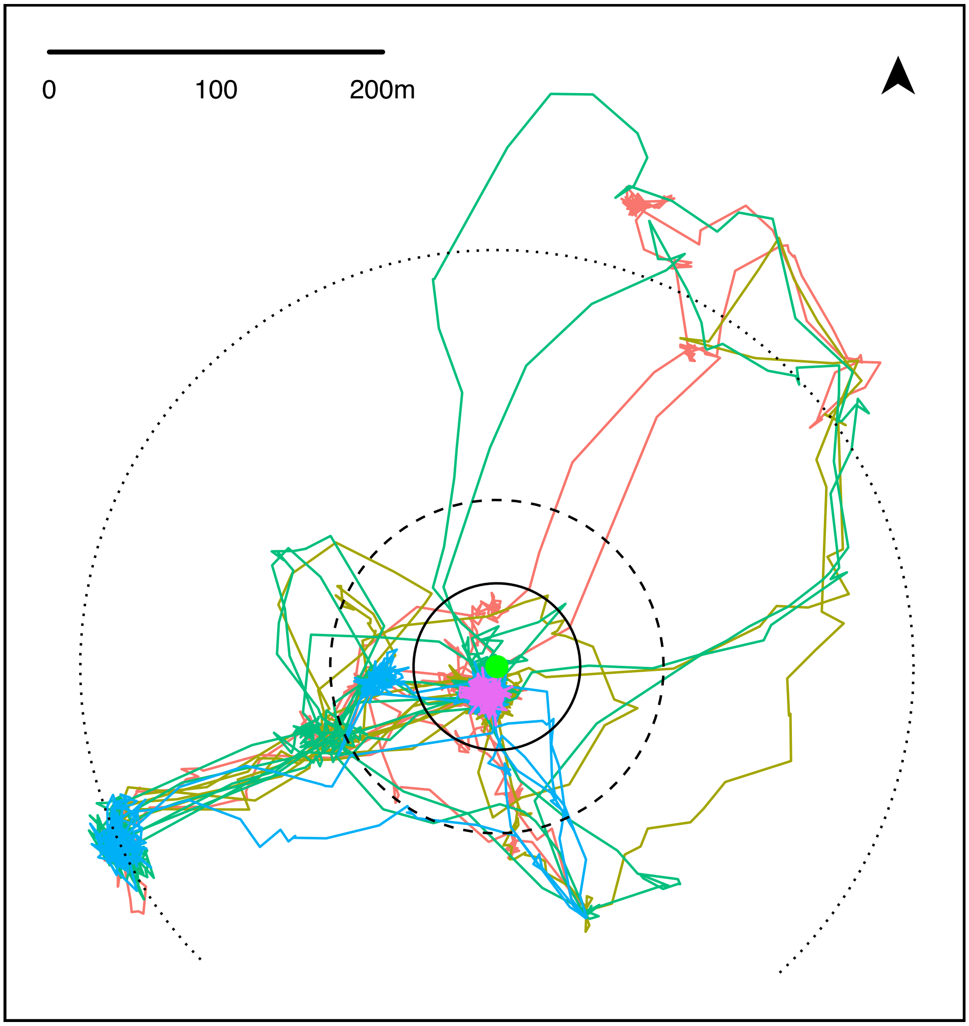

Pig 20

Neighborhood	Neighborhood B
Season	October
Sex	F
Age (months)	71

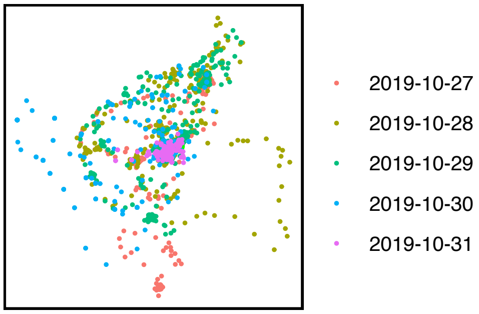

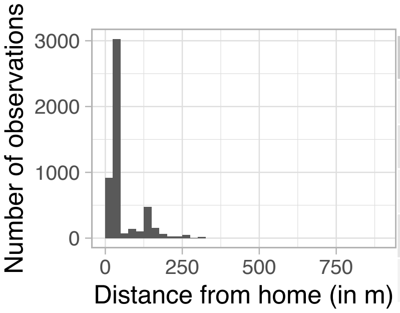

Radius	Residence time	Proportion of total time	Number of visits
50m	74.1 h	20.8%	27
100m	77.7 h	16.9%	24
150m	87.7 h	6.2%	25
200m	91.4 h	2.2%	10
250m	92.4 h	1.2%	5

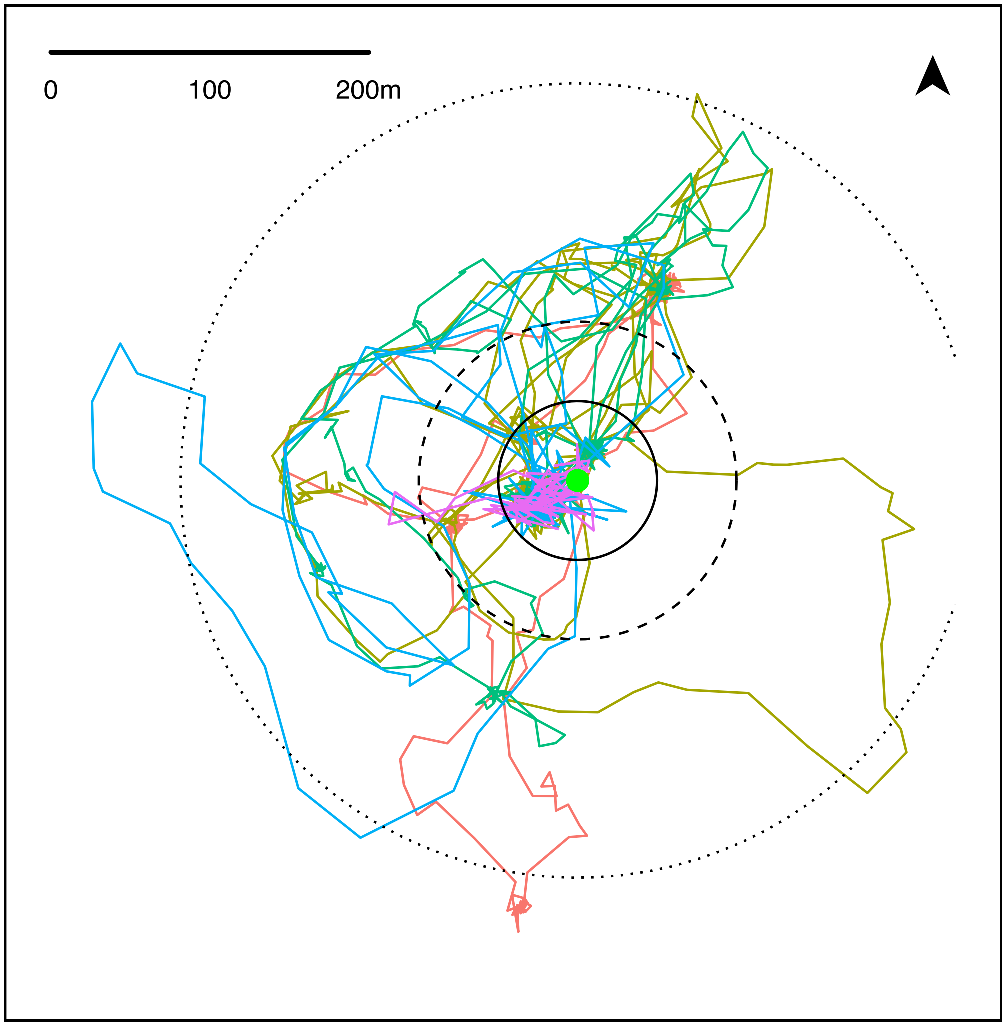

Pig 21

Neighborhood	Neighborhood B
Season	October
Sex	F
Age (months)	45

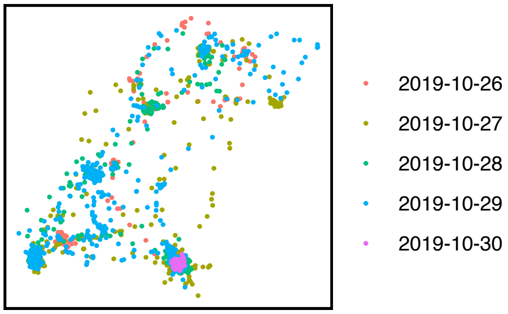

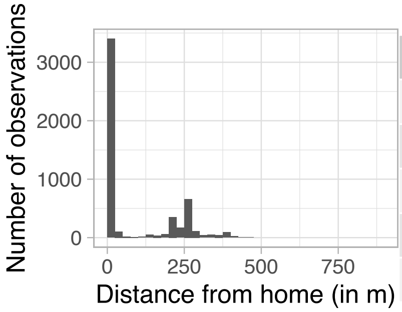

Radius	Residence time	Proportion of total time	Number of visits
50m	61.5 h	33.7%	11
100m	62.7 h	32.5%	6
150m	63.7 h	31.4%	12
200m	65.3 h	29.6%	24
250m	75 h	19.2%	44

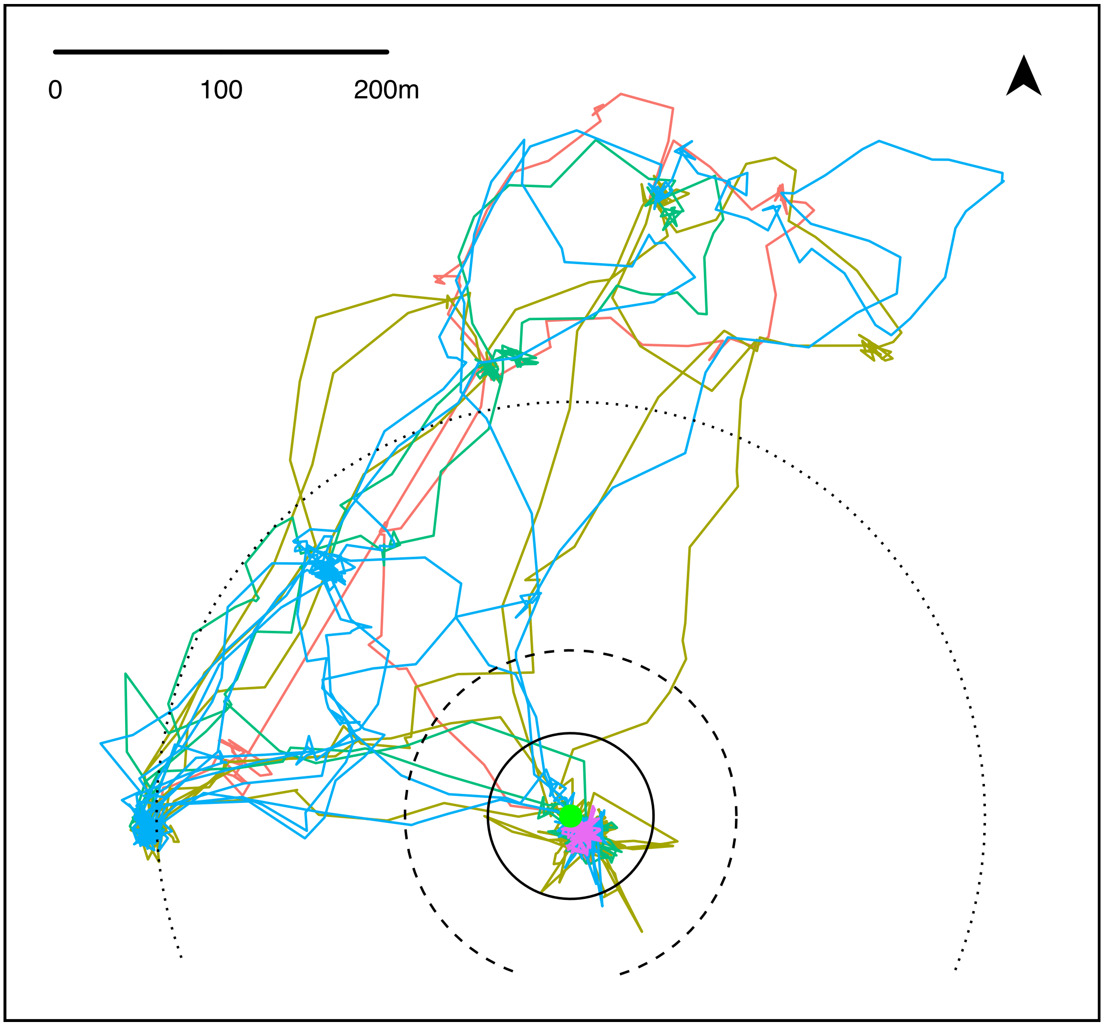

Pig 22

Neighborhood	Neighborhood B
Season	October
Sex	F
Age (months)	4

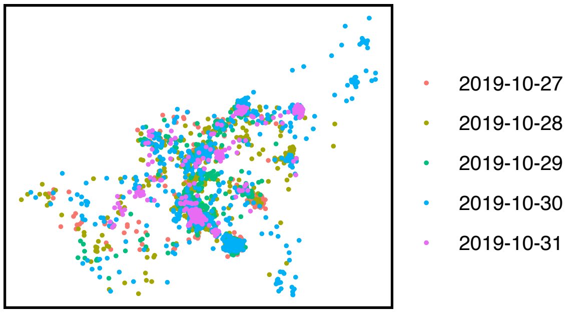

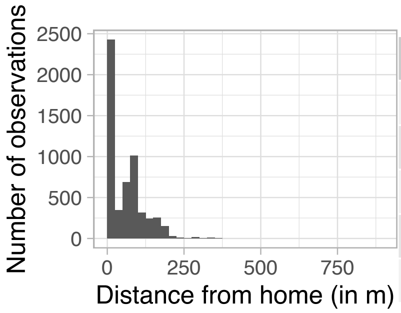

Radius	Residence time	Proportion of total time	Number of visits
50m	47.2 h	49.2%	51
100m	75.8 h	18.5%	54
150m	85.2 h	8.4%	86
200m	91.9 h	1.2%	13
250m	92.4 h	0.7%	3

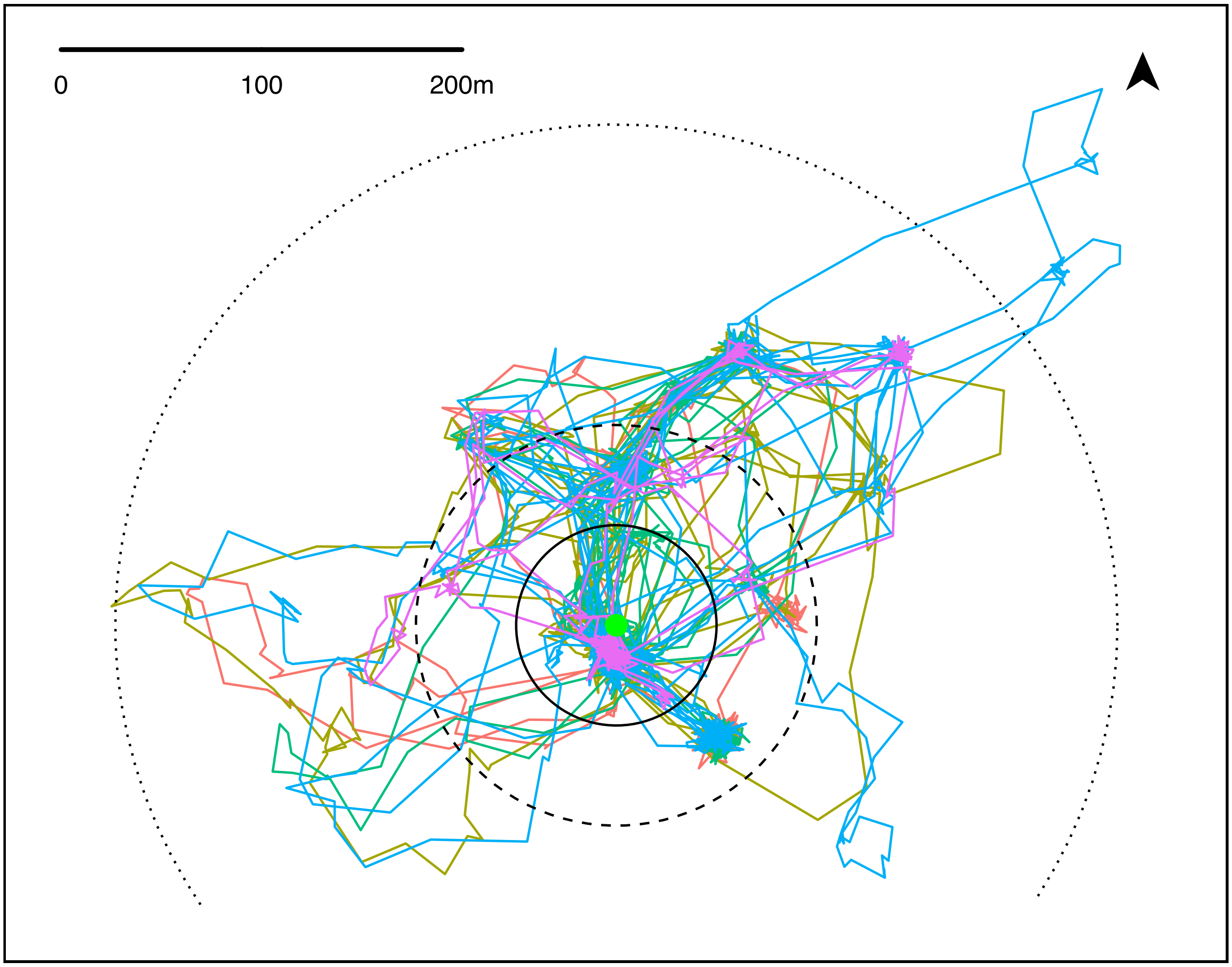

Pig 23

Neighborhood	Neighborhood B
Season	October
Sex	M
Age (months)	13

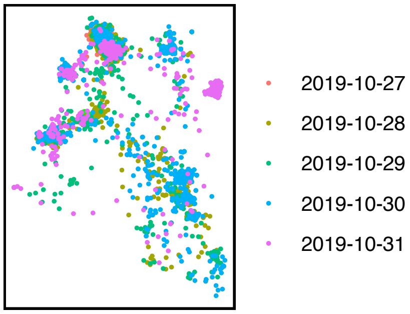

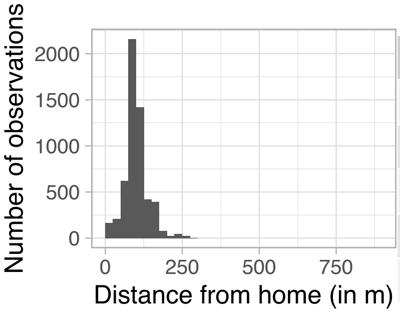

Radius	Residence time	Proportion of total time	Number of visits
50m	6.3 h	93.3%	43
100m	53.6 h	43.1%	222
150m	84.5 h	10.3%	48
200m	92.6 h	1.7%	11
250m	93.8 h	0.5%	6

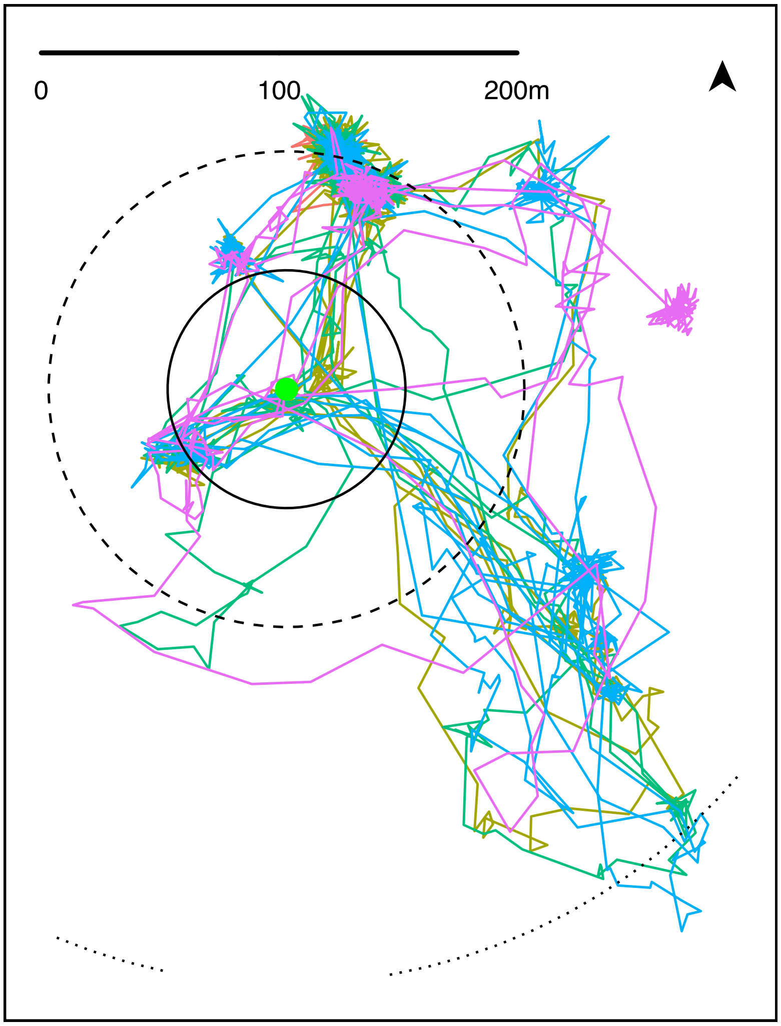

Pig 24

Neighborhood	Neighborhood A
Season	April
Sex	M
Age (months)	NA

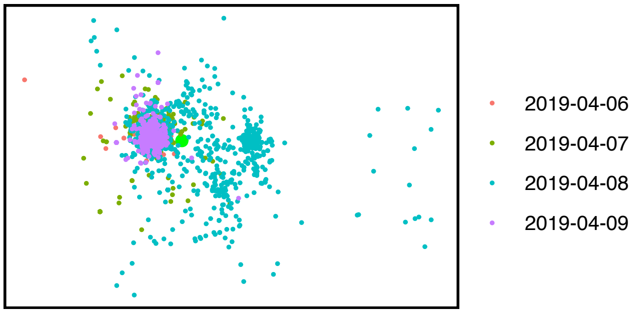

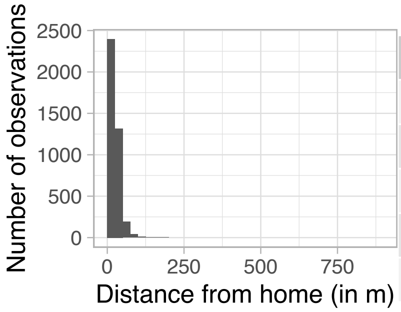

Radius	Residence time	Proportion of total time	Number of visits
50m	69.6 h	7.2%	47
100m	74.6 h	0.5%	5
150m	74.8 h	0.2%	1
200m	75 h	0%	0
250m	75 h	0%	0

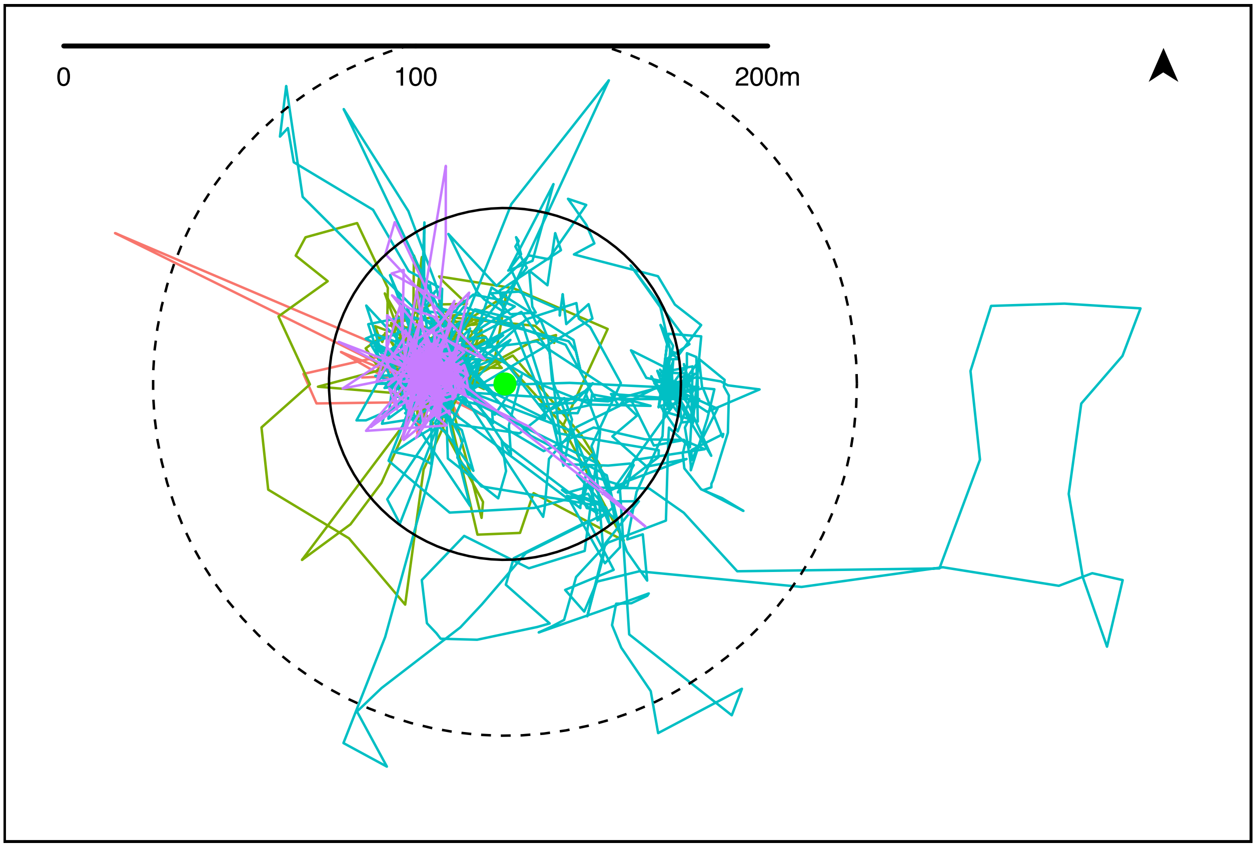

Pig 25

Neighborhood	Neighborhood A
Season	April
Sex	F
Age (months)	4

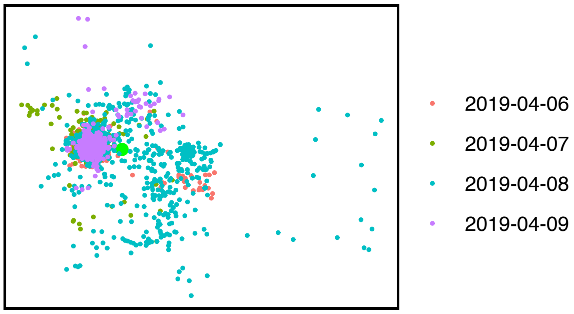

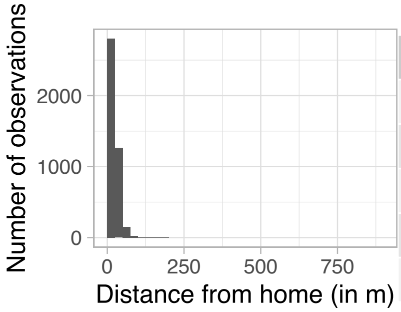

Radius	Residence time	Proportion of total time	Number of visits
50m	70.8 h	4.2%	32
100m	73.6 h	0.4%	2
150m	73.7 h	0.2%	1
200m	73.9 h	0%	0
250m	73.9 h	0%	0

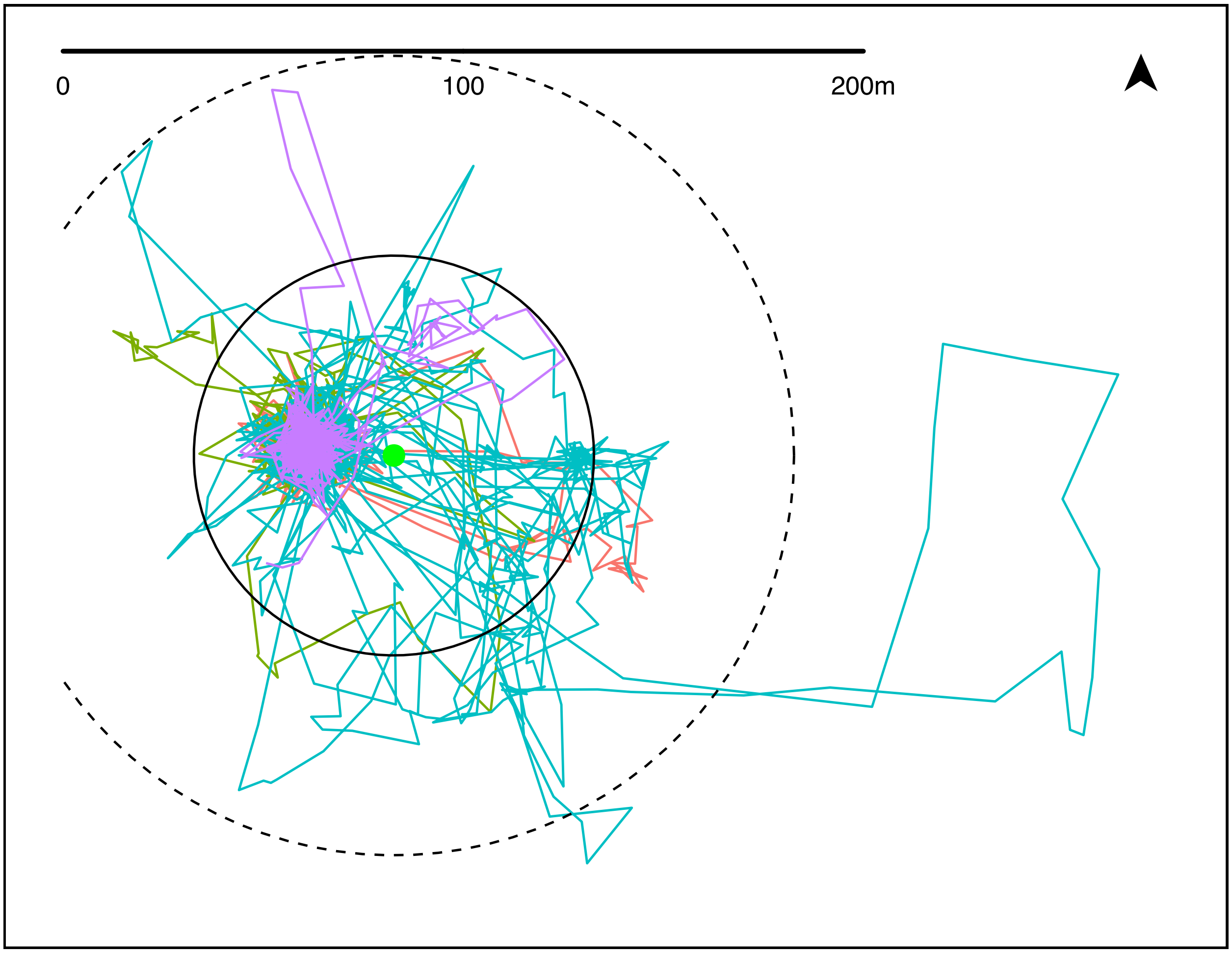

Pig 26

Neighborhood	Neighborhood A
Season	April
Sex	F
Age (months)	NA

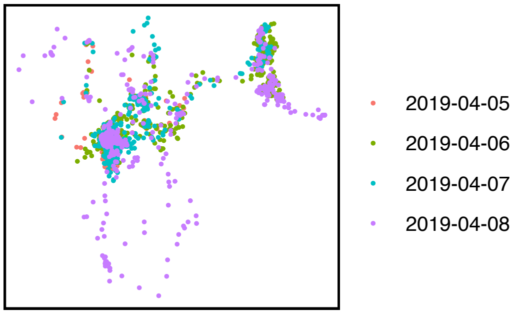

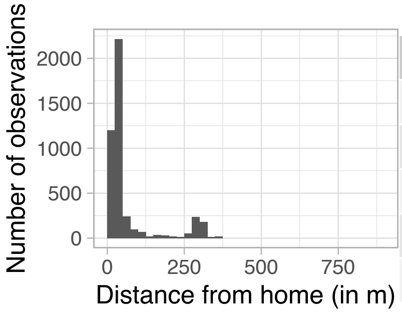

Radius	Residence time	Proportion of total time	Number of visits
50m	58.2 h	23%	77
100m	63.8 h	15.5%	23
150m	65.3 h	13.6%	11
200m	66.4 h	12.1%	9
250m	67 h	11.3%	5

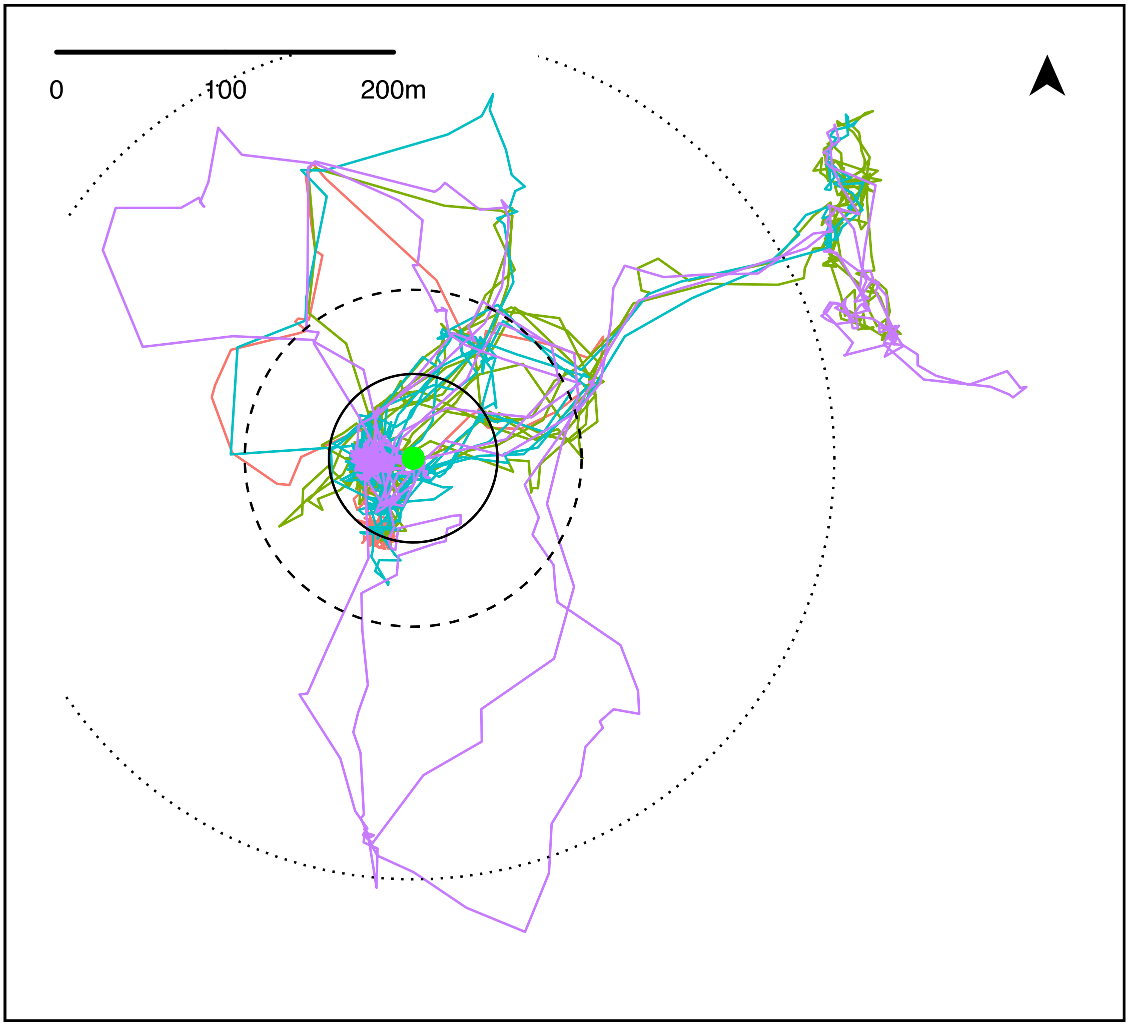

Pig 27

Neighborhood	Neighborhood A
Season	April
Sex	F
Age (months)	10

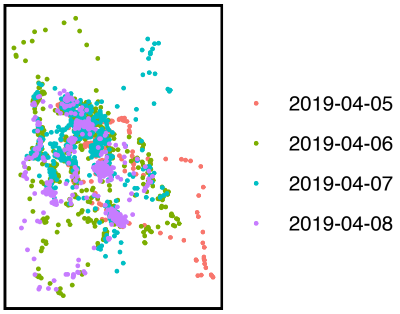

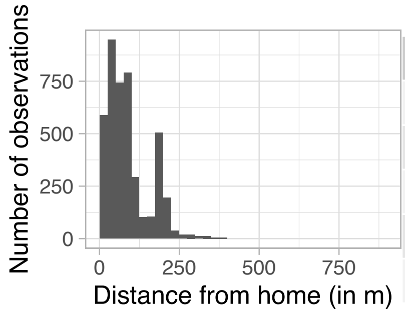

Radius	Residence time	Proportion of total time	Number of visits
50m	25.6 h	65.4%	217
100m	51 h	31.2%	91
150m	57.4 h	22.6%	24
200m	67.9 h	8.4%	42
250m	72.9 h	1.7%	6

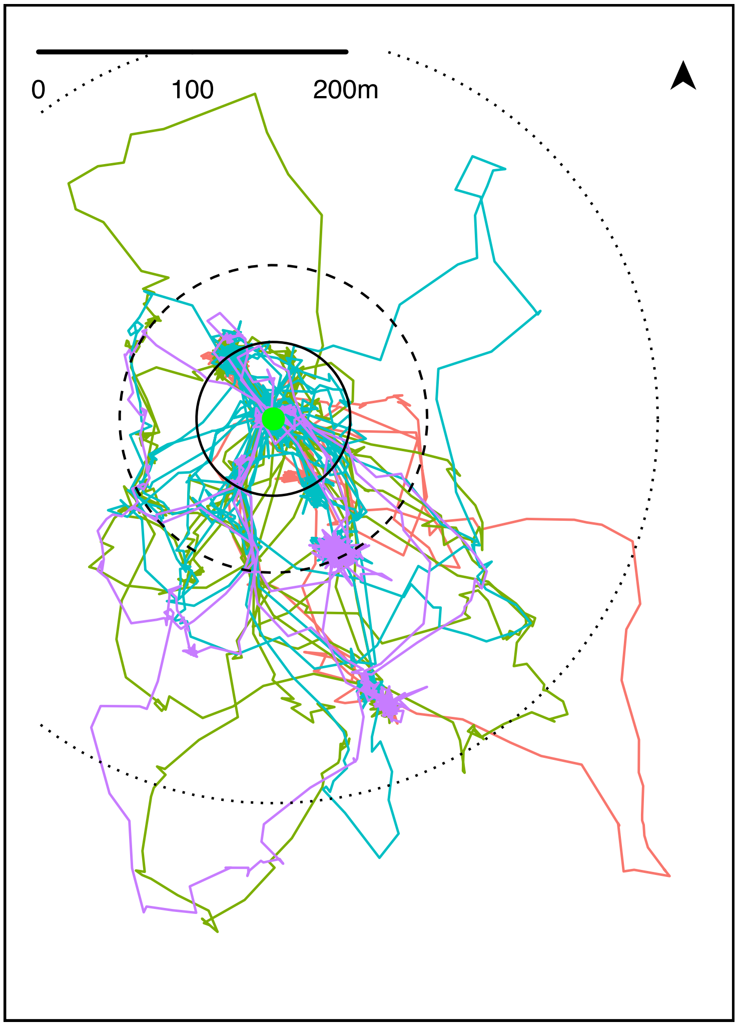

Pig 28

Neighborhood	Neighborhood A
Season	April
Sex	F
Age (months)	10

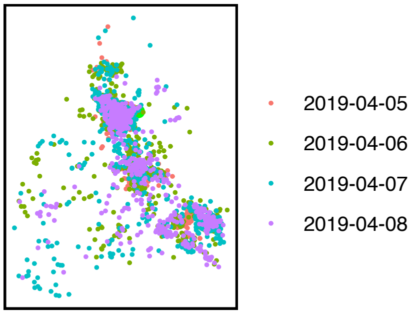

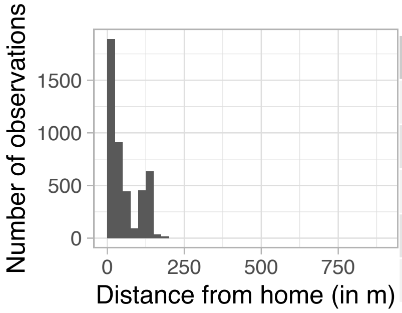

Radius	Residence time	Proportion of total time	Number of visits
50m	45.8 h	37.9%	55
100m	54.7 h	25.8%	19
150m	72.8 h	1.2%	6
200m	73.7 h	0%	0
250m	73.7 h	0%	0

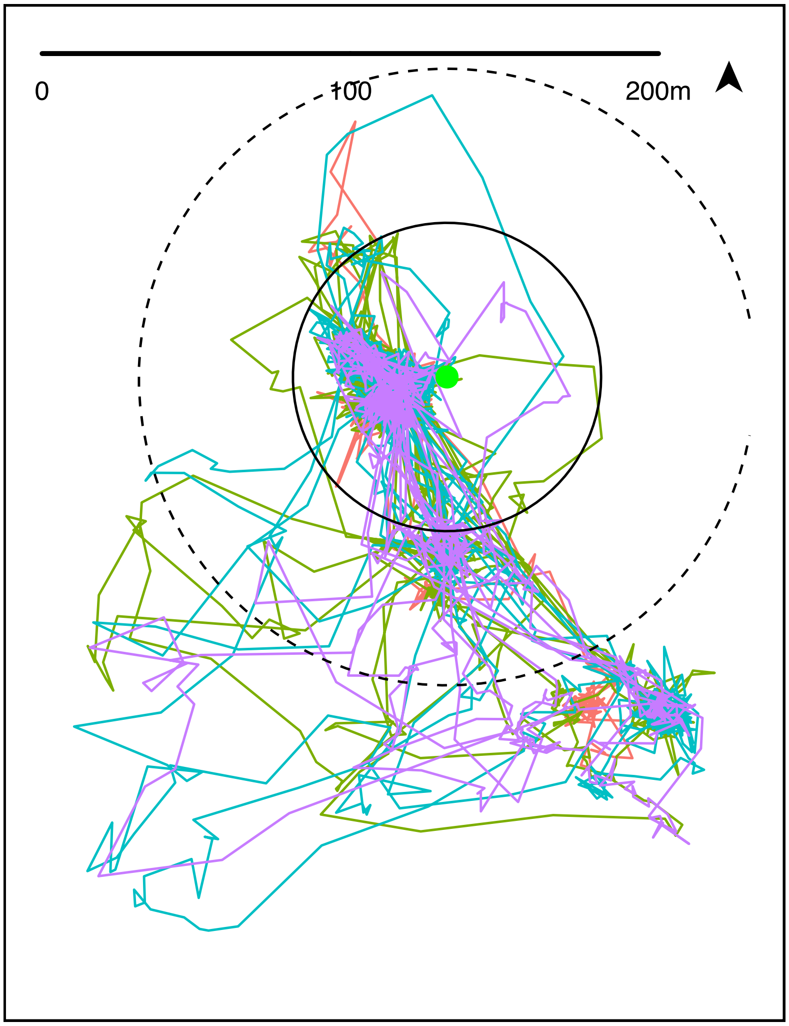

Pig 29

Neighborhood	Neighborhood A
Season	April
Sex	F
Age (months)	10

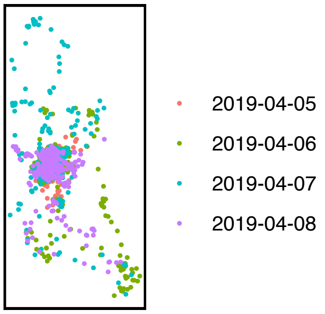

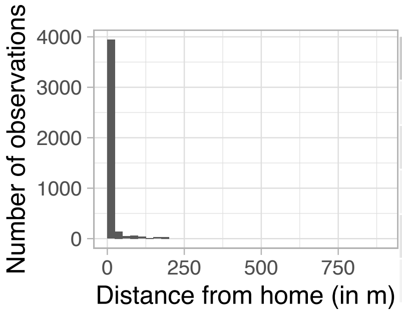

Radius	Residence time	Proportion of total time	Number of visits
50m	69.3 h	5%	16
100m	71.2 h	2.5%	7
150m	72 h	1.4%	4
200m	73 h	0%	0
250m	73 h	0%	0

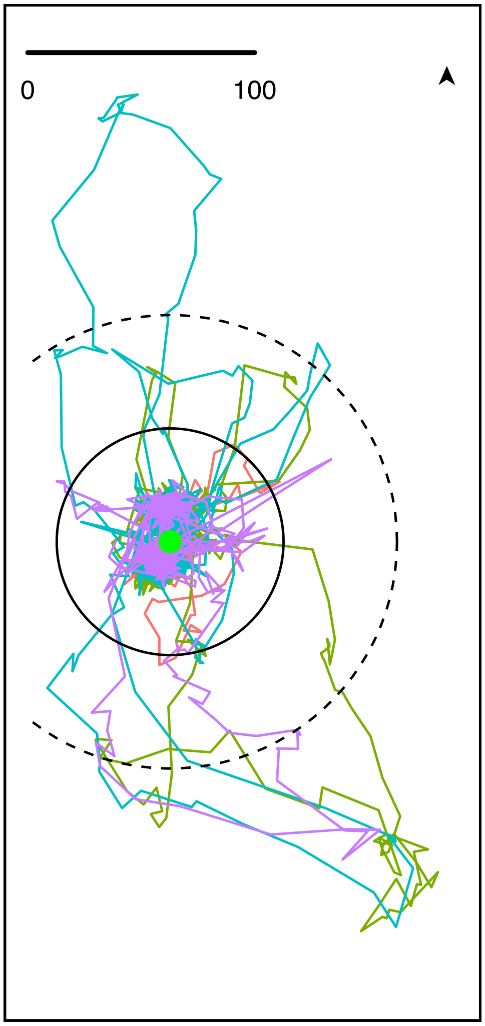

Pig 30

Neighborhood	Neighborhood A
Season	April
Sex	M
Age (months)	9

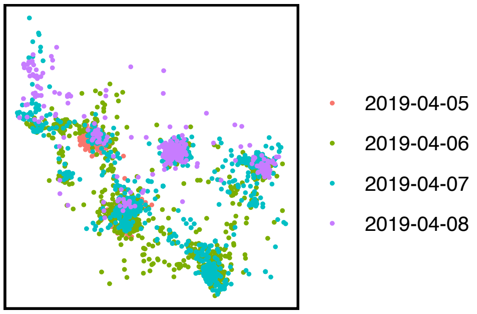

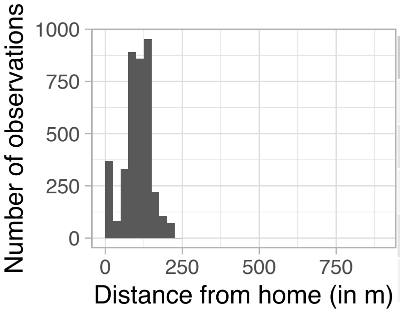

Radius	Residence time	Proportion of total time	Number of visits
50m	7.2 h	88.6%	10
100m	27.9 h	56.3%	50
150m	57.6 h	9.9%	55
200m	62.6 h	1.9%	9
250m	63.9 h	0%	0

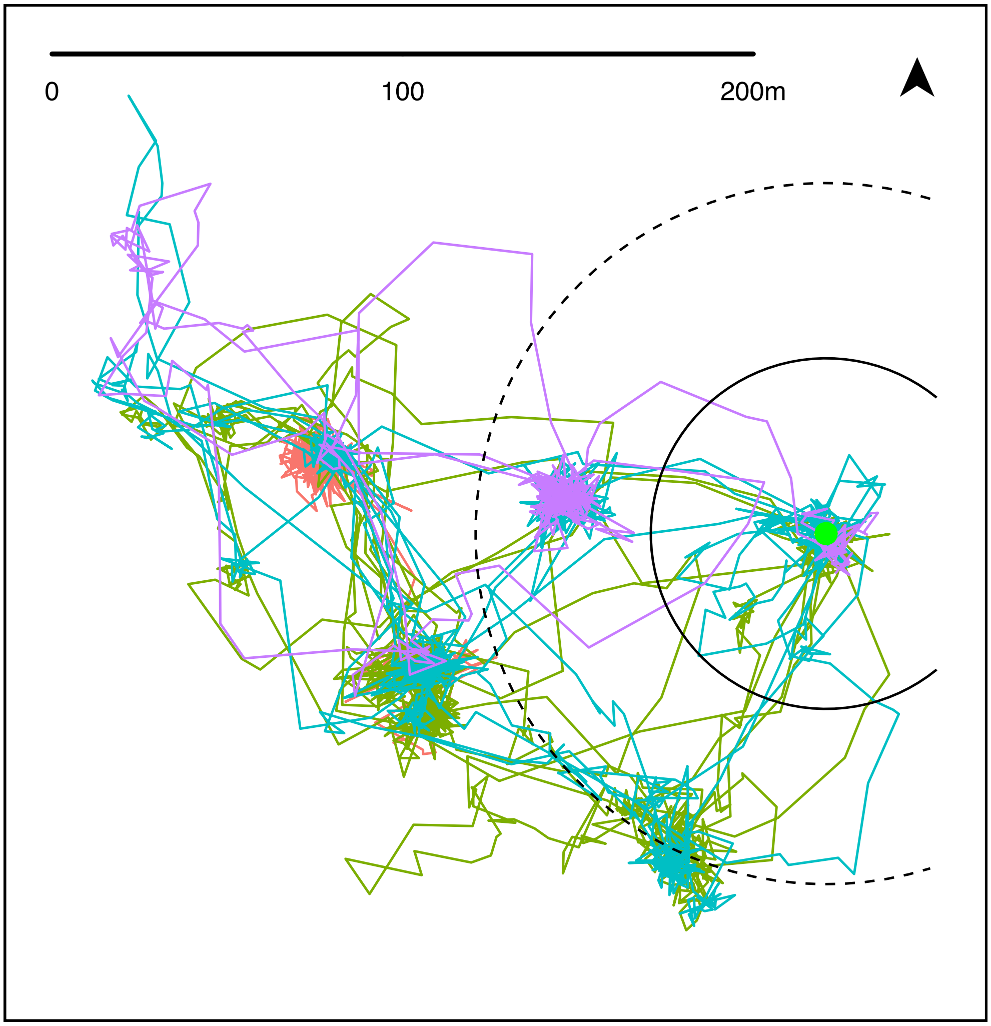

Pig 31

Neighborhood	Neighborhood A
Season	April
Sex	F
Age (months)	10

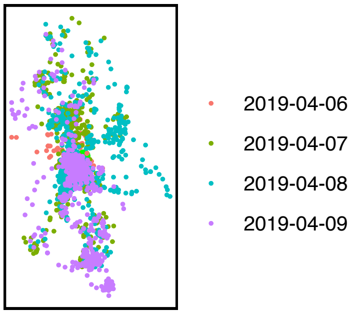

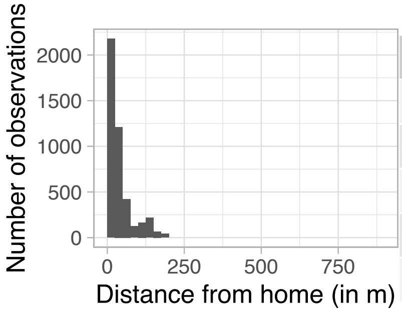

Radius	Residence time	Proportion of total time	Number of visits
50m	60.4 h	22.5%	52
100m	69.8 h	10.4%	29
150m	76.2 h	2.3%	31
200m	78 h	0%	0
250m	78 h	0%	0

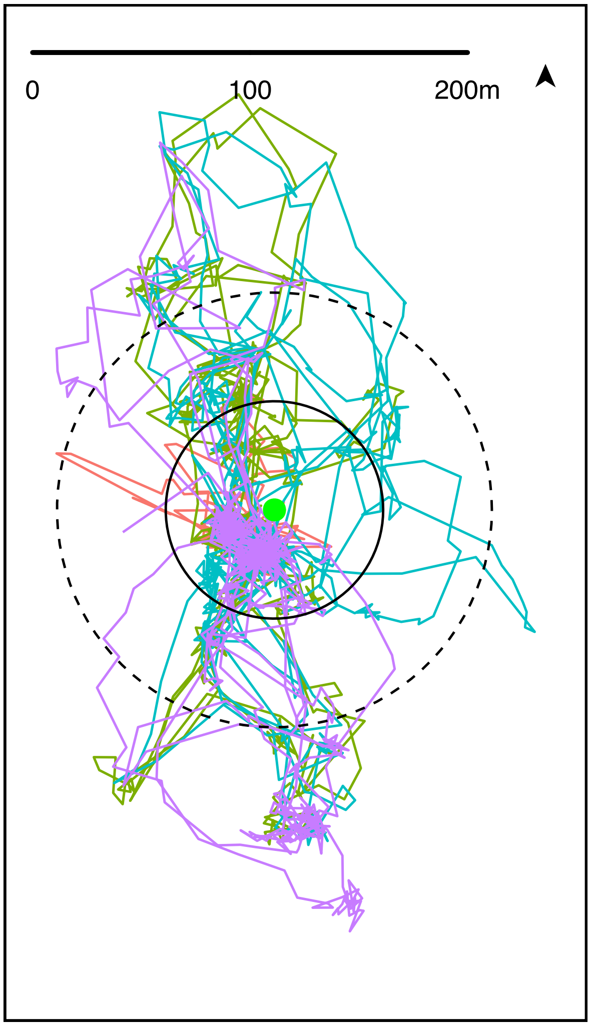

Pig 32

Neighborhood	Neighborhood A
Season	April
Sex	M
Age (months)	7

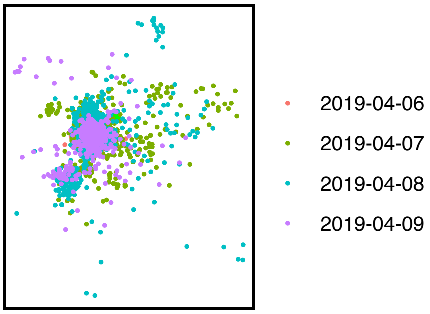

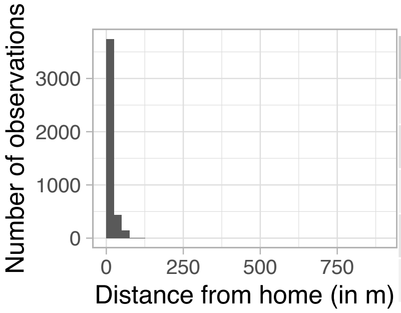

Radius	Residence time	Proportion of total time	Number of visits
50m	69.5 h	3.4%	36
100m	71.9 h	0.1%	2
150m	71.9 h	0%	0
200m	71.9 h	0%	0
250m	71.9 h	0%	0

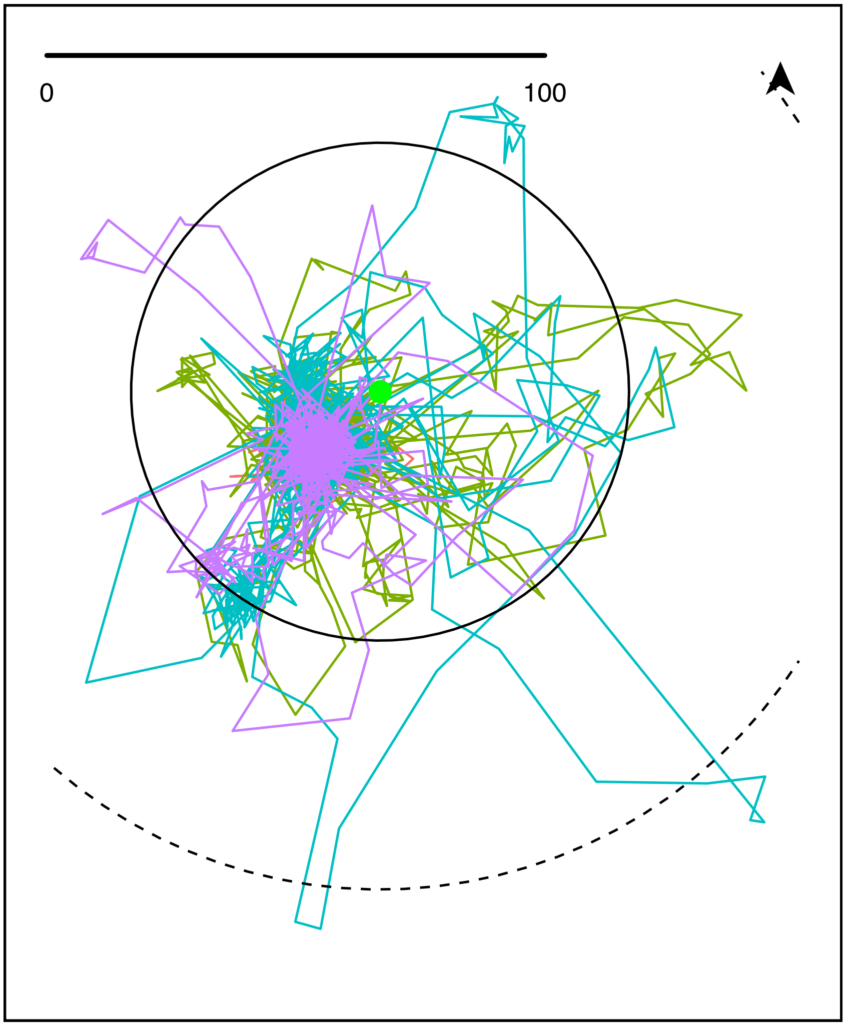

Pig 33

Neighborhood	Neighborhood B
Season	April
Sex	F
Age (months)	13

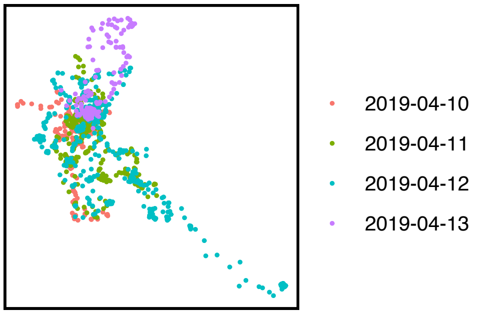

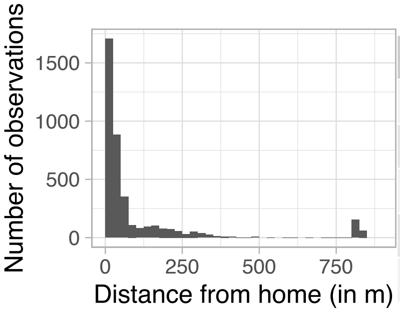

Radius	Residence time	Proportion of total time	Number of visits
50m	43.6 h	35.2%	71
100m	51.5 h	23.4%	21
150m	55.2 h	18%	19
200m	58.1 h	13.6%	23
250m	60.3 h	10.3%	8

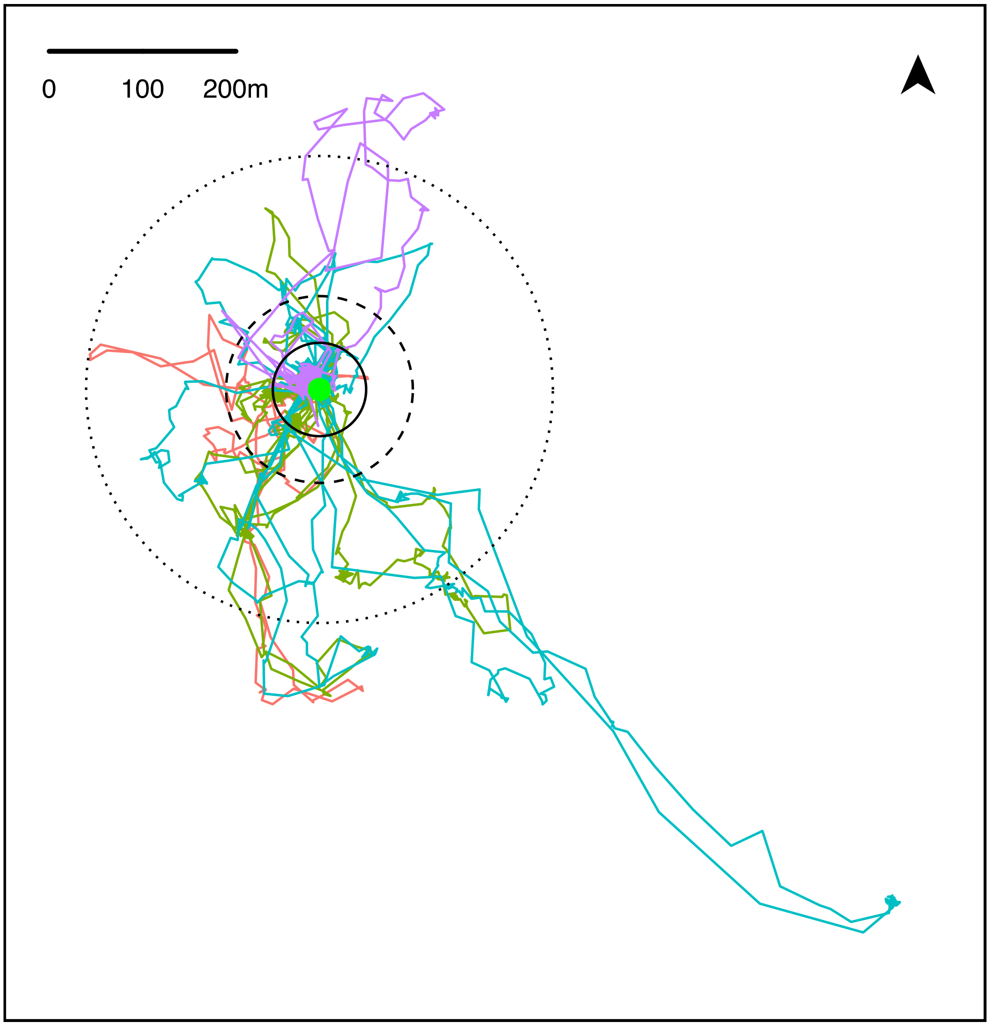

Pig 34

Neighborhood	Neighborhood B
Season	April
Sex	F
Age (months)	11

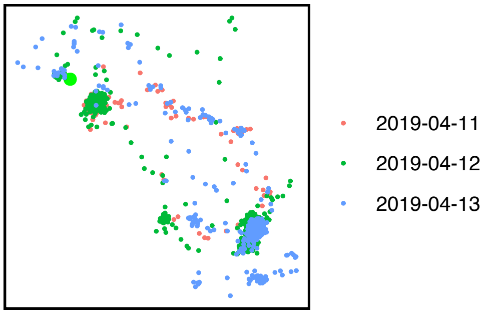

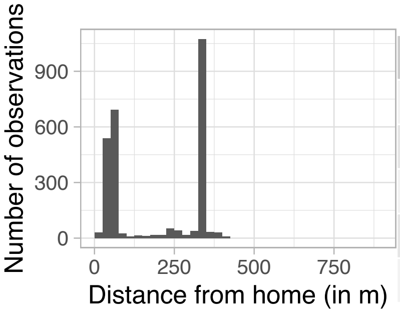

Radius	Residence time	Proportion of total time	Number of visits
50m	9.5 h	79%	142
100m	21.7 h	51.9%	5
150m	22.2 h	50.8%	5
200m	22.7 h	49.8%	5
250m	23.9 h	47.1%	9

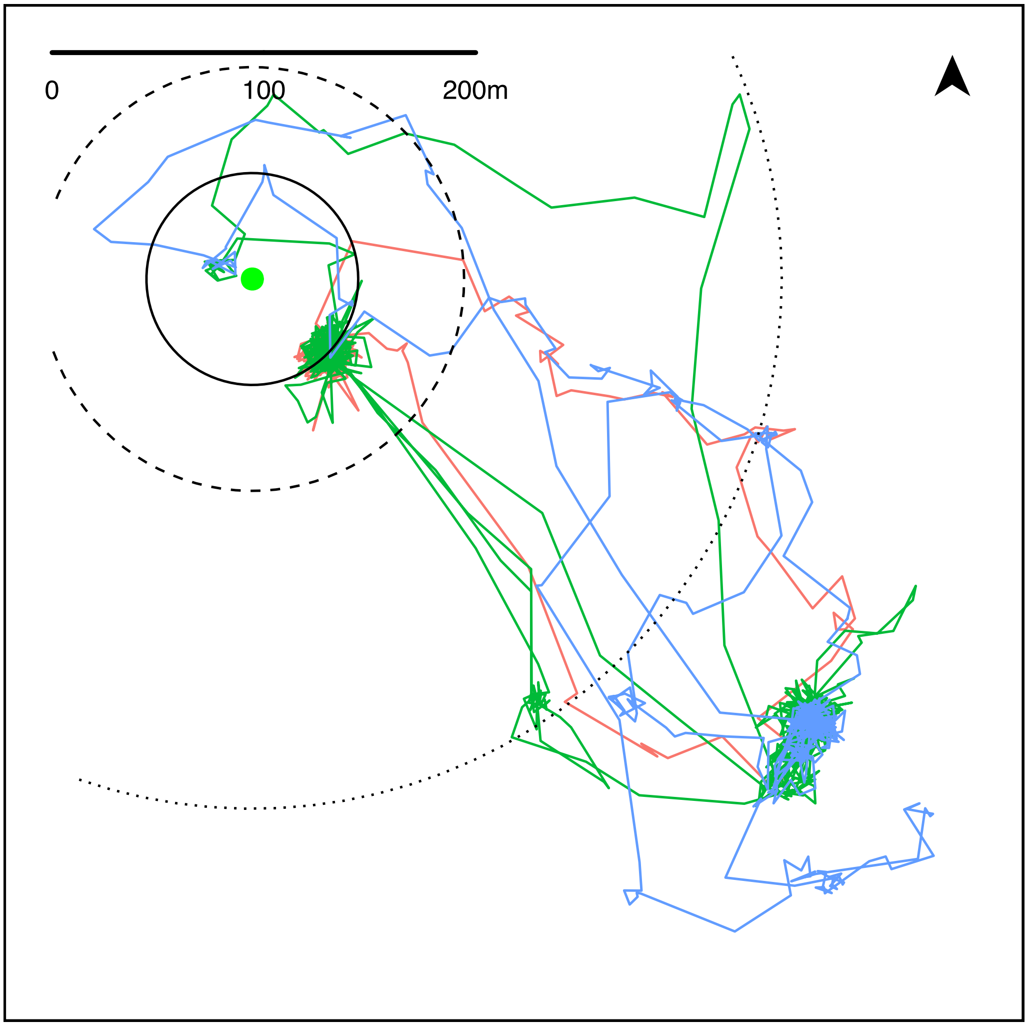

Pig 35

Neighborhood	Neighborhood B
Season	April
Sex	F
Age (months)	54

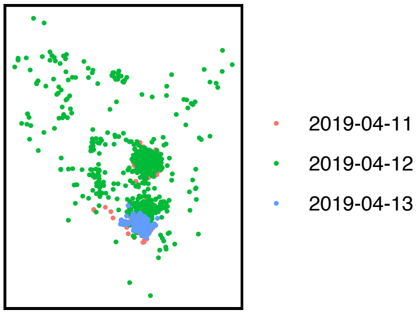

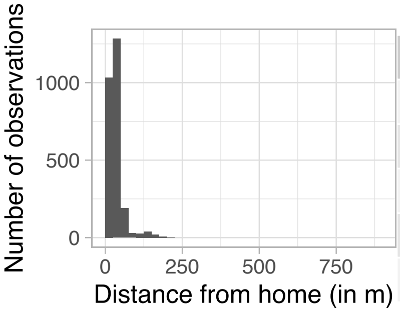

Radius	Residence time	Proportion of total time	Number of visits
50m	39.4 h	11.1%	92
100m	42.7 h	3.6%	3
150m	43.8 h	1.1%	4
200m	44.3 h	0.1%	1
250m	44.3 h	0%	0

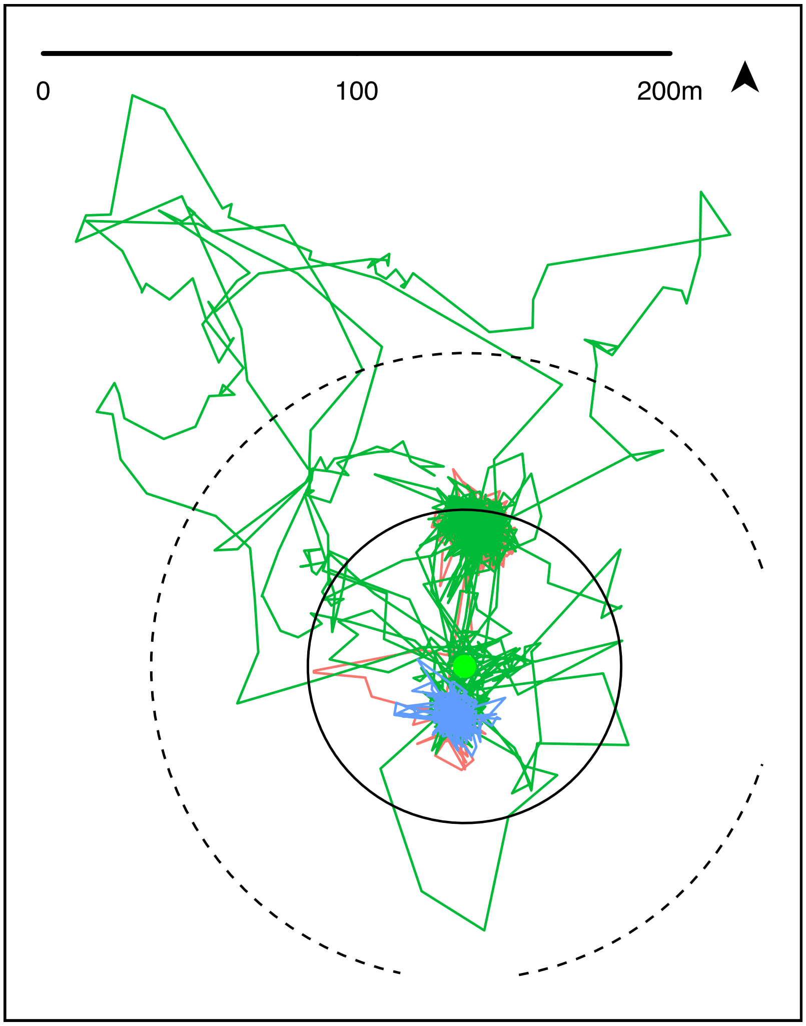

Pig 36

Neighborhood	Neighborhood B
Season	April
Sex	F
Age (months)	33

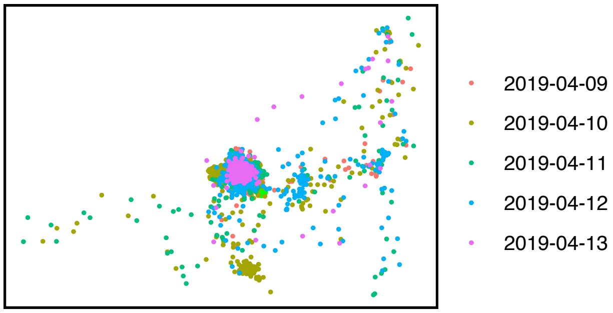

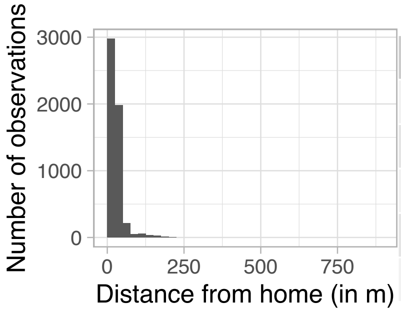

Radius	Residence time	Proportion of total time	Number of visits
50m	84.3 h	7.2%	22
100m	88.7 h	2.4%	16
150m	90.2 h	0.7%	10
200m	90.8 h	0%	1
250m	90.8 h	0%	0

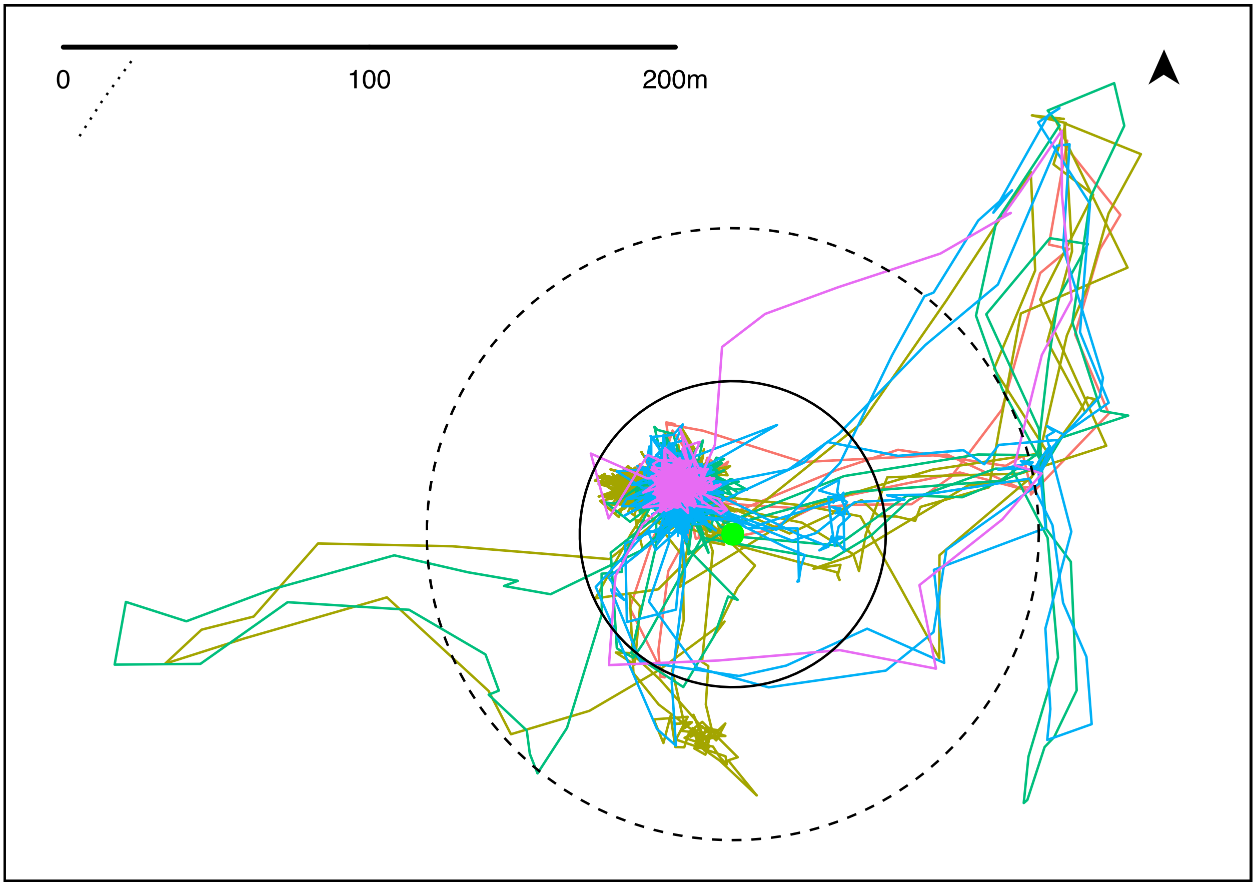

Pig 37

Neighborhood	Neighborhood B
Season	April
Sex	F
Age (months)	60

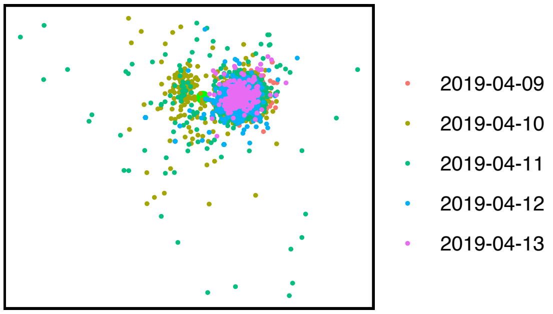

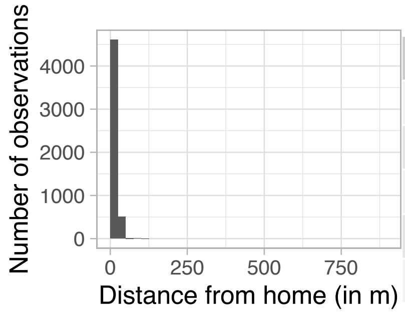

Radius	Residence time	Proportion of total time	Number of visits
50m	87.6 h	0.4%	6
100m	87.9 h	0%	1
150m	87.9 h	0%	0
200m	87.9 h	0%	0
250m	87.9 h	0%	0

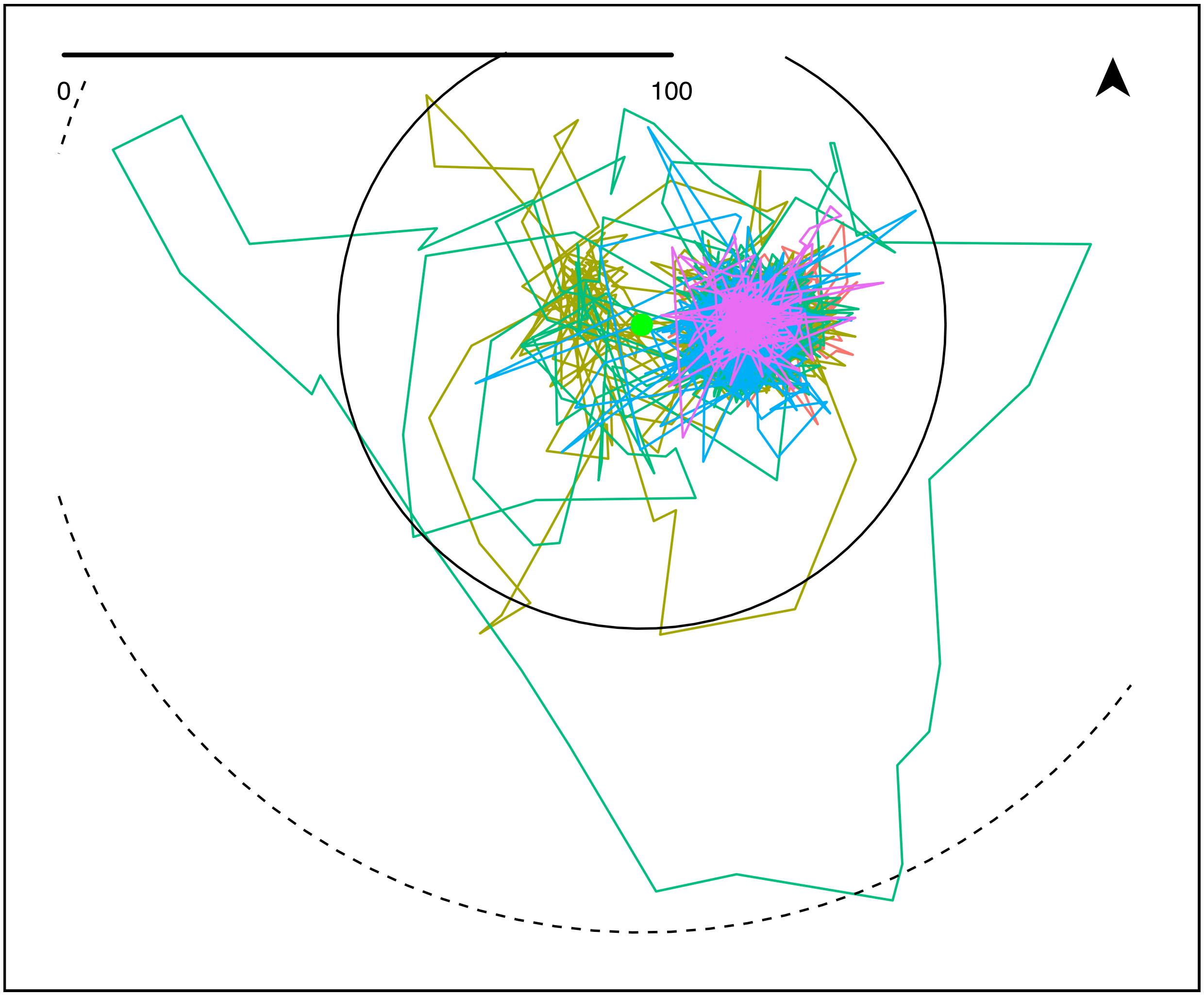

Pig 38

Neighborhood	Neighborhood B
Season	April
Sex	F
Age (months)	15

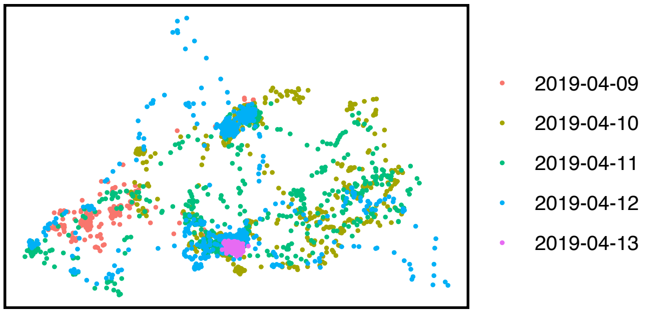

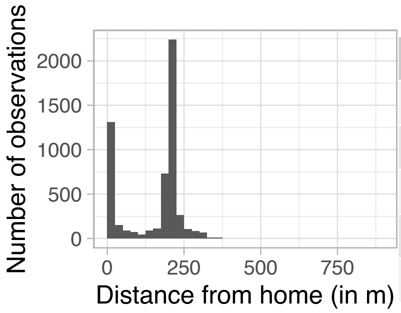

Radius	Residence time	Proportion of total time	Number of visits
50m	25 h	72.3%	20
100m	27.6 h	69.3%	16
150m	29.8 h	66.9%	18
200m	43.8 h	51.4%	46
250m	85.6 h	5%	16

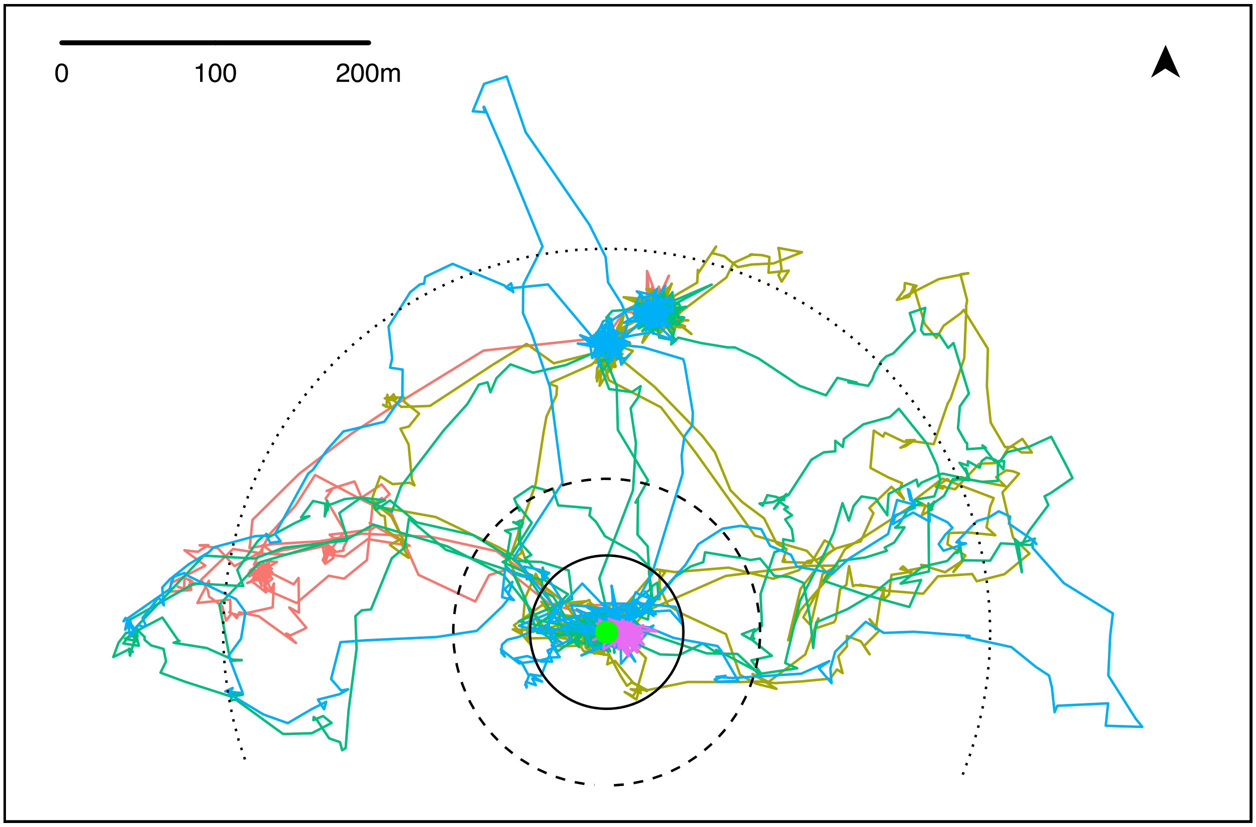

Pig 39

Neighborhood	Neighborhood B
Season	April
Sex	F
Age (months)	28

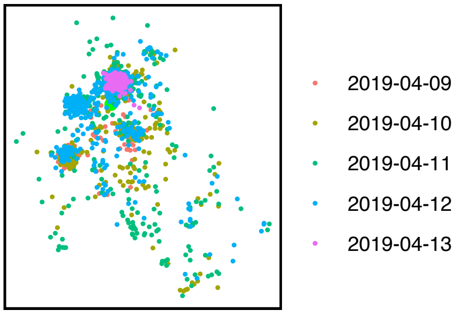

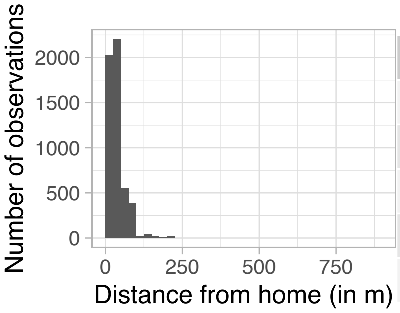

Radius	Residence time	Proportion of total time	Number of visits
50m	71.2 h	20%	44
100m	86.9 h	2.5%	12
150m	88.1 h	1.2%	8
200m	88.7 h	0.5%	5
250m	89.1 h	0%	0

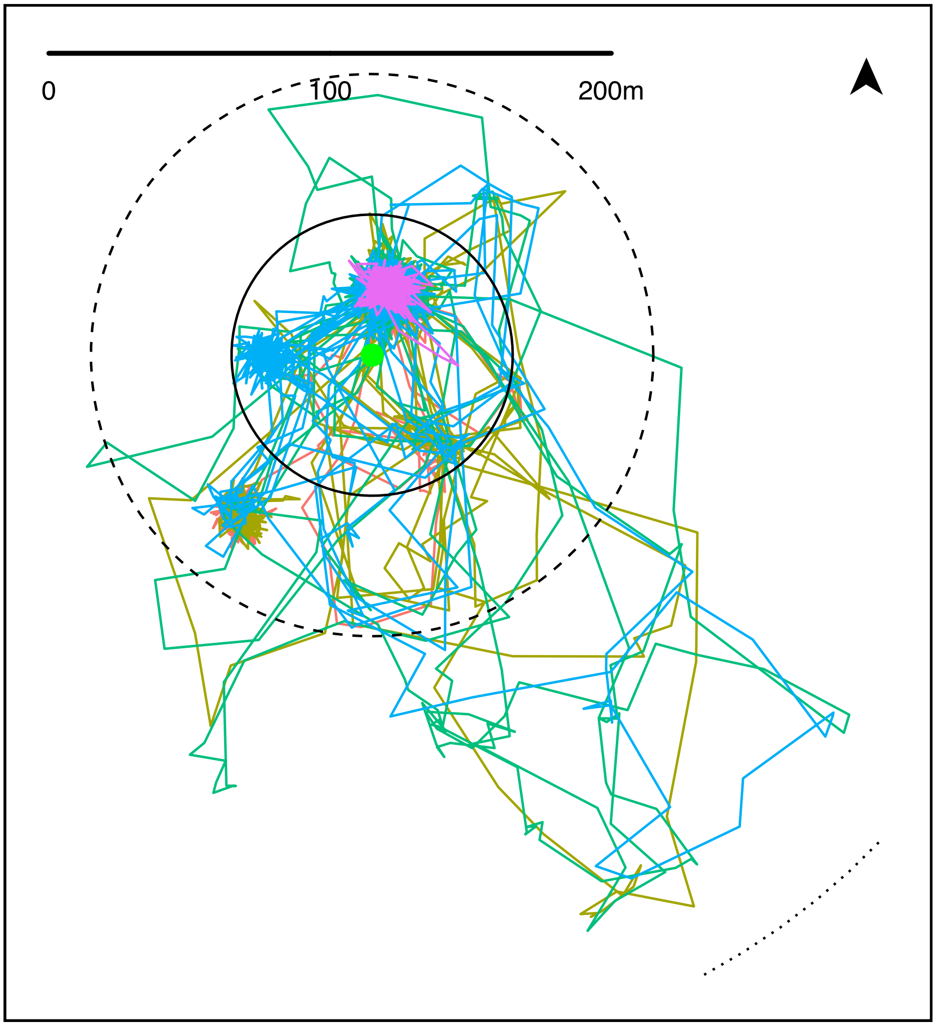

Pig 40

Neighborhood	Neighborhood B
Season	April
Sex	F
Age (months)	10

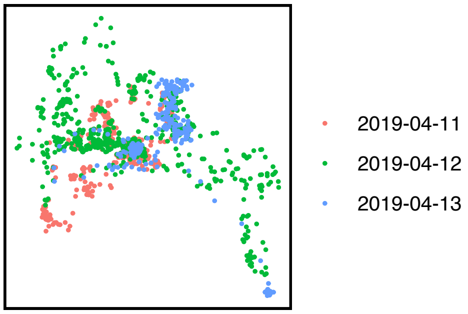

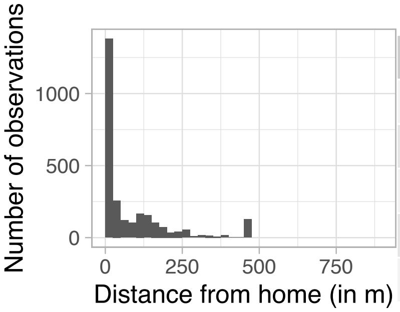

Radius	Residence time	Proportion of total time	Number of visits
50m	27.7 h	39.2%	21
100m	31.4 h	31.1%	24
150m	36.9 h	19%	23
200m	39.9 h	12.4%	11
250m	41.2 h	9.5%	9

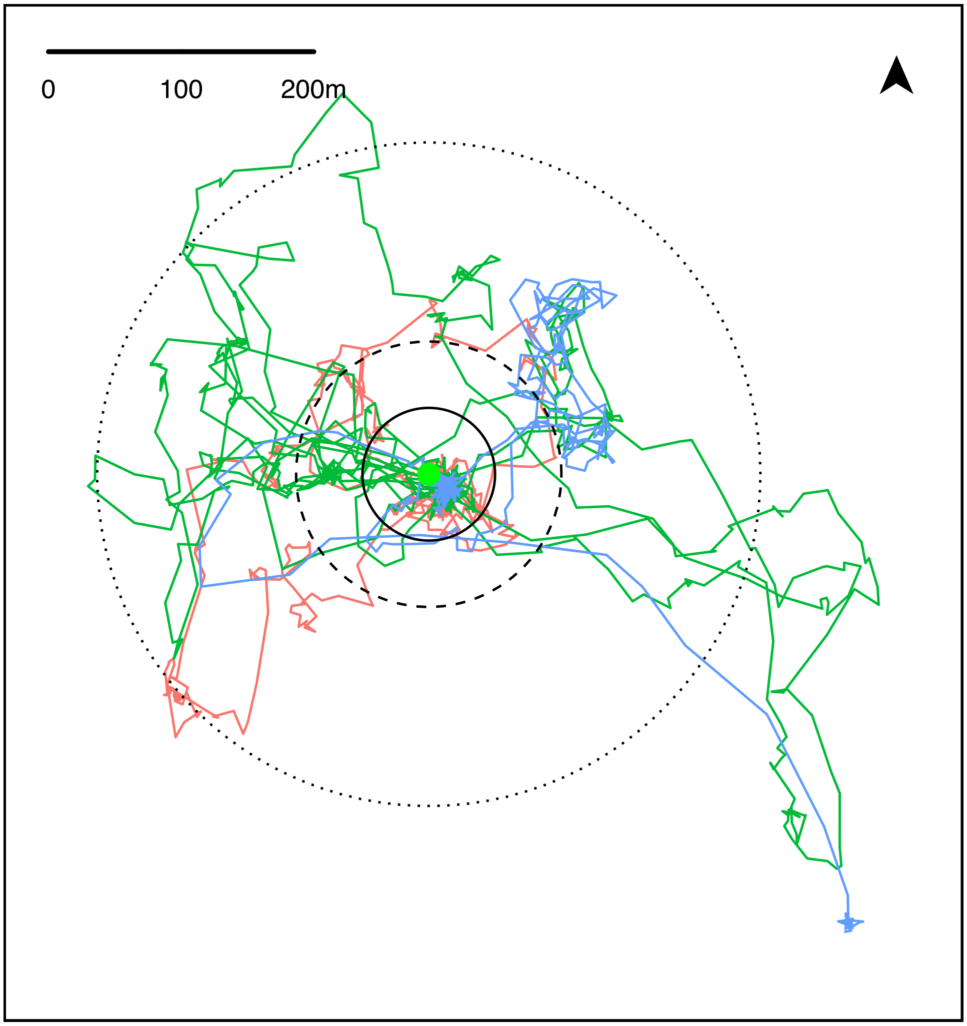

Pig 41

Neighborhood	Neighborhood B
Season	April
Sex	M
Age (months)	6

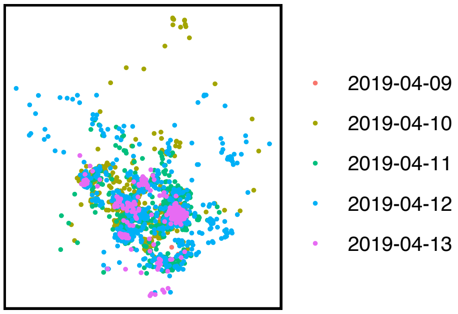

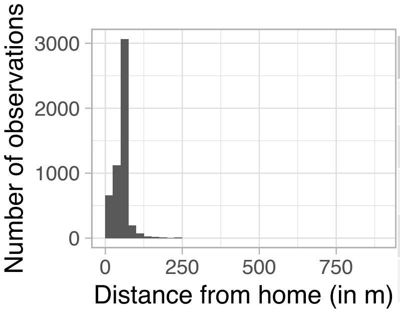

Radius	Residence time	Proportion of total time	Number of visits
50m	29.8 h	65.7%	108
100m	84.7 h	2.7%	19
150m	86.3 h	0.8%	5
200m	86.8 h	0.3%	2
250m	87 h	0%	0

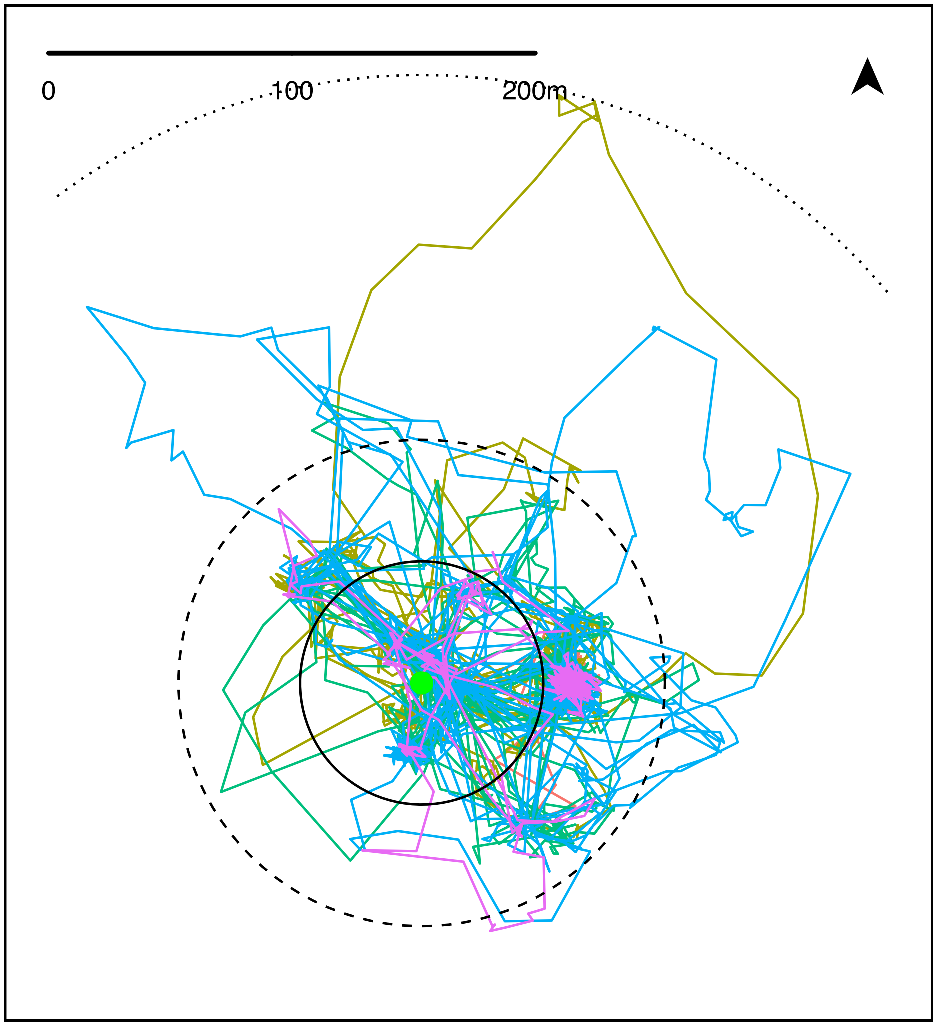

Pig 42

Neighborhood	Neighborhood B
Season	April
Sex	F
Age (months)	3

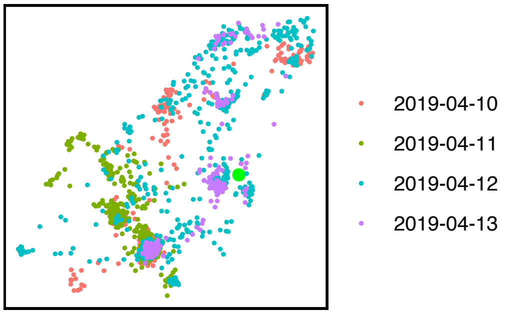

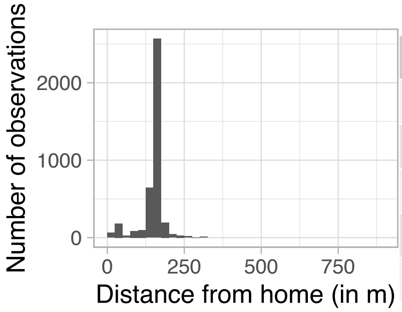

Radius	Residence time	Proportion of total time	Number of visits
50m	4.6 h	93.5%	7
100m	6.4 h	90.8%	20
150m	18.9 h	73%	213
200m	68.4 h	2.6%	14
250m	69.6 h	0.8%	3

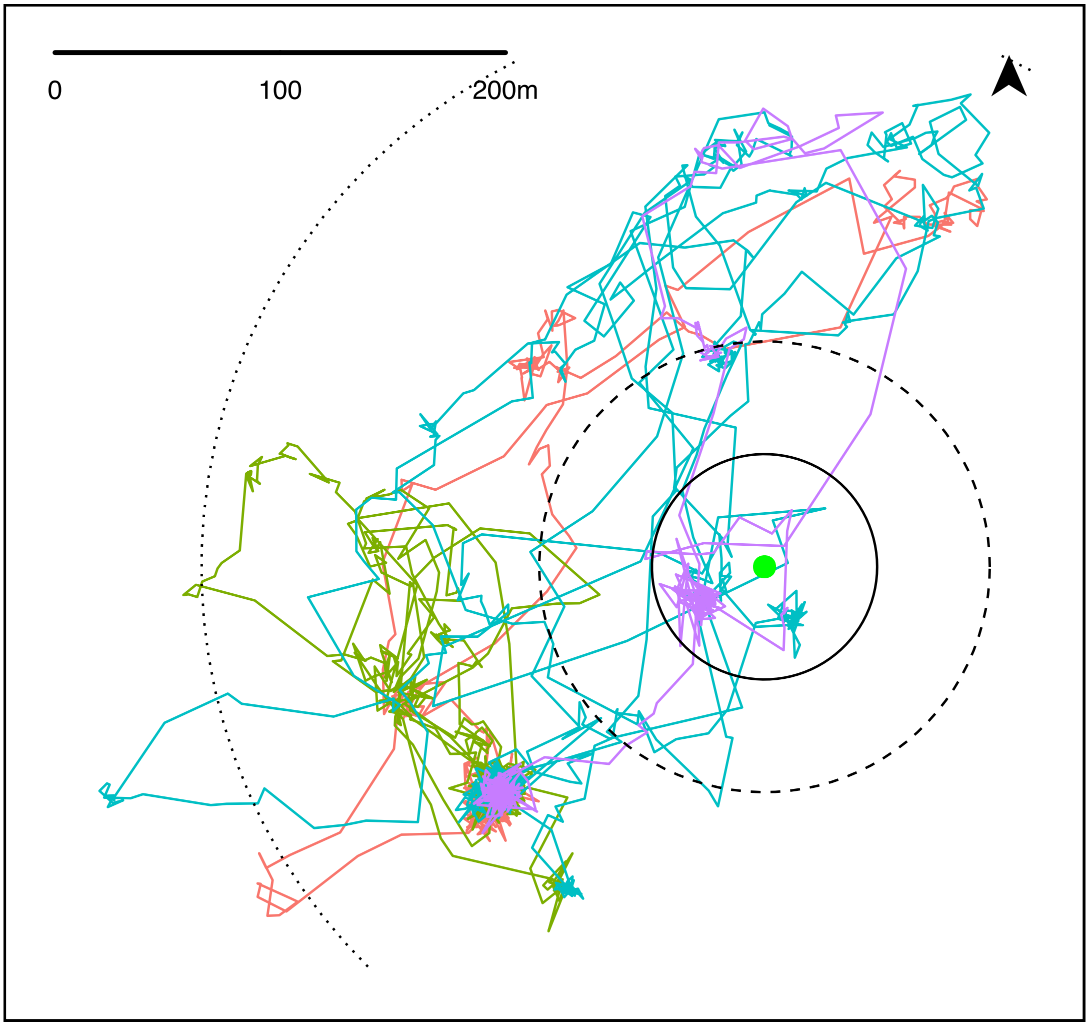

Pig 43

Neighborhood	Neighborhood B
Season	April
Sex	F
Age (months)	10

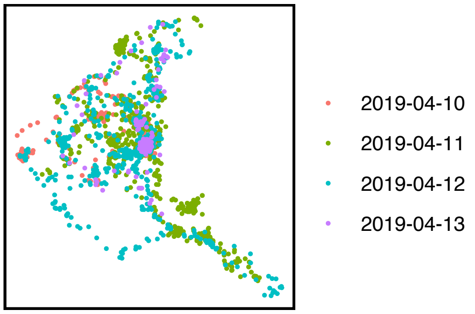

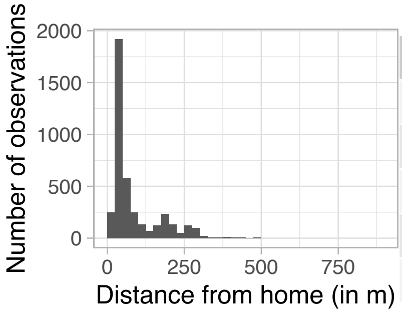

Radius	Residence time	Proportion of total time	Number of visits
50m	37.4 h	45.6%	172
100m	51.1 h	25.7%	24
150m	54.5 h	20.7%	28
200m	60.6 h	11.9%	17
250m	63.7 h	7.4%	19

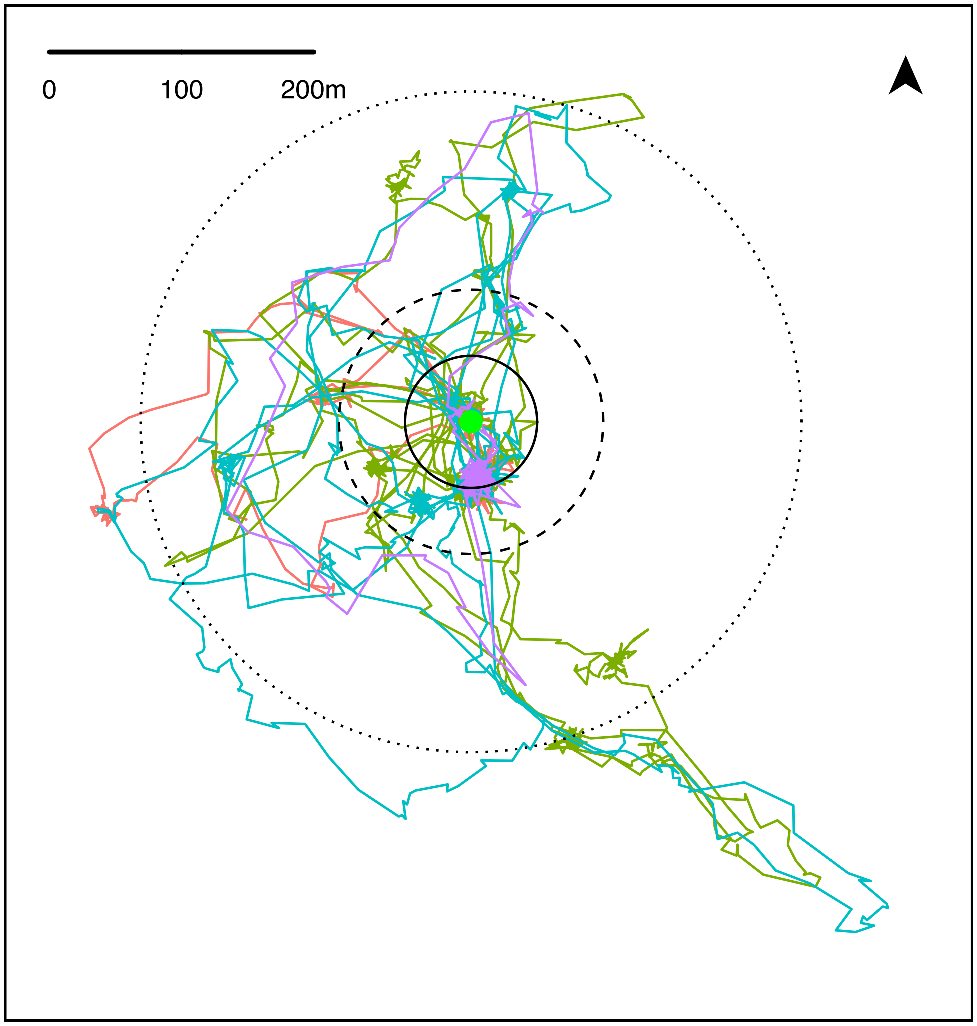

Supplement: Supplementary file 2 — Additional file 2. GPS tracks and summary data on individual pigs. [file 13071_2022_5264_MOESM2_ESM.pdf]
